# Dual modulation of toxic risks and functional benefits: How traditional practices enable safe consumption of toxic *Rhododendron decorum* Franch. in ethnic cuisine

**DOI:** 10.3389/fpls.2026.1778014

**Published:** 2026-06-09

**Authors:** Yongqian Gao, Weiwei Liu, Ling Wang, Zhongyu Fan, Kaiye Yang, Liyuan He, Xinchun Mo

**Affiliations:** 1 College of Forestry, Yunnan Forestry Technological College, Kunming, China; 2 College of Landscape Architecture and Horticulture, Southwest Forestry University, Landscape Architecture & Horticulture Faculty, Kunming, China; 3 Lijiang Forest Biodiversity National Observation and Research Station, Kunming Institute of Botany, Chinese Academy of Science, Kunming, China; 4 School of Life Science, Lijiang Normal University, Lijiang, China; 5 School of Medicine and Health, Kunming Vocational University of Science and Technology, Kunming, China

**Keywords:** comparative metabolome, dual modulations, edible corollas, *Rhododendron decorum* Franch., toxic risks and functional benefits, traditional ethnic cuisine, transcriptome-metabolome correlation analysis

## Abstract

**Introduction:**

The corollas of *Rhododendron decorum* Franch. have been used as a traditional ethnic food by the Bai nationality in Yunnan, China for centuries, with a well-preserved custom of removing the androecium and gynoecium prior to soaking, drying, and cooking. However, the molecular basis, nutritional value, and edible safety of this long-standing practice remain largely uncharacterized, limiting the development and utilization of this characteristic plant resource.

**Methods:**

We integrate transcriptomic, metabolomic, and comparative metabolomic analyses to systematically characterize the molecular and metabolic profiles of distinct floral organs, geographically distinct populations, and fresh versus traditionally processed edible corollas of *R. decorum* Franch.

**Results and Discussion:**

3,463 differentially expressed genes (DEGs) and 165 differentially accumulated metabolites (DAMs) between corollas and androecium/gynoecium from Lijiang populations, and 3,229 DEGs and 145 DAMs from Heqing populations were identified, respectively. The flavonoid biosynthesis pathway showed the greatest divergence, with potentially risky flavonoids highly enriched in the androecium and gynoecium. Regional comparison revealed only the glycerophospholipid metabolism pathway was significantly co-enriched with DEGs and DAMs between edible corollas from the two regions, with no notable differences in other edible-related functional pathways. Traditional processing stably retained flavor-related L-histidine and S-(4-Methylthiobutylthiohydroximoyl)-L-cysteine, upregulated functional components p-coumaroyl quinic acid and taxifolin, and significantly downregulated potentially harmful flavonoids including (-)-epigallocatechin and myricetin.

**Conclusion:**

This study systematically reveals the molecular basis of the Bai nationality’s traditional edible practice of *R. decorum* Franch., demonstrating that removing the androecium/gynoecium and traditional processing can effectively reduce edible risks while retaining flavor and functional active components. These findings provided critical scientific support for the food safety assessment.

## 1 Introduction

The decorative use of flowers can be traced back to approximately 3,000 BC (Matyjaszczyk and Śmiechowska, 2019). Their role as food dates back thousands of years, with examples such as capers, artichokes, broccoli, and cauliflower (Nicolau and Gostin, 2016). At present, flowers are integrated into a wide range of culinary traditions in Europe, Asia, and the Middle East, which is due to their appealing aroma, distinctive taste, and high nutritional value (Chetia et al., 2025; Mlcek and Rop, 2011; Motti et al., 2022). Among the diverse applications for humans, edible flowers are harvested from numerous plants and employed in a wide range of forms, such as stand-alone dishes that are prepared and served directly, salads, soups, beverages, desserts, confections, and jellies. They are also used as flavor enhancers for cakes, marmalades, and yogurts (Takahashi et al., 2020). Yunnan province, well-known in China for its biodiversity, is the habitat of various ethnic minorities. These minorities possess a unique understanding of edible flowers, affected by their dietary habits and the regional plant distribution (Zhang et al., 2023b). For instance, the Dai and Hani ethnic minorities, predominantly residing in southern Yunnan, exhibit a preference for the flowers of *Zingiber* and *Musa* plants, which are abundant in this area. Once pickled, these flowers develop a distinctive sour flavor and are commonly utilized in mixed cold dishes (Zhang et al., 2023a). Conversely, the Bai ethnic minority, primarily living in northwestern Yunnan, holds the belief that darker-colored flowers are more prone to being toxic. Consequently, they favor lighter-colored flowers, such as those of *Pyrus* and *Rhododendron* plants. These flowers were typically incorporated into meat-based side dishes or cooked independently (He et al., 2015; Pinakin et al., 2020). *Rhododendron decorum* Franch., a perennial evergreen shrub or small tree belonging to the genus *Rhododendron* of the Ericaceae family, is mainly distributed in the subalpine areas with an altitude of 1,000–3,600 m in western Yunnan, western Sichuan, southern Xizang, and northern Guizhou in China. It has large, white, or pale pink corollas with a faint fragrance, and its flowering period is from April to June. As a representative edible flower resource in southwest China, its corollas have been used as a traditional edible ingredient by local ethnic minorities for hundreds of years (Shi et al., 2021).

Nevertheless, it has been reported that the majority of *Rhododendron* species are toxic and not suitable for consumption. Among the identified toxic chemicals from genus *Rhododendron*, grayanotoxin and certain flavonoids have been found to accumulate significantly in their flowers, leaves, and even the honey produced by bees, posing a potential danger to both herbivores and humans (Baral et al., 2022; Paudel et al., 2022; Schregel et al., 2024). The toxic mechanism of grayanotoxin has been demonstrated to involve its interaction with sodium channel activators. This interaction leads to abnormal sodium ion function and the activation of voltage-gated sodium channels (VGSCs) (Yang et al., 2022). Additionally, certain flavonoids have been demonstrated to induce the attenuation of mitochondrial membrane potential in a time- and dose-dependent manner, ultimately leading to apoptosis (Liu et al., 2015). Although it has been reported that the *Rhododendron* species contain toxic chemicals for human, some of them are regarded as potential drugs or nutritional food additives by the local people in Yunnan province, China. *R. decorum*, a potential fundamental source plant for the development of natural medicines and health products, is rich in flavonoids, such as taxifolin, gallocatechin, and myricetin (Liu et al., 2024a). It has been reported that when *Rhododendron* extracts are added to the fermentation process, beers can effectively enhance the aroma, taste, and color. Although the extracts contain tannins, saponins, alkaloids, and flavonoids (Mehra et al., 2020), the Bai nationality traditionally consume the corollas of *R. decorum*, despite the presence of toxic substances in them (Liu et al., 2024a, 2024). During the processing of fresh corollas of *R. decorum*, the androecium/gynoecium are typically removed, while the corollas are retained. The corollas are then soaked and rinsed in clean water for 3 to 5 days. Subsequently, they were dried in a shaded area for future use. This processing method had been preserved to date as a traditional technique for ethnic minorities to consume *R. decorum* Franch. However, the nutritional value and potential toxicity risks of the processed edible dried corollas remained unclear. Hence, to develop the corollas of *R. decorum* Franch. as a valuable traditional ethnic flower food, further in-depth research and comprehensive food safety assessments are necessary.

**Figure 1:**
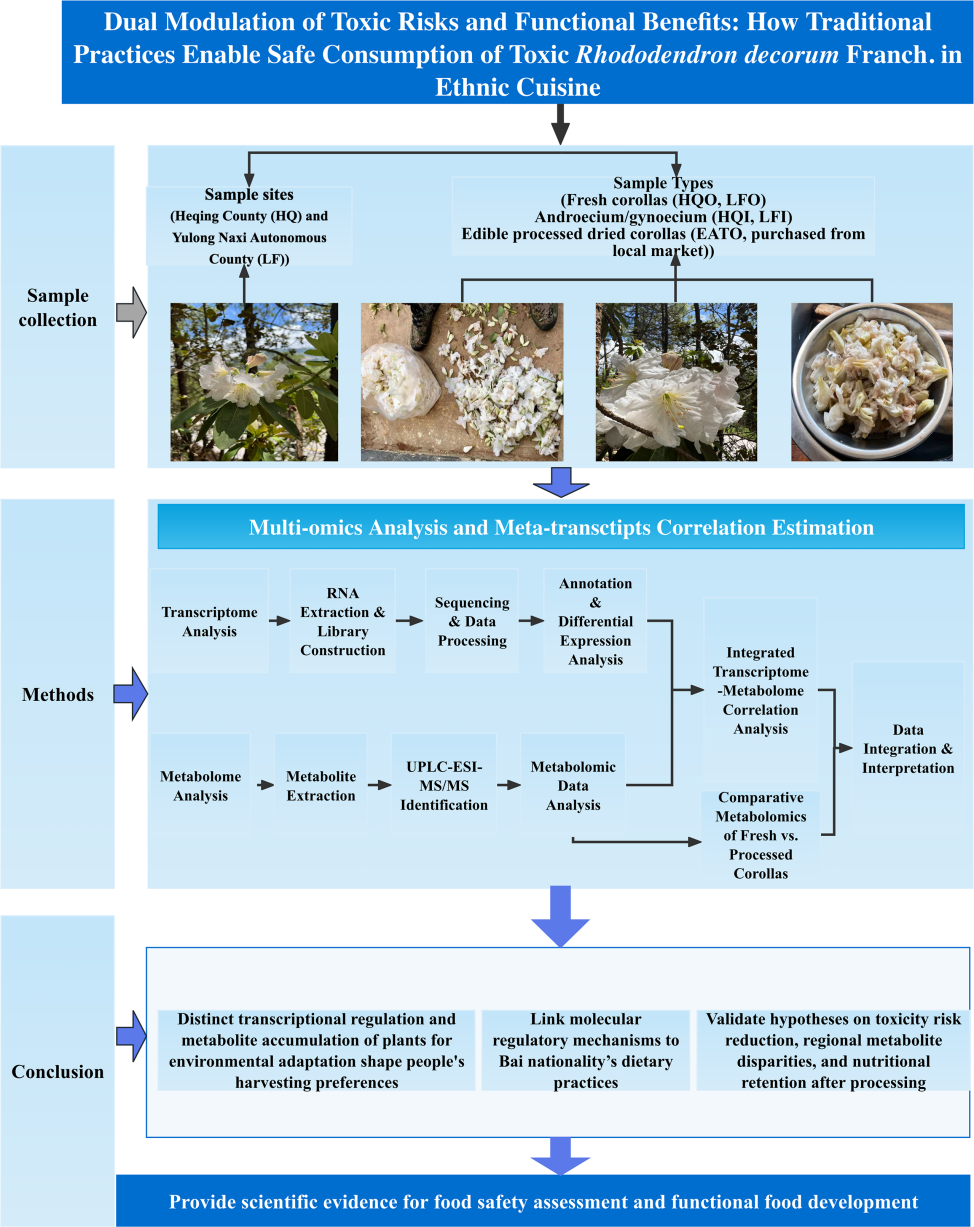
The workflow diagram of this study.

Panomics technologies, including transcriptomics and metabolomics, have been extensively employed in diverse aspects of plant research, including plant development, stress tolerance, and the accumulation of specialized metabolites (Mishra et al., 2024; Wang et al., 2025). To conduct a comprehensive assessment of the nutritional values and risks associated with the corollas of *R. decorum* Franch., a traditional flower food consumed by the Bai ethnic minority, an integrated approach that combined transcriptomics–metabolomics analysis and comparative metabolomics methods was employed. Based on the above background, this study focuses on three core scientific questions: (1) What is the molecular basis for the Bai people’s traditional practice of only consuming the corollas of *R. decorum* Franch. and removing the androecium/gynoecium? (2) Are there significant differences in the edible-related traits of *R. decorum* Franch. corollas from different main producing areas? (3) How does the traditional pre-consumption processing affect the nutritional value and edible safety of *R. decorum* Franch. corollas? By answering these questions, we aimed to provide a comprehensive scientific explanation for the traditional dietary custom of Bai nationality towards *R. decorum* Franch. corollas (**Figure 1**).

## 2 Materials and methods

### 2.1 Sample collection

In May 2024, healthy flowers of *R. decorum* Franch. were collected from Heqing County (HQ) (100.075720°E, 26.485620°N, altitude 3,038 m), Dali Bai Autonomous Prefecture and Yulong Naxi Autonomous County (LF) (100.180355°E, 27.001036°N, Altitude 3220 m), Lijiang City, Yunnan Province, China. To minimize the influence of an excessively close genetic background among individuals within the same population at the sample sites (Laurentin, 2009), three distinct populations separated by a distance of over 50 meter were selected at each sample site. Within each population, five healthy individuals of *R. decorum* Franch. were randomly chosen for sampling. For each individual, three to five intact flowers were collected and then separated into the corolla samples (named HQO and LFO, respectively) and the androecium/gynoecium samples (named HQI and LFI, respectively). After sample collection, the same floral organ samples from the same population were pooled together and then divided into three groups based on distinct population. The edible-treated dry corolla of *R. decorum* Franch. (referred to as EATO) was purchased from the market. All samples were subsequently flash-frozen in liquid nitrogen for long-term storage at −80°C until used. The botanical identification of *R. decorum* Franch. was performed in accordance with the morphological description in Flora of China, and the voucher specimens were deposited in the Lijiang Alpine Botanic Herbarium (LJABH) at Lijiang Normal University (Specimen Nos. LJABH-20240500101, LJABH-20240500102, LJABH-20240500201, LJABH-20240500202, LJABH-20240500301, LJABH-20240500302, LJABH-20240500401, LJABH-20240500402, LJABH-20240500501, LJABH-20240500502, LJABH-20240500601, and LJABH-20240500602). The dried corollas of commercially available *R. decorum* Franch. were initially verified through the identification of flower characteristics and follow-up visits to the original collection sites. All specimens and samples were identified by Weiwei Liu from the Lijiang Forest Biodiversity National Observation and Research Station, Chinese Academy of Sciences.

### 2.2 RNA extraction, cDNA library construction, and transcriptome sequencing

Approximately 5 g of each sample (including corollas and androecium/gynoecium samples) was used for extracting the total RNA by employing the Qiagen RNeasy Plant Mini Kit (Qiagen Company, Hilden, Germany) in accordance with the manufacturer’s instructions. The quantity and quality of extracted total RNA were evaluated by the NanoDrop 2000 spectrophotometer (Thermo Fisher Scientific Co. Ltd., Waltham, MA, USA) and Agilent 2100 Bioanalyzer (Agilent Technologies, Santa Clara, CA, USA). RNA samples with a minimum RNA Integrity Number (RIN) value equal to or greater than 8.0 and with respective absorbance ratios ranging from 1.8 to 2.0 at wavelengths of both *A*_260_/*A*_280_ nm were selected for subsequent cDNA library construction and sequencing (Shi et al., 2024).

Purified total RNA (1 µg) from each sample was used to construct the cDNA libraries according to the instruction of the NEBNext^®^Ultra™ RNA Library Prep Kit (NEB, Ipswich, MA, USA). Initially, mRNA was purified from the total RNA and reverse-transcribed into first-strand cDNA using random hexamer primers and M-MuLV Reverse Transcriptase in NEBNext First Strand Synthesis Reaction Buffer (5×). Subsequently, second-strand cDNA synthesis was synthesized with DNA Polymerase I and RNase H enzymes. When the synthesized cDNA fragments reached an approximate length of 240 bp, they were further purified using the AMPure XP system (Beckman Coulter, Beverly, MA, USA). The blunt-ended cDNA fragments were then ligated with adapters containing a “T” nucleotide overhang at their 3′ ends to generate paired-end libraries for sequencing. PCR amplification and enrichment of the cDNA libraries were performed by using adaptor region-based primers. The quality of the sequencing libraries was evaluated on an Agilent Bioanalyzer 2100 system (Agilent Technologies, Santa Clara, CA, USA). Finally, the constructed cDNA libraries were sequenced by an Illumina HiSeqXTM10 instrument (Illumina Inc., San Diego, CA, USA) at Biomarker Technologies Co., Ltd. (Beijing, China).

### 2.3 Transcriptome assembly, annotation, and differential expression analysis

The clean reads were obtained by adapter trimming and the removal of the low-quality reads from the raw transcriptome data, and then they were *de novo* assembled by using Trinity software (version 2.14.0) to generate the transcripts (Camacho et al., 2023; Grabherr et al., 2011). All transcripts were further analyzed to eliminate redundancies and obtain non-redundant unigenes. These non-redundant unigenes were subjected to blast analysis against major public databases, including the NCBI non-redundant protein sequence database (NR database) (https://www.ncbi.nlm.nih.gov/protein/) (accessed on 1 July 2024) (Bairoch and Apweiler, 2000), Swiss Prot database (https://www.uniprot.org/uniprot/) (accessed on 1 July 2024) (Tatusov et al., 2003), Clusters of Orthologous Groups (COG) (https://www.ncbi.nlm.nih.gov/research/cog) (accessed on 1 July 2024), Clusters of Protein homology (KOG) (https://ftp.ncbi.nlm.nih.gov/pub/COG/KOG/) (accessed on 1 July 2024) (Ashburner et al., 2000), eggNOG4 (http://eggnog6.embl.de) (accessed on 1 July 2024) (Huerta-Cepas et al., 2016), and the Pfam database (https://pfam.xfam.org) (accessed on 1 July 2024) (Bateman et al., 2002), with a cutoff value below 10^−5^ for homologous alignment. Gene Ontology was conducted to annotate the identified unigenes by using Blast2GO software (version 2.5) with default parameters (Kanehisa et al., 2004). Furthermore, TransDecoder software (v5.0.0) (https://transdecoder.github.io/) (accessed on 1 July 2024) was employed to predict the coding region sequences (CDSs) of unigenes with the translated amino acid sequences against the Pfam database.

Also, to verify the function of the identified unigenes, they were subjected to blast in the Kyoto Encyclopedia of Genes and Genomes (KEGG) database with a threshold of 1 × 10^−5^ (Xie et al., 2011). Additionally, the KEGG orthology (KO) analysis was performed to assign the identified unigenes into biosynthesis pathways by employing the KOBAS software (version 3.0.3) with default parameters. The fragments per kilobase per million fragments mapped (FPKM) values were estimated by the Expectation-Maximization (RSEM) software (version 1.3.3) to present the gene expression levels of unigenes from RNA sequencing (RNA-seq) data (Li and Dewey, 2011). Differential expression analysis was performed to identify the differentially expressed genes (DEGs) within different groups based on the negative binomial distribution by using DESeq2 software (version 1.30.1). The resulting *p*-values were adjusted by means of Benjamini and Hochberg’s approach to control the false discovery rate, and the significance threshold was an adjusted *p*-value less than 0.05 (Love et al., 2014).

### 2.4 Metabolite extraction identified by UPLC-ESI-MS/MS

The metabolomics analysis was carried out in cooperation with Biomarker Technologies Co., Ltd. (Beijing, China), and it encompassed a series of specific procedures. At first, 100 mg of crushed and lyophilized powder was immersed in 1,200 µL of a 70% methanol aqueous solution and extracted for 180 min at −20°C. During the extraction procedure, the mixtures were vortexed once every 30 min, each time lasting for 30 s, totaling six times. After centrifugation at 12,000 rpm for 3 min, the supernatant was filtered through a 0.22-µm membrane. The quantification of the metabolites in the *R. decorum* Franch. samples was carried out using ultra-performance liquid chromatography–electrospray ionization tandem mass spectrometry (UPLC-ESI-MS/MS) technology. Established methods were employed, and an LC-ESI-MS/MS system (UPLC, gasket-packed UFLC CBM30A, Shimadzu; MS, 6500 QTRAP, Applied Biosystems, Shanghai, China) was utilized. All sample extracts were then analyzed (Fraga et al., 2010; Gong et al., 2023). Mobile phases A and B were respectively composed of ultrapure water containing 0.1% formic acid and acetonitrile containing 0.1% formic acid. According to the gradient program, phase B changed from 5% to 95% within 9 min and then was promptly reduced back to 5% and adjusted for an additional 14 min after being maintained at that level for 1 min. The column temperature was kept at 40°C, while the flow rate of the mobile phase remained constant at 0.35 mL·min^−1^, and each injection volume was 4 µL.

The ESI process conditions are presented as follows: The temperature was fixed at 550°C; the ion spray voltage (IS) was 5,500 V in the positive ion mode and −4,500 V in the negative ion mode; gas I (GSI), gas II (GSII), and the curtain gas (CUR) were respectively regulated to 50, 60, and 25 psi; high-collision activated dissociation (CAD) was employed. QQQ scans were planned as multiple reaction monitoring (MRM) experiments with medium collision gas (nitrogen). The declustering potential (DP) and collision energy (CE) for individual MRM transitions were optimized along with additional adjustments. A particular set of MRM transitions was carried out for each period in accordance with the eluted metabolites within that time frame (Duan et al., 2018).

### 2.5 Comparative metabolome data analysis

After normalizing the original peak area information of metabolites by the total peak area, a subsequent analysis was carried out. Principal component analysis (PCA) and Spearman correlation analysis were employed to assess the repeatability of the samples within groups and the quality control samples. Regarding the identified compounds, their classification and pathway information were obtained from the KEGG database, the Human Metabolite Database (HMDB), and the LIPID MAPS Database, respectively (Conroy et al., 2024; Wishart et al., 2018; Xie et al., 2011). Based on the grouping information, the difference multiples were calculated and compared, and a *t*-test was utilized to calculate the *p*-value of differential significance for each compound. The R language package ropls was adopted to conduct orthogonal signal correction and partial least squares-discriminant analysis (OPLS-DA) modeling, and 200 permutation tests were carried out to validate the reliability of the model (Zhou et al., 2025). The variable importance in projection (VIP) values of the model were computed through multiple cross-validation. The approach of integrating the difference multiples, the *p*-values, and the VIP values of the OPLS-DA model was utilized to sift the differential metabolites. The screening thresholds were set by the parameters of |log2foldchange| (FC) > 1, *p*-value < 0.05 and VIP value > 1. The differential metabolites with significant KEGG pathway enrichment were calculated by means of the hypergeometric distribution test (Yu et al., 2012).

### 2.6 Integrative transcriptome–metabolome correlation analysis

To investigate the interaction of transcriptional genes and metabolites, two-way orthogonal partial least squares (O2PLS) modeling was employed to assess the overall impact between distinct data sets. This approach will directly reflect the weights of various variables within the model (Chao et al., 2023). Additionally, joint principal component analysis was carried out to assess the dispersion degree of samples. In the correlation analysis, Pearson correlation coefficients were computed for all genes and metabolites within each differential group. Subsequently, screening and visualization were conducted based on an absolute correlation coefficient (CC) greater than 0.80 and a correlation *p*-value less than 0.05 (Xu et al., 2023). Hierarchical cluster analysis was performed on DEGs and differentially accumulated metabolites (DAMs) based on their expression levels, offering a comprehensive manifestation of the differences in their expression patterns. DEGs and DAMs were further categorized using K-means clustering for each group independently to determine the correlation trends between them. Through KEGG enrichment analysis, the pathways commonly enriched by significantly DEGs/DAMs were identified (Han et al., 2023). Redundancy analysis (RDA) was subsequently employed to further analyze the typing trends of DEGs and DAMs in these significantly enriched pathways. Finally, inter-group network analysis was conducted using the Spearman correlation coefficient to construct a correlation matrix between DEGs and DAMs (Jiang et al., 2021; Pathania et al., 2016). Node values, line values, and co-occurrence patterns were computed and visualized to illuminate the profound relationships between DEGs and DAMs.

## 3 Results

### 3.1 Different distribution patterns of DEGs and DAMs in different floral organs

#### 3.1.1 DEGs/DAMs distribution pattern in different groups

To explore the correlation between transcriptional genes and metabolites, the O2PLS and PCA were employed to assess the relevance of transcripts and metabolites within and between the LFI/LFO group and the HQI/HQO group. Results indicated that the DAMs in the LFI/LFO group mainly fell into quadrants 1 and 4, where the DEGs were primarily situated in quadrants 2 and 3 (**Supplementary Figure 1A**). In the LFI/LFO group, the regulation pattern of the accumulation of these DAMs might exhibit negative feedback from DEGs. However, the distribution pattern of DAMs and DEGs in the HQI/HQO group was distinct from that in the LFI/LFO group. DAMs and DEGs were distributed throughout all quadrants, and their regulatory interactions showed a high degree of diversity (**Supplementary Figure 1B**). Also, the PCA demonstrated that the DEGs and DAMs were strongly aggregated in each group (LFI/LFO; HQI/HQO), suggesting a high level of sample repeatability (**Supplementary Figures 1C, D**).

#### 3.1.2 Comparative KEGG enrichment analysis of DEGs/DAMs in different groups

Through functional estimation of the DEGs and DAMs within different groups, the comparative KEGG enrichment pathways analysis was conducted to predict the potential functions of identified DEGs and DAMs. Results showed that 3,463 DEGs and 165 DAMs in the LFI/LFO group were categorized into 148 pathways, where they shared 83 pathways, and DEGs had 53 private pathways and DAMs had 12 pathways (**Supplementary Figure 2A**). The predominant pathway was the plant hormone signal transduction pathway, which had a relative low content of DAMs, followed by the carbon metabolism and biosynthesis of amino acids, in which the biosynthesis of amino acids pathway had the highest DAMs (**Supplementary Figure 2B**). In the HQI/HQO group, 3,229 DEGs and 145 DAMs were grouped into 144 pathways, where they shared 74 common pathways. Moreover, the DEGs had private 58 pathways and DAMs had 12 private pathways (**Supplementary Figure 2C**). The results of KEGG enrichment analysis towards DEGs and DAMs in the HQI/HQO group exhibited the same distributed patterns with that in the LFI/LFO group (**Supplementary Figure 2D**).

**Figure 2:**
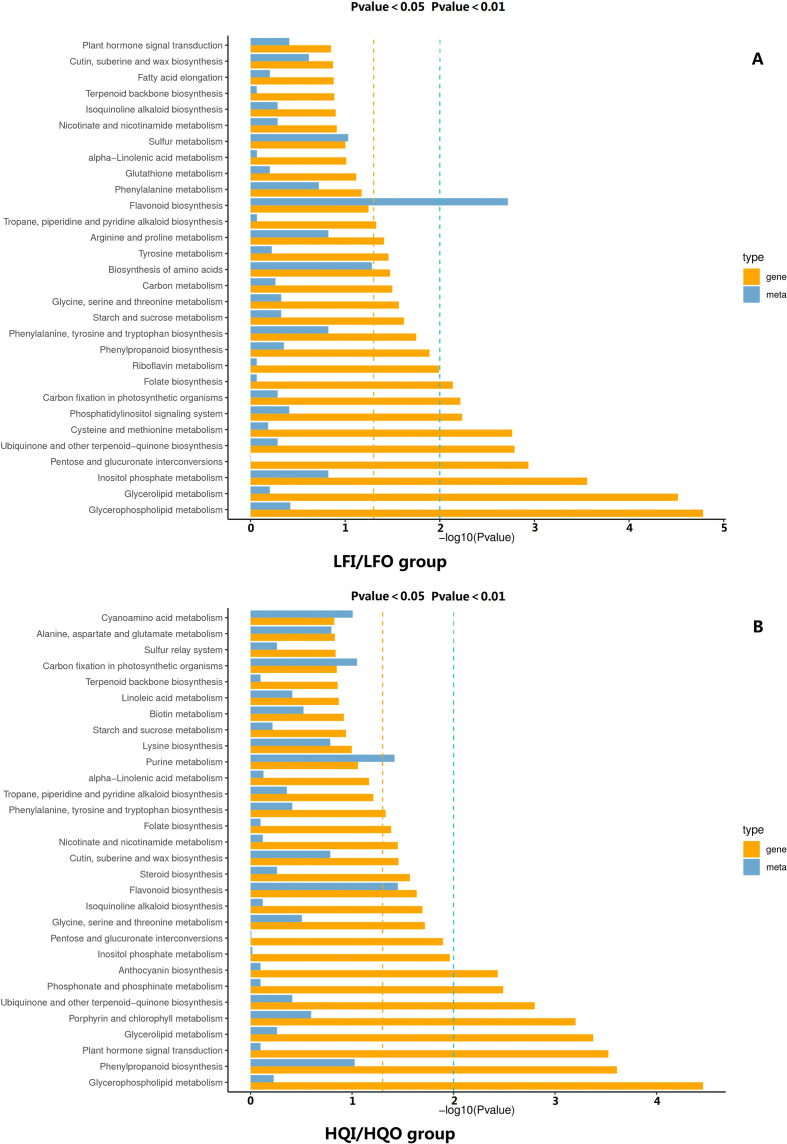
The top 30 enriched pathways of KEGG enrichment analysis towards DEGs/DAMs association from two comparative groups. **(A)** DEGs/DAMs enriched in the LFI/LFO group. **(B)** DEGs/DAMs enriched in the HQI/HQO group. Yellow columns represent DEGs in different enriched KEGG pathways, and blue columns are for the DAMs. The vertical axis shows pathway names, and the horizontal axis shows the significance of pathway enrichment (log-transformed FDR values). The height of columns indicates the significant enrichment.

Furthermore, to deeply understand the functions of DEGs/DAMs association within KEGG pathways, the enrichment analysis was performed within two comparative groups of corollas and androecium/gynoecium from two regions. Results showed that the DEGs/DAMs in the LFI/LFO group significantly associated with enrichment in the flavonoid biosynthesis pathway (*p* < 0.1), the biosynthesis of amino acids pathway (*p* < 0.1), and the sulfur metabolism pathway (*p* < 0.1) (**Figure 2A**; **Supplementary Table 1**). In the HQI/HQO group, DEGs/DAMs exhibited significantly associated enrichment in the flavonoid biosynthesis pathway (*p* < 0.05), the phenylpropanoid biosynthesis pathway (*p* < 0.1), and the purine metabolism pathway (*p* < 0.1) (**Figure 2B**; **Supplementary Table 2**). Although most of the top 30 pathways showed significant enrichment of DEGs or DAMs across various floral organs in different regions, the associations between DEGs and DAMs were not observed in these pathways.

#### 3.1.3 Correlation analysis of DEGs/DAMs within different organs

To estimate the correlations between all identified DEGs and DAMs within different organs, the correlation coefficient and the *p*-value were calculated. Results indicated that DEGs/DAMs were apparently divided into two groups within the LF group or the HQ group, some of which showed the consistency of DEGs’ expression and metabolite accumulation trends, whereas some of them showed an opposite correlation. Additionally, the expression level of a proportion of DEGs was stable and their associated metabolites showed either upregulation or downregulation (**Supplementary Figures 3A, B**). Hierarchical cluster analysis of DEGs/DAMs according to their expression and accumulation levels revealed that the gene expression patterns within different floral organs were significantly different from the metabolite’s accumulation (**Supplementary Figures 3C, D**). Moreover, K-means cluster analysis also indicated that the transcriptional–metabolic regulatory patterns of the androecium/gynoecium and corolla were opposite, reflecting distinct biological functions of different organs in the LFI/LFO group (**Supplementary Figures 3E–H**). In contrast, the transcriptional–metabolic regulatory patterns of the androecium/gynoecium and corolla in the HQI/HQO group were found to be largely congruent (**Supplementary Figures 3I–L**), indicating coordinated biological functions across different organs in the HQI/HQO group.

**Figure 3:**
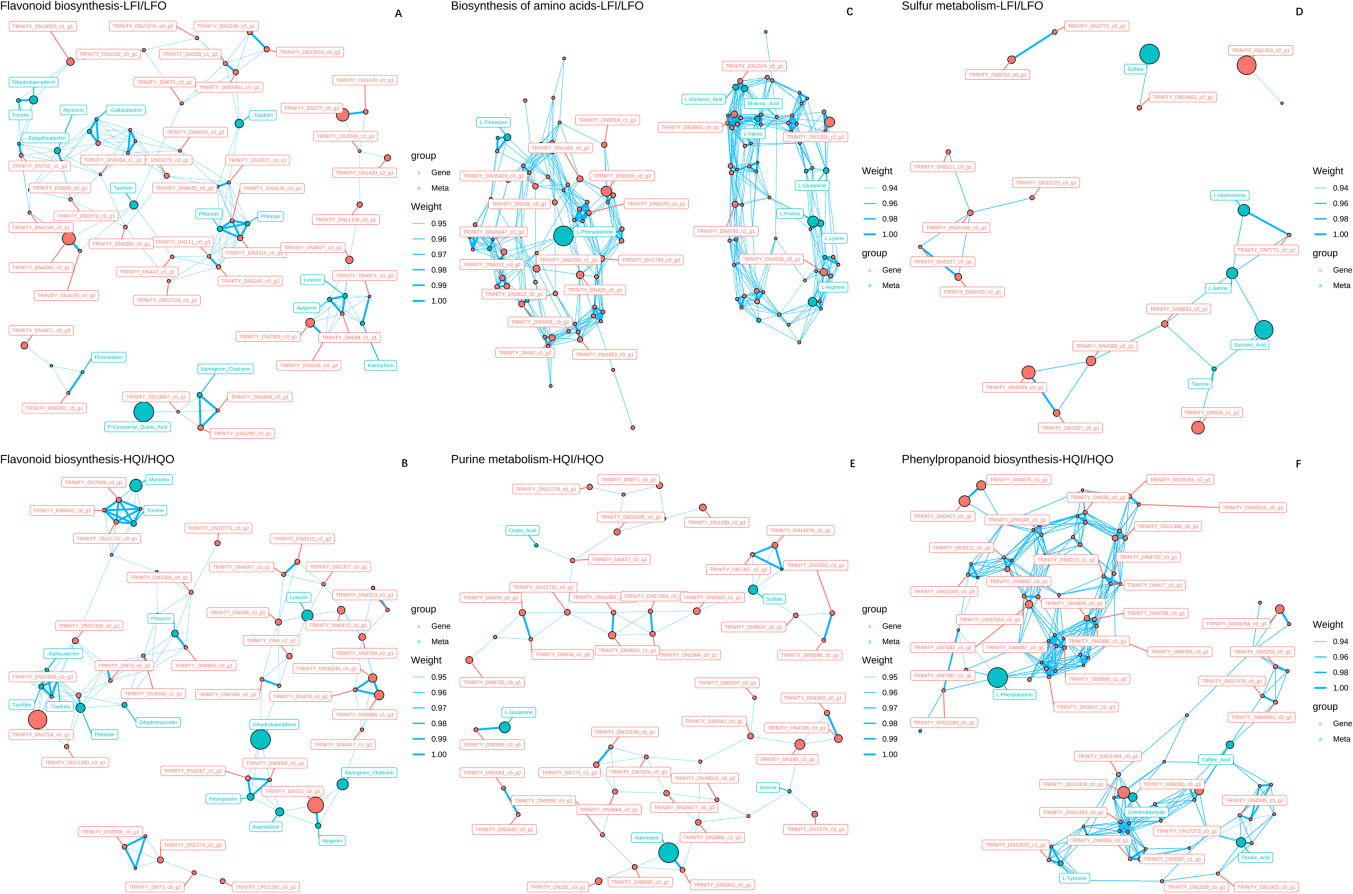
Correlation regulatory network analysis of associated DEGs/DAMs within different metabolic pathways. **(A)** The correlation network of DEGs/DAMs in the flavonoid biosynthesis pathway for the LFI/LFO group. **(B)** The correlation network of DEGs/DAMs in the flavonoid biosynthesis pathway for the HQI/HQO group. **(C)** The correlation network of DEGs/DAMs in the biosynthesis of amino acid pathway for the LFI/LFO group. **(D)** The correlation network of DEGs/DAMs in the sulfur metabolism pathway for the LFI/LFO group. **(E)** The correlation network of DEGs/DAMs in the purine metabolism pathway for the HQI/HQO group. **(F)** The correlation network of DEGs/DAMs in the phenylpropanoid biosynthesis pathway for the HQI/HQO group. The correlation coefficient matrix for DEGs and DAMs determines the node values and edge weights in the network, which are then visualized. Red nodes represent genes, green nodes represent metabolites, and node size reflects expression levels. Blue edge thickness indicates correlation strength, with thicker lines showing stronger correlations.

Moreover, to assess the correlation of DEGs/DAMs enrichment in KEGG pathways, pathways that concurrently met the criteria of both DEG and DAM *p*-values being less than 0.1 and an enrichment factor greater than 1 were chosen for RDA, which enabled a comprehensive assessment of the profound correlation between DEGs and DAMs across various groups. Results showed that three pathways were successful in meeting the thresholds of both *p*-values and enriched factors in the LFI/LFO group, namely, flavonoid biosynthesis, biosynthesis of amino acids, and sulfur metabolism (**Supplementary Table 1**), where flavonoid biosynthesis, purine metabolism, and phenylpropanoid biosynthesis were found in the HQI/HQO group (**Supplementary Table 2**). Then, the inter-group correlation network analysis was performed towards the identified DEGs and DAMs in the LFI/LFO group and the HQI/HQO group within the aforementioned pathways (**Figure 3**). Results of the RDA revealed that all first RDA components account for more than 95%, indicating that the model successfully elucidates the correlation between DEGs and DAMs within various pathways. Notably, the DEGs/DAMs in the LFI/LFO group were shown to associate with both the androecium/gynoecium and corolla within the flavonoid biosynthesis pathway, but there was a lack of association in the corollas of the HQI/HQO group (**Supplementary Figures 4A, B**). In the biosynthesis of amino acid pathway and sulfur metabolism pathway, the DEGs/DAMs showed association in all organs of the LFI/LFO group (**Supplementary Figures 4C, D**), where the purine metabolism pathway and the phenylpropanoid biosynthesis pathway showed the same trends in all organs of the HQI/HQO group (**Supplementary Figures 4E, F**).

In the LFI/LFO group, four associated subnetworks were identified in the flavonoid biosynthesis pathway. A total of 15 DAMs showed significant correlation with 50 DEGs. Among them, p-coumaroyl quinic acid was extremely significant to gene TRINITY_DN13667_c0_g1, where dihydromyricetin exhibited a close relationship to tricetin and (−)-epigallocatechin (**Figure 2A**; **Supplementary Table 3**). One single regulatory network led by the chemicals pinocembrin and chrysin was specifically observed in the corollas from the LF region and was absent in the HQ region (**Figure 3A**; **Supplementary Table 3**). Also, metabolites (+)-taxifolin and taxifolin played a pivotal role in the subnetwork in terms of their correlation with other DAMs and DEGs. In the HQI/HQO group, metabolites dihydrokaempferol, luteolin, myricetin, and naringenin chalcone showed a significant correlation with DEGs and other DAMs, where gene TRINITY_DN1724_c0_g1 and TRINITY_DN721_c0_g1 were found to be significantly correlated to a series of DAMs (**Figure 3B**; **Supplementary Table 3**). Within the amino acid biosynthesis pathway, two complex regulatory networks were found in the LFI/LFO group. One regulatory network led by L-phenylalanine and L-threonine in the corollas, and another regulatory network led by L-proline, L-arginine, and L-lysine were found in the androecium/gynoecium (**Figure 3C**; **Supplementary Table 3**). Unlike the HQ region, the corollas in the LFI/LFO group possessed five fragmented regulatory networks in the sulfur metabolism pathway, which is composed of a primary network driven by succinic acid (**Figure 3D**; **Supplementary Table 3**). In the HQI/HQO group, they showed very contrasting regulatory network construction except for the flavonoid biosynthesis pathway. Four regulatory networks were identified in the purine metabolism pathway, led by adenosine, sulfate, L-glutamine, and L-glutamine **(Figure 3E**; **Supplementary Table 3**). Moreover, two complex regulatory networks were also found in the phenylpropanoid biosynthesis pathway. One network was dominated by L-phenylalanine and eugenol in the corollas of the HQI/HQO group, and the other network was regulated by ferulic acid, coniferaldehyde, and caffeic acid in the androecium/gynoecium (**Figure 3F**; **Supplementary Table 3**).

### 3.2 Multi-omics analysis of edible corollas across different regions

**Figure 4:**
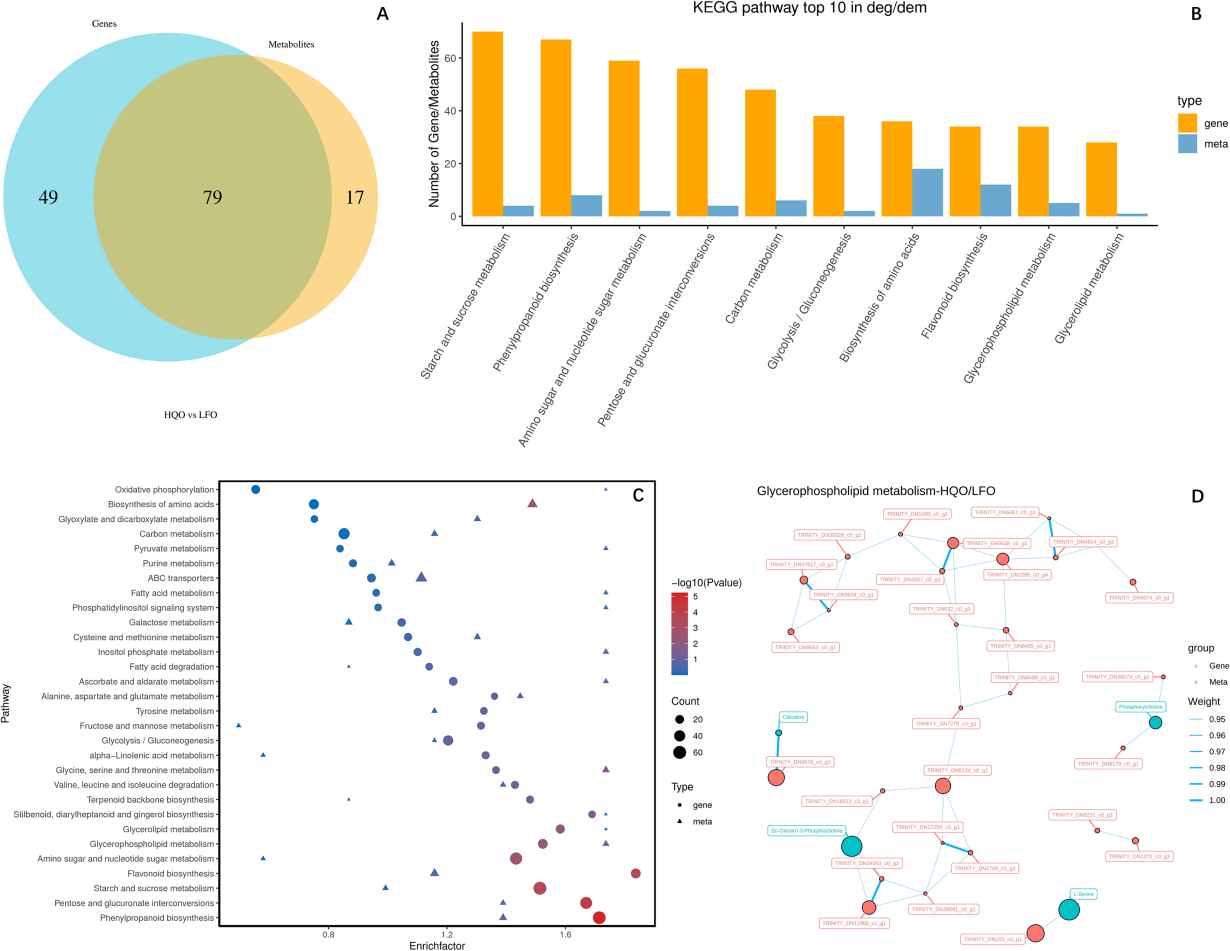
Multi-omics comparative analysis of edible corollas across different regions. **(A)** Venn analysis of pathways involved DEGs and DAMs in the HQO/LFO group. **(B)** The top 10 KEGG enrichment pathways of DEGs/DAMs in the HQO/LFO group; the yellow bar represents the DEGs and the blue bar denotes the DAMs. **(C)** The top 30 KEGG enrichment pathways of DEGs/DAMs in the HQO/LFO group. The *x*-axis shows the enrichment factor of pathways across different omics, and the *y*-axis shows the KEGG pathway names. The red-to-blue gradient indicates decreasing enrichment significance. Circles represent the DEGs, triangles represent the DAMs, and larger bubbles indicate more DEGs/DAMs. **(D)** Correlation regulatory network analysis of DEGs/DAMs within glycerophospholipid metabolism pathways. Red nodes represent DEGs, green nodes represent DAMs, and node size reflects expression levels. Blue edge thickness indicates correlation strength, with thicker lines showing stronger correlations.

To understand the preference of the Bai ethnic group towards corollas of *R. decorum* Franch., multi-omics analysis was employed to compare the edible corollas of *R. decorum* Franch. distributed in the Lijiang and Heqing regions. Results revealed that associated DEGs/DAMs were grouped into 145 pathways in HQO vs. LFO, where DAMs possessed private 17 pathways and DEGs had their own 49 pathways, and they shared 79 common pathways (**Figure 4A**). Moreover, the associated DEGs/DAMs were further classified into functional pathways to comprehensively understand the potential functions of DEGs and DAMs. Most of the associated DEGs and DAMs were classified into the top 10 pathways, namely, starch and sucrose metabolism, phenylpropanoid biosynthesis, amino sugar and nucleotide sugar metabolism, pentose and glucuronate interconversions, carbon metabolism, glycolysis/gluconeogenesis, biosynthesis of amino acids, flavonoid biosynthesis, glycerophospholipid metabolism, and glycerolipid metabolism (**Figure 4B**). Through co-enrichment analysis of DEGs/DAMs in the corollas from different regions, it was found that only the glycerophospholipid metabolism pathway exhibited significance in the HQO/LFO group. Also, there were differential enrichments of DEGs in the phenylpropanoid biosynthesis, pentose and glucuronate interconversions, starch and sucrose metabolism, and flavonoid biosynthesis pathways, and of DAMs in the biosynthesis of amino acids and glycine, serine, and threonine metabolism pathways; however, co-enrichment analysis showed that they were not significantly correlated (**Figure 4C**). Within the glycerophospholipid metabolism pathway, five regulatory networks were found to have significant correlations. A complex network led by the metabolite Sn-glycero-3-phosphocholine and numerous genes was constructed to show the association in the corollas of the compared HQO/LFO group. Also, citicoline, L-serine, and phosphorylcholine were found to construct a very simple regulatory network with one or two genes (**Figure 4D**; **Supplementary Table 3**).

### 3.3 Comparative metabolomic analysis of edible processed dried corollas and fresh corollas of *R. decorum* Franch. from different regions

To gain a deep understanding of the transformation of fresh corollas into the edible corollas consumed by the Bai people, a comparative metabolomic analysis of edible processed dried and fresh corollas from two regions was conducted to verify the metabolite changes after processing. After the fresh corollas were processed into edible dried corollas, it was observed that some of the DAMs exhibited mixture accumulation in different comparison groups. Within the EATO/HQO and EATO/LFO groups, the corollas shared the common DAMs that exhibited up - level accumulation, such as ferric ammonium citrate, methyl tridecanoate, linalool, and (3R, 5S, E) - 1,7 - Diphenylhept - 1 - ene - 3,5 - diol. In contrast, other DAMs like tyramine, (−)-epigallocatechin, (+)-gallocatechin, tricetin, sequoyitol, and (−)-catechin gallate showed down - level accumulation (**Supplementary Figure 5**). In addition to the regulated accumulation of the aforementioned DAMs within the two compared groups, certain DAMs, such as heptanal oxime, ferulic acid, vanillic acid, zeranol, and padmatin, were also observed to exhibit upregulated accumulation in the EATO/HQO group (**Supplementary Figure 5A**). Meanwhile, DAMs such as taxifolin, hydroxyhexanoic acid, and 4-ethyloctanoic acid showed upregulated accumulation in the EATO/LFO group (**Supplementary Figure 5B**). Additionally, the total identified DAMs within the EATO/HQO and EATO/LFO groups were also calculated. Results showed that 1,340 DAMs were identified in the EATO/HQO and EATO/LFO group. Among them, 277 DAMs were upregulated and 356 DAMs were downregulated in the EATO/HQO group. In the EATO/LFO group, 311 DAMs were upregulated and 303 DAMs were downregulated (**Supplementary Figures 5C, D**). These results indicated that nearly half of the identified DAMs exhibited significant changes after processing the fresh corollas into edible dried corollas.

**Figure 5:**
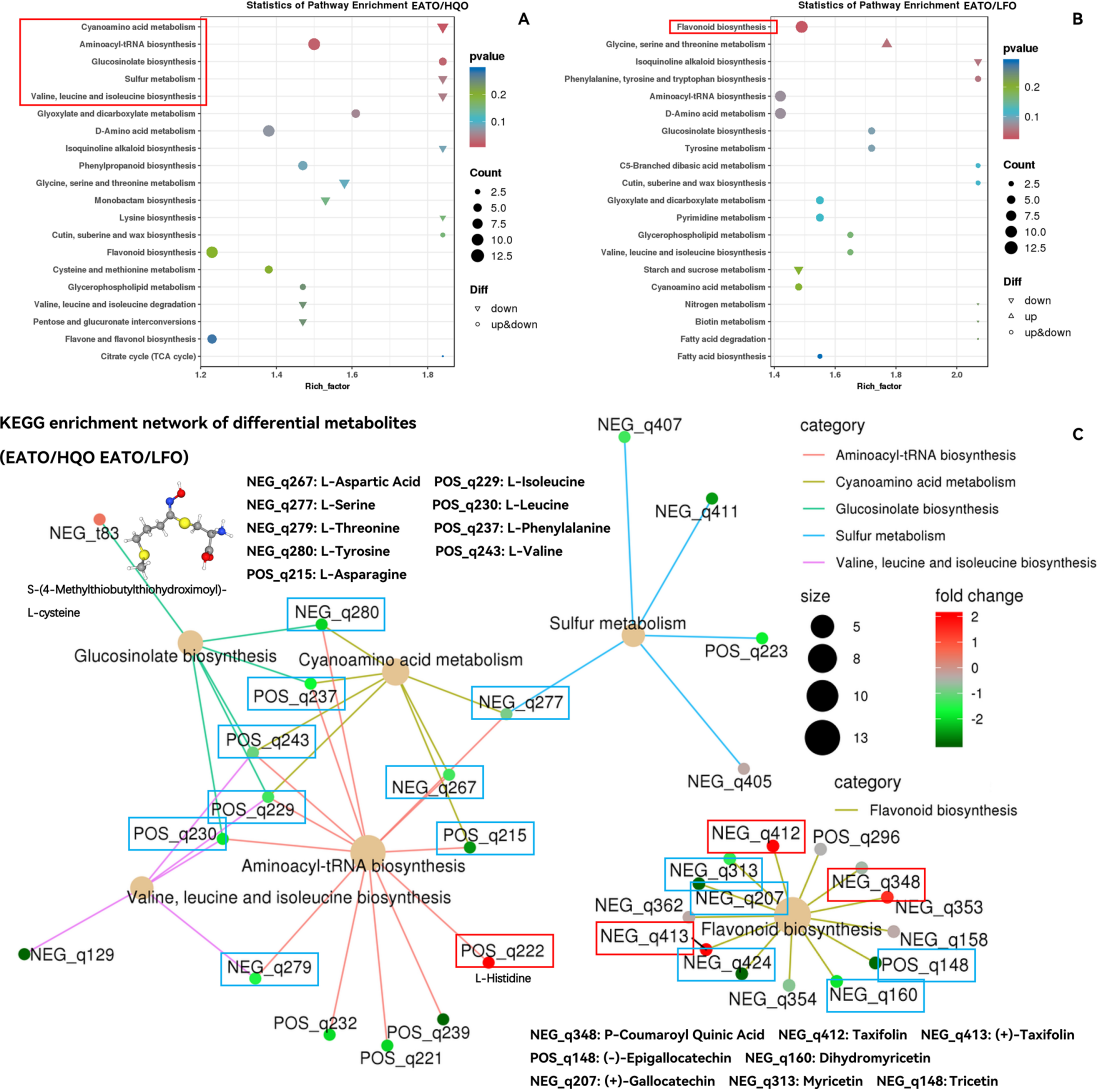
KEGG enrichment and network analysis of DAMs within the EATO/HQO and EATO/LFO group. **(A)** Top 20 KEGG enrichment pathways in the EATO/HQO group. **(B)** Top 20 KEGG enrichment pathways in the EATO/LFO group. The *x*-axis represents the rich factors; the *y*-axis indicates the top 20 pathways. Dot color intensity reflects the *p*-value, with significantly enriched pathways marked by a red box. Dot size indicates the number of DAMs in the pathway, while dot shape denotes regulated patterns: upregulated (triangle up), downregulated (triangle down), or mixed (circle). **(C)** KEGG enrichment network analysis of DAMs in the EATO/HQO and EATO/LFO group. Light-yellow nodes represent pathways, and the connected small nodes were DAMs annotated to these pathways. Node size indicates the number of annotated DAMs, and color depth reflects the log2(FC) value. Key DAMs were marked with colored boxes: red for upregulation and blue for downregulation.

After functional analysis of all identified DAMs within the two compared groups, KEGG enrichment analysis was performed to categorize them into different pathways. Results showed that five pathways were found to significantly enrich DAMs in the EATO/HQO group (*p* < 0.05), namely, cyanoamino acid metabolism; aminoacyl-tRNA biosynthesis; glucosinolate biosynthesis; valine, leucine and isoleucine biosynthesis; and sulfur metabolism (**Figure 5A**; **Supplementary Table 4**). However, only the flavonoid biosynthesis pathway significantly enriched the DAMs (*p* < 0.05) in the EATO/LFO group (**Figure 5B**; **Supplementary Table 4**). These findings suggested that the transformation patterns of DAMs showed significant differences between the fresh corollas derived from the HQ and LF regions and the edible dried corollas. To understand the in-depth relationship of the enriched KEGG pathways, a network analysis of DAMs involved in the significantly enriched pathways was conducted. Results showed that the DAMs were enriched in the aminoacyl-tRNA biosynthesis pathway within the EATO/HQO group, which acts as a central hub pathway to regulate the biosynthesis of various amino acids and consequently influence the production of a series of metabolites in downstream metabolic pathways (**Figure 5C**). Interestingly, only L-histidine was shown to be upregulated in edible dried corollas within the aminoacyl-tRNA biosynthesis pathway. Also, DAMs like p-coumaroyl quinic acid, taxifolin, and (+)-taxifolin were found to upregulate in edible dried corollas within the flavonoid biosynthesis pathway, whereas (−)-epigallocatechin, (+)-gallocatechin, myricetin, and tricetin were downregulated in edible dried corollas (**Figure 5C**).

## 4 Discussion

### 4.1 Evidence and potential mechanism for consuming corollas of *R. decorum* Franch

The corollas of *R. decorum* Franch., which are regarded as a traditional food by the Bai people, are usually cooked with meat as a vegetable supplement to enhance the flavor and nutrition of the dishes (Shi et al., 2021). Generally, the Bai people prefer to harvest the corollas and remove the androecium/gynoecium of *R. decorum* Franch. flowers and then process them through hot water treatment, which is regarded as a crucial step in ensuring the safe consumption of *R. decorum* Franch. corollas. To unveil the molecular basis of food customs regarding the consumption of the corollas from *R. decorum* Franch., a comparative KEGG enrichment analysis of associated DEGs/DAMs was carried out. Significantly, associated DEGs/DAMs were enriched in the flavonoid biosynthesis pathway in both Heqing and Lijiang regions. When comparing the shared key flavonoids in the androecium/gynoecium from Heqing and Lijiang regions, several flavonoids and their derivatives were identified, including myricetin, phloretin, gallocatechin, taxifolin, luteolin, apigenin, phlorizin, kaempferol, naringenin chalcone, tricetin, and dihydrokaempferol. Although the anti-cancer, anti-inflammatory, antioxidant, and detoxification properties of these flavonoids have been comprehensively verified (de Oliveira et al., 2021; Mishra et al., 2024; Sohel et al., 2024), the potential risks associated with flavonoid consumption must be seriously considered. For instance, the increases in cell surface roughness and the decreases in elasticity induced by myricetin intensified the toxic effects of copper exposure, leading to a higher cellular mortality rate (Sadžak et al., 2021). When utilized as a dietary supplement, gallocatechin may have potentially toxic effects on the liver (Oketch-Rabah et al., 2020). Kaempferol, a compound present in food supplements that exhibits protective effects, may also induce DNA damage and cell apoptosis (Niering et al., 2005). Furthermore, studies have confirmed that the aqueous extract of flavonoids from *Rhododendron*-related species exhibited significant dose-dependent genotoxicity *in vitro* (Gazim et al., 2022), which further indicated that the excessive accumulation of flavonoids in the androecium/gynoecium of *R. decorum* Franch. might bring potential genotoxic risks to consumers. They may interact with the human ether-a-go-go-related gene (hERG) channel, resulting in its blockage and potentially giving rise to a lethal ventricular tachyarrhythmia (Saponara et al., 2021). Therefore, in comparison with the consumption of corollas, the androecium/gynoecium of *R. decorum* Franch. exhibited a higher risk of organ damage and mortality.

Within the amino acid biosynthesis pathway, L-phenylalanine was significantly enriched in the corollas of the LFI/LFO group. This finding may be associated with floral fragrance production, suggesting that plants in high-altitude regions need to attract more pollinating insects (Zeng et al., 2019). In the androecium and gynoecium organs, the significant accumulation of L-glutamic acid and L-arginine indicated the cold-resistance mechanism of plants surviving at higher altitudes (Yang et al., 2024). Additionally, various amino acids, such as L-valine, L-proline, and L-glutamine, had been proven to play a key role in the reproductive processes of plants (Wei et al., 2023). In the LFI/LFO group, L-serine and succinic acid were found to be significantly enriched in the sulfur metabolism pathway within the androecium/gynoecium. These compounds partake in the synthesis of autonomous auxin in pollen grains via the phosphorylation of serine biosynthesis and influence the development and maturation of plant embryos and pollen (Amanda et al., 2022; Casatejada-Anchel et al., 2021). As a constituent of nectar, taurine enhanced the specific memory retention of pollinating insects, thus enhancing the adaptability of *R. decorum* Franch. to the decreasing population of pollinators in high-altitude environments (Carlesso et al., 2021). Although there was no direct evidence to substantiate the function of sulfates, sulfates exhibit a significant enrichment in corollas. It is hypothesized that the sulfates may serve as a potential catalyst to facilitate the formation of floral fragrance (Zou et al., 2021). The enrichment patterns of DAMs demonstrated by distinct flower organs within the amino acid biosynthesis pathway and sulfur metabolism pathway reflect the differentiation of their floral organ functional adaptation.

In the HQI/HQO group, adenosine and inosine were enriched in the purine metabolism pathway, which contributed to energy production and exerted protective effects during the synthesis of plant nucleic acids (Choi, 2024; Straube et al., 2023). Oxalic acid was also significantly enriched in the purine metabolism pathway. Evidence indicated that the oxalic acid treatment would strongly enhance the plant cold resistance by modulating fruit energy metabolism (Jin et al., 2014; Li et al., 2014). Therefore, it was hypothesized that this mechanism might serve as a protective strategy for the reproductive organs of *R. decorum* Franch. in subalpine environments. In the phenylpropanoid biosynthesis pathway, L-phenylalanine was significantly enriched and served as a crucial core precursor for synthesizing various aromatic compounds such as eugenol and isochavicol (Zeng et al., 2019). These compounds not only attracted flower-visiting insects but also exhibited antioxidant properties (Parrot et al., 2021; Yan et al., 2018). Moreover, a significant enrichment of p-coumaryl alcohol suppressed the lignification process, thereby improving the edible quality of corollas (Zou et al., 2019).

Through the correlation network analysis of DAMs in distinct flower organs (corolla and androecium/gynoecium), it was discovered that several metabolic pathways were enriched with a large number of DAMs. These DAMs demonstrated significantly different patterns in various organs. These differential regulatory patterns not only were evident in the accumulation of toxic substances but also reflected the disparities in aroma formation and palatability. These findings may contribute to a more profound understanding of the practice of removing the androecium/gynoecium and selecting the corollas for consumption of *R. decorum* Franch.

### 4.2 Variations of the edible corollas of *R. decorum* Franch. across different regions

Although the flowers of *R. decorum* Franch. in the Heqing area demonstrated a higher accumulation of secondary metabolites compared to those in the Lijiang area (Liu et al., 2024a), when combined with the DEGs for comprehensive analysis, the DEGs/DAMs in the HQO/LFO group were only significantly enriched in the glycerophospholipid metabolism pathway. After conducting a correlation network analysis of associated DEGs/DAMs, it was discovered that L-serine, which served as a synthetic precursor of choline compounds, participated in the biosynthesis of phosphorylcholine, citicoline, and sn-glycero-3-phosphocholine, ultimately forming sn-glycerol-3-phosphate. Experimental evidence has proven that the sn-glycerol-3-phosphate enables plants to develop systemic acquired resistance (SAR) through the enzymatic activity of glycerophosphodiester phosphodiesterase 1. This process plays a crucial role in plant responses to abiotic stress (Liao et al., 2024). Furthermore, recent research has shown that the chemical sn-glycerol-3-phosphate serves as a substrate for the biosynthesis of extracellular lipids, such as suberin and cutin in plants, through acylation processes. This enhances the physical defense ability of the plant surface layer, thereby enabling the plant to resist invasion by exogenous pathogenic bacteria or fungi (Zhang et al., 2024). These findings further imply that the *R. decorum* Franch. corollas at a lower distribution altitude (Heqing) may face greater pressures to adapt to both biotic and abiotic environmental stresses. Nevertheless, based on the comprehensive analysis of DEGs/DAMs, no additional significantly enriched functional pathways were found in the edible corollas from different regions.

### 4.3 Benefits and risk of the consumption towards fresh corollas and edible dried corollas

Through the exploration of comparative network analysis of DAMs between HQ and LF regions, it was found that, in comparison with fresh corollas, there was a significant decrease in the levels of the majority of amino acids regulated and produced via the aminoacyl-tRNA biosynthesis pathway in edible dry corollas. However, L-histidine was successfully retained. L-histidine had been proven to be able to tolerate a high decomposition temperature of 287°C, indicating excellent thermal stability and the ability to resist decomposition or denaturation during cooking processes. As an essential amino acid, L-histidine was crucial for proton buffering, metal ion chelation, scavenging reactive oxygen and nitrogen species, erythropoiesis, and the histaminergic system in the human body (Holeček, 2020). Furthermore, research has indicated its impact on food flavor. Specifically, it is a major factor contributing to bitter and harsh taste characteristics. This property may have a negative effect on the palatability of *R. decorum* Franch. when it was prepared as a cooked dish (Zheng et al., 2024). L-cysteine sulfide derivatives, in which one sulfur atom is replaced by a 4-methylthiobutylthiohydroximoyl group, have been identified as S-(4-methylthiobutylthiohydroximoyl)-L-cysteine in the edible dried corollas of *R. decorum* Franch. To the best of our knowledge, no relevant studies had been reported on this particular amino acid derivative. Nevertheless, other L-cysteine sulfide derivatives with similar structures, such as S-allyl-L-cysteine, were known to possess a wide range of biological activities, including antioxidant, anti-inflammatory, and anticancer properties (Kosuge, 2020). Based on these findings, it is hypothesized that the chemical S-(4-methylthiobutylthiohydroximoyl)-L-cysteine may serve as a potential novel active medicine for further development. During thermal processing, L-cysteine sulfide derivatives can undergo sulfoxidation to produce sulfoxide compounds with distinctive aromatic profiles (Steventon and Mitchell, 2009), thereby enhancing the flavor of food. This characteristic may also explain why the Bai people traditionally select *R. decorum* Franch. as a culinary ingredient. Flavonoid biosynthesis has been proven to play a crucial role in the resistance of *R. decorum* Franch. to pests, diseases, and herbivores (Liu et al., 2024a, 2025). Moreover, it contains numerous metabolites that are beneficial to human health. However, in comparison with the fresh corollas, the composition of flavonoids in the processed edible dried corollas has changed significantly. Several flavonoid compounds, such as (−)-epigallocatechin, myricetin, (+)-gallocatechin, and tricetin, have shown substantial decreases. This presents a challenge when considering the fresh corollas of *R. decorum* Franch. as a source for drug development, thus necessitating consideration of the limitations of traditional processing methods. In contrast, the content of certain flavonoids with low water solubility and high thermal stability, such as p-coumaroyl quinic acid and taxifolin, exhibited an increase in the edible dried corollas when compared to the fresh corollas. p-coumaroyl quinic acid, in addition to being industrially synthesized via condensation reactions, occurs naturally in various plants (Bakr and El Bishbishy, 2016; Chen et al., 2014; Teclegeorgish et al., 2021). It combats aging by elevating telomerase and telomerase reverse transcriptase levels in normal human melanocytes, demonstrating potential for the development of drug and functional food (Saber et al., 2021). Taxifolin possesses anti-oxidative and anti-inflammatory properties, offering protection against advanced glycation end products and mitochondrial damage (Saito et al., 2021). Moreover, taxifolin has been proven to be effective in the cardiovascular system by reducing blood pressure (Bernatova and Liskova, 2021), making a positive contribution to the prevention of basic cardiovascular and cerebrovascular diseases among the Bai people residing on the Yunnan Plateau.

Overall, the Bai ethnic group has traditionally utilized the processed dried corollas of *R. decorum* Franch. in culinary practices. In addition to enhancing the flavor of food, this ingredient may also have certain medicinal benefits. However, the edible dried corollas contain grayanotoxin, which may lead to human fatality if not properly decomposed (Baral et al., 2022; Yang et al., 2022). In Yunnan province, numerous cases of poisoning caused by *Rhododendron* consumption are reported every year. Therefore, it is necessary to establish standardized procedures for the collection, processing, sales, and online promotion of *Rhododendron*. It is anticipated that this valuable traditional ethnic cuisine will become available across China and globally in the future.

## 5 Conclusion

The corollas of *R. decorum* Franch. were extensively documented to contain toxic secondary metabolites; however, the Bai nationality in Yunnan, China, have safely consumed them as a traditional food for centuries. By employing integrated transcriptomic and metabolomic analysis, we demonstrated that the long-standing practice of the Bai people of removing the androecium and gynoecium prior to consumption is a precise, source-directed risk mitigation strategy. The androecium/gynoecium serve as the primary accumulation sites of potentially toxic flavonoids, and flavonoid biosynthesis represents the most significantly divergent pathway in both transcription and metabolism between floral organs. Further analysis also disclosed that the traditional soaking and drying process yields a triple synergistic effect: it decreases residual toxic flavonoids, preserves metabolites that define flavor, and enriches bioactive components beneficial to human health. This study systematically understands the molecular mechanism underlying this traditional ethnic edible practice, thereby establishing a replicable paradigm for the safety assessment and standardized utilization of characteristic ethnic edible plant resources.

## Data Availability

The data presented in this paper are deposited in the GenomeSequence Archive (Genomics, Proteomics & Bioinformatics 2025) in NationalGenomics Data Center (Nucleic Acids Res 2025), China National Center forBioinformation / Beijing Institute of Genomics, Chinese Academy of Sciences,accession number : CRA043725 that are publicly accessible at https://ngdc.cncb.ac.cn/gsa/browse/CRA043725.
