# Supplementary material for: Dual modulation of toxic risks and functional benefits: How traditional practices enable safe consumption of toxic Rhododendron decorum Franch. in ethnic cuisine
Source: Front Plant Sci. 2026 Jun 9;17:1778014. doi: 10.3389/fpls.2026.1778014 (PMC13291839; doi:10.3389/fpls.2026.1778014)
Supplement: Supplementary file 1 [file Table1.docx]

Supplementary Material

# 1 Supplementary Figures and Tables

##
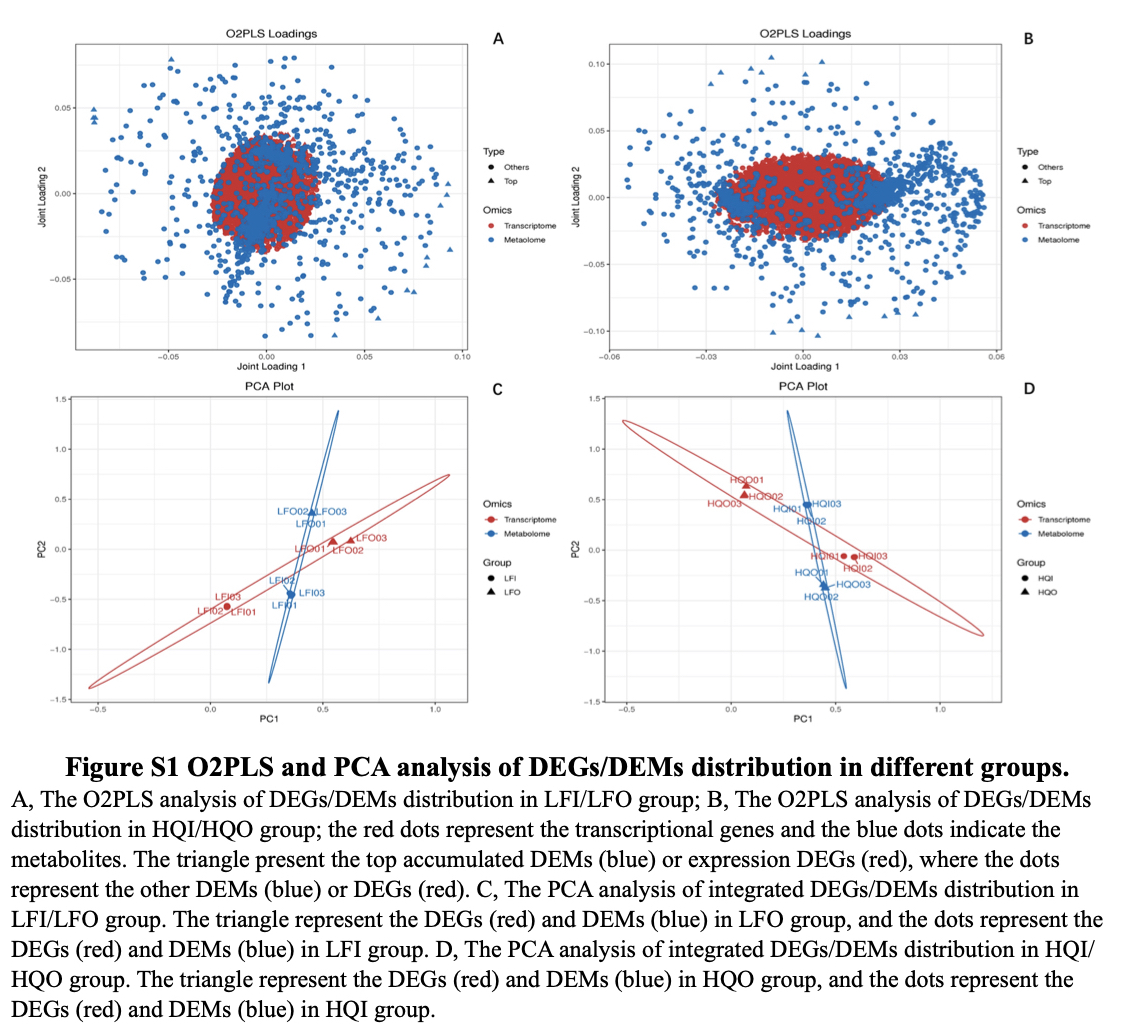
Supplementary Figures

**Supplementary Figure 1. O2PLS and PCA analysis of DEGS/DAMs distribution in different groups.**

1. The O2PLS analysis of DEGs/DAMs distribution in LFI/LFO group. (B) The O2PLS analysis of DEGS/DAMs distribution in HQI/HQO group; the red dots represent the transcriptional genes and the blue dots indicate the metabolites. The triangle present the top accumulated DAMs (blue) or expression DEGs (red), where the dots represent the other DAMs (blue) or DEGs (red). (C) The PCA analysis of integrated DEGs/DAMs distribution in LFILFO group. The triangle represent the DEGs (red) and DAMs (blue) in LFO group, and the dots represent the DEGs (red) and DAMs (blue) in LFI group. (D) The PCA analysis of integrated DEGs/DAMs distribution in HQI/ HQO group. The triangle represent the DEGs (red) and DAMs (blue) in HQO group, and the dots represent the DEGs (red) and DAMs (blue) in HQI group.


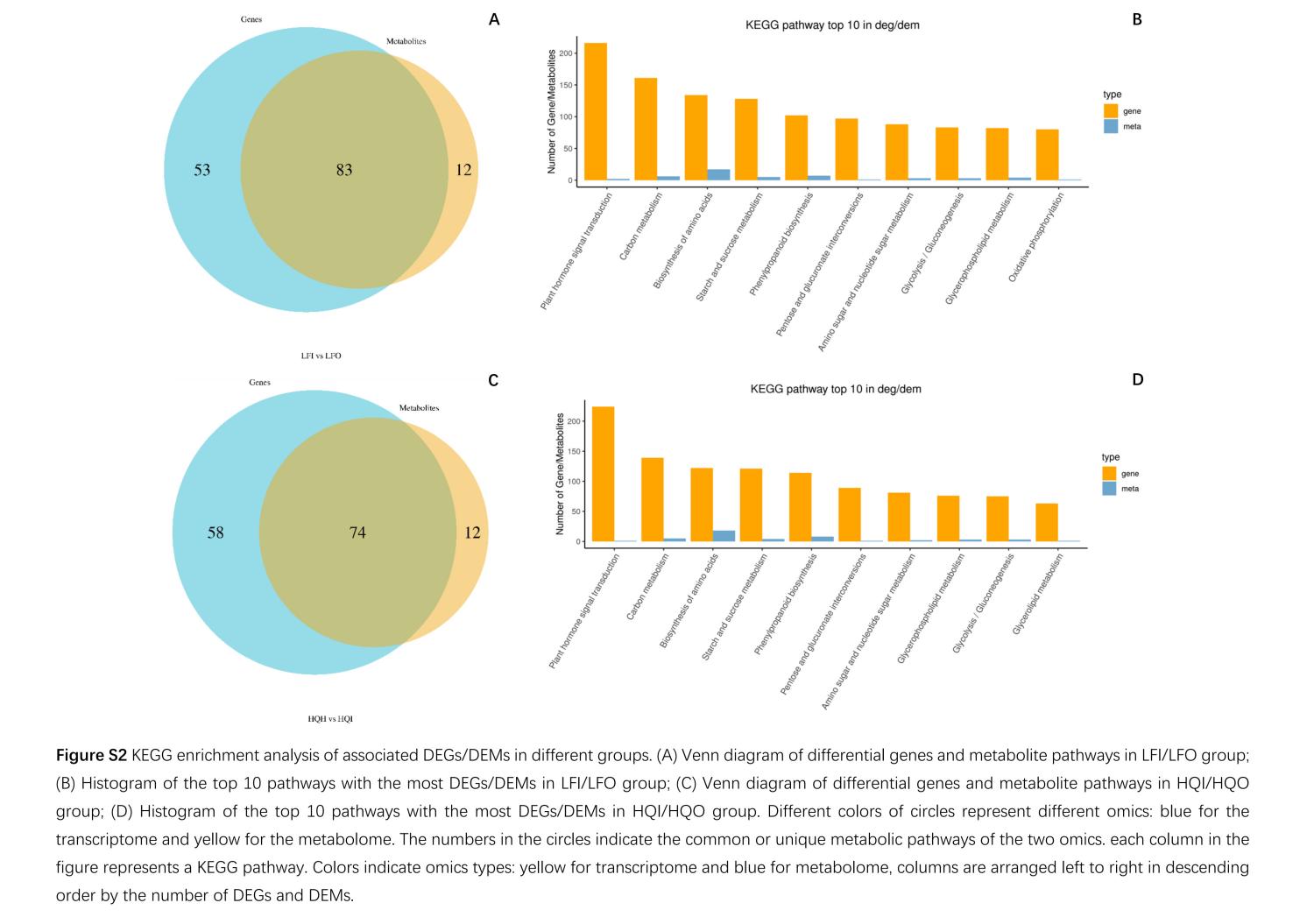


**Supplementary Figure 2.** **KEGG enrichment analysis of associated DEGs/DAMs in different groups.**

(A) Ven diagram of differential genes and metabolite pathways in LFI/LFO group; (B) Histogram of the top 10 pathways with the most DEGs/DAMs in LFI/LFO group; (C) Ven diagram of differential genes and metabolite pathways in HQ1/HQO group; (D) Histogram of the top 10 pathways with the most DEGs/DAMs in HQI/HQO group. Different colors of circles represent different omics: blue for the transcriptome and yellow for the metabolome. The numbers in the circles indicate the common or unique metabolic pathways of the two omics. each column in the figure represents a KEGG pathway. Colors indicate omics types: yellow for transcriptome and blue for metabolome, columns are arranged left to right in descending order by the number of DEGs and DAMs.


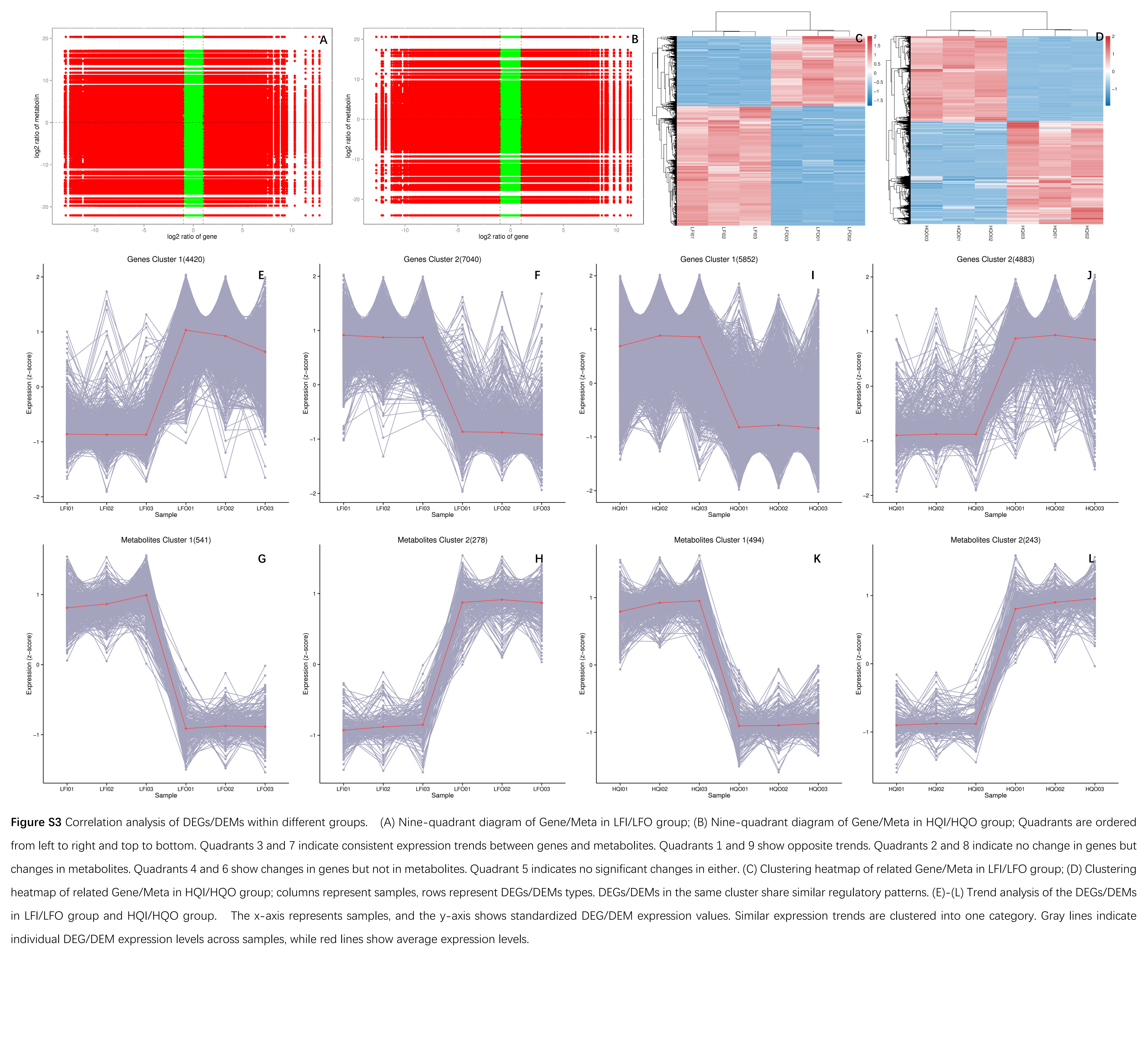


**Supplementary Figure 3. Correlation analysis of DEGs/DAMs within different groups.**

(A) Nine-quadrant diagram of Gene/Meta in LF/LFO group. (B) Nine-quadrant diagram of Gene/Meta in HQI/HQO group. Quadrants are ordered from left to right and top to bottom. Quadrants 3 and 7 indicate consistent expression trends between genes and metabolites. Quadrants 1 and 9 show opposite trends. Quadrants 2 and 8 indicate no change in genes but changes in metabolites. Quadrants 4 and 6 show changes in genes but not in metabolites. Quadrant 5 indicates no significant changes in either. (C) Clustering heatmap of related Gene/Meta in LFI/LFO group; (D) Clustering heatmap of related Gene/Meta in HO/HOO aroup: columns represent samples, rows represent DEGs/DAMs tves. DEGs/DAMs in the same cluster share similar requlatorv patterns. (E)-(L) Trend analvsis of the DEGS/DAMs in LFI/LFO group and HQI/HQO group. The x-axis represents samples and the y-axis shows standardized DEG/DAM expression values. Similar expression trends are grouped into one category. Gray lines indicate individual DEG/DAM expression levels across samples, while red lines show average expression levels.


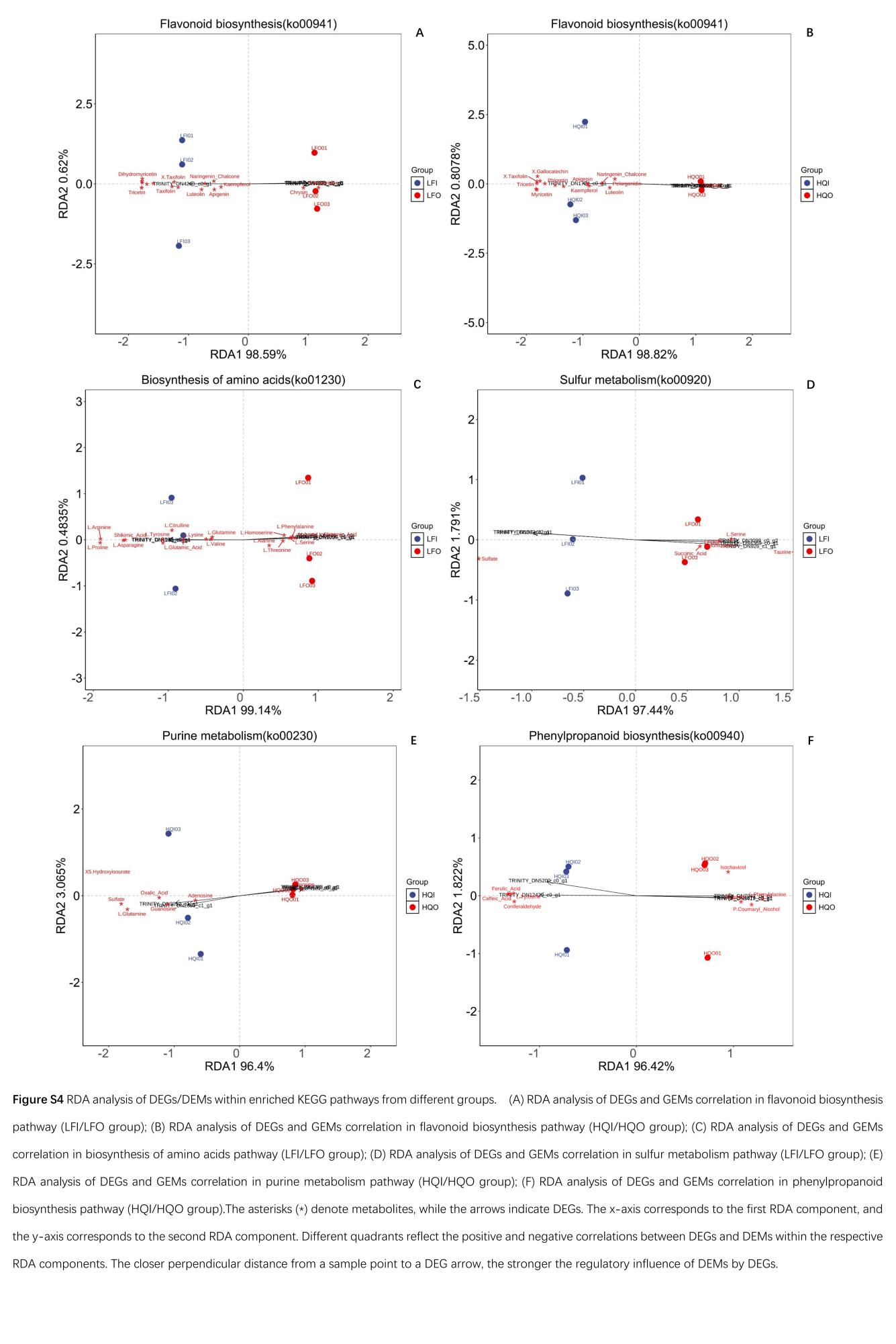


Figure S4 RDA analvsis of **Supplementary Figure 4. RDA analysis of DEGs/DAMs within enriched KEGG pathways from different groups.**

(A) RDA analvsis of DEGs and DAMs correlation in flavonoid biosynthesis pathway (LFI/LFO group). (B) RDA analysis of DEGs and DAMs correlation in flavonoid biosynthesis pathway (HQI/HQO group). (C) DA analysis of DEGs and DAMs correlation in biosynthesis of amino acids pathway (LFI/LFO group). (D) RDA analvsis of DEGs and DAMs correlation in sulfur metabolism pathwav (LFI/LFO group). (E) RDA analvsis of DEGs and DAMs correlation in purine metabolism pathway (HQI/HQO group) (F) DA analvsis of DEGs and DAMs correlation in phenvloronanoid biosynthesis pathway (HQI/HQO group). The asterisks (*) denote metabolites, while the arrows indicate DEGs. The x-axis corresponds to the first RDA component, and the y-axis corresponds to the second RDA component. Different quadrants reflect the positive and negative correlations between DEGs and DAMs within the respective RDA components. The closer perpendicular distance from a sample point to a DEG arrow, the stronger the regulatory influence of DAMs by DEGS.


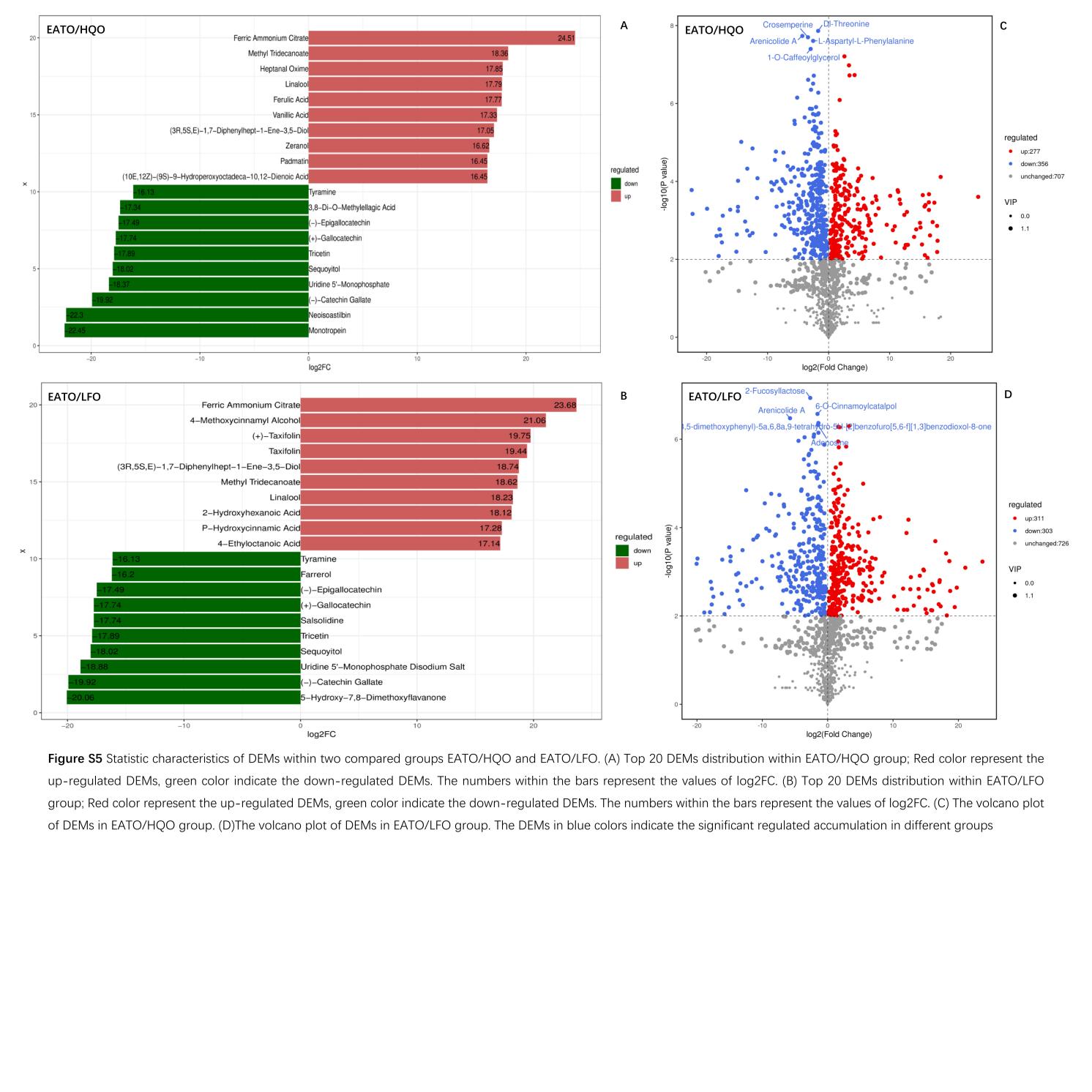


**Supplementary Figure 5 Statistic characteristics of DAMs within two compared groups EATO/HQ0 and EATO/LFO.**

1. Top 20 DAMs distribution within EATO/HQO group; Red color represent the up-regulated DAMs. green color indicate the down-regulated DAMs. The numbers within the bars represent the values of log2FC. (B) Top 20 DAMs distribution within EATO/LFO group: Red color represent the up-regulated DAMs, green color indicate the down-regulated DAMs. The numbers within the bars represent the values of log2FC. (C) The volcano plot of DAMs in EATO/HOO group. (D)The volcano plot of DAMs in EATO/LFO group. The DAMs in blue colors indicate the significant regulated accumulation in different groups.

## Supplementary Tables

**Table S1 Comparative KEGG enrichment analysis of DEGs/DAMs correlation analysis in LFI/LFO group**

| **Kegg_pathway** | **ko_id** | **Gene_Cluter_frequency** | **Gene_Genome_frequency** | **Gene_P-value** | **Gene_rich_factor** | **Metabolite_Cluter_frequency** | **Metabolite_Genome_frequency** | **Metabolite_P-value** | **Metabolite_rich_factor** |
| --- | --- | --- | --- | --- | --- | --- | --- | --- | --- |
| Glycerophospholipid metabolism | ko00564 | 82 out of 3463 2.36788911348542% | 164 out of 10163 1.61369674308767% | 0.0000 | 1.47 | 4 out of 165 2.42424242424242% | 5 out of 264 1.89393939393939% | 0.3797 | 1.28 |
| Glycerolipid metabolism | ko00561 | 69 out of 3463 1.99249205890846% | 135 out of 10163 1.32834792876119% | 0.0000 | 1.50 | 1 out of 165 0.606060606060606% | 1 out of 264 0.378787878787879% | 0.6250 | 1.60 |
| Inositol phosphate metabolism | ko00562 | 65 out of 3463 1.87698527288478% | 133 out of 10163 1.30866870018695% | 0.0003 | 1.43 | 4 out of 165 2.42424242424242% | 4 out of 264 1.51515151515152% | 0.1505 | 1.60 |
| Pentose and glucuronate interconversions | ko00040 | 97 out of 3463 2.80103956107421% | 220 out of 10163 2.16471514316639% | 0.0012 | 1.29 | 1 out of 165 0.606060606060606% | 5 out of 264 1.89393939393939% | 0.9930 | 0.32 |
| Ubiquinone and other terpenoid-quinone biosynthesis | ko00130 | 32 out of 3463 0.924054288189431% | 60 out of 10163 0.590376857227197% | 0.0016 | 1.57 | 3 out of 165 1.81818181818182% | 4 out of 264 1.51515151515152% | 0.5184 | 1.20 |
| Cysteine and methionine metabolism | ko00270 | 66 out of 3463 1.9058619693907% | 143 out of 10163 1.40706484305815% | 0.0017 | 1.35 | 5 out of 165 3.03030303030303% | 8 out of 264 3.03030303030303% | 0.6530 | 1.00 |
| Phosphatidylinositol signaling system | ko04070 | 57 out of 3463 1.64597170083742% | 126 out of 10163 1.23979140017711% | 0.0058 | 1.33 | 2 out of 165 1.21212121212121% | 2 out of 264 0.757575757575758% | 0.3897 | 1.60 |
| Carbon fixation in photosynthetic organisms | ko00710 | 51 out of 3463 1.47271152180191% | 111 out of 10163 1.09219718587031% | 0.0061 | 1.35 | 3 out of 165 1.81818181818182% | 4 out of 264 1.51515151515152% | 0.5184 | 1.20 |
| Folate biosynthesis | ko00790 | 19 out of 3463 0.548657233612475% | 34 out of 10163 0.334546885762078% | 0.0073 | 1.64 | 1 out of 165 0.606060606060606% | 2 out of 264 0.757575757575758% | 0.8603 | 0.80 |
| Riboflavin metabolism | ko00740 | 16 out of 3463 0.462027144094716% | 28 out of 10163 0.275509200039358% | 0.0102 | 1.68 | 1 out of 165 0.606060606060606% | 2 out of 264 0.757575757575758% | 0.8603 | 0.80 |
| Phenylpropanoid biosynthesis | ko00940 | 102 out of 3463 2.94542304360381% | 249 out of 10163 2.45006395749287% | 0.0129 | 1.20 | 7 out of 165 4.24242424242424% | 10 out of 264 3.78787878787879% | 0.4448 | 1.12 |
| Phenylalanine, tyrosine and tryptophan biosynthesis | ko00400 | 32 out of 3463 0.924054288189431% | 68 out of 10163 0.669093771524156% | 0.0178 | 1.38 | 4 out of 165 2.42424242424242% | 4 out of 264 1.51515151515152% | 0.1505 | 1.60 |
| Starch and sucrose metabolism | ko00500 | 128 out of 3463 3.69621715275772% | 325 out of 10163 3.19787464331398% | 0.0240 | 1.16 | 5 out of 165 3.03030303030303% | 7 out of 264 2.65151515151515% | 0.4743 | 1.14 |
| Glycine, serine and threonine metabolism | ko00260 | 43 out of 3463 1.24169794975455% | 98 out of 10163 0.964282200137755% | 0.0271 | 1.29 | 5 out of 165 3.03030303030303% | 7 out of 264 2.65151515151515% | 0.4743 | 1.14 |
| Carbon metabolism | ko01200 | 161 out of 3463 4.64914813745307% | 419 out of 10163 4.12279838630326% | 0.0318 | 1.13 | 6 out of 165 3.63636363636364% | 9 out of 264 3.40909090909091% | 0.5458 | 1.07 |
| Biosynthesis of amino acids | ko01230 | 134 out of 3463 3.86947733179324% | 345 out of 10163 3.39466692905638% | 0.0336 | 1.14 | 17 out of 165 10.3030303030303% | 21 out of 264 7.95454545454545% | 0.0523 | 1.30 |
| Tyrosine metabolism | ko00350 | 39 out of 3463 1.12619116373087% | 89 out of 10163 0.875725671553675% | 0.0349 | 1.29 | 4 out of 165 2.42424242424242% | 6 out of 264 2.27272727272727% | 0.5965 | 1.07 |
| Arginine and proline metabolism | ko00330 | 38 out of 3463 1.09731446722495% | 87 out of 10163 0.856046442979435% | 0.0389 | 1.28 | 4 out of 165 2.42424242424242% | 4 out of 264 1.51515151515152% | 0.1505 | 1.60 |
| Tropane, piperidine and pyridine alkaloid biosynthesis | ko00960 | 18 out of 3463 0.519780537106555% | 37 out of 10163 0.364065728623438% | 0.0470 | 1.43 | 3 out of 165 1.81818181818182% | 6 out of 264 2.27272727272727% | 0.8562 | 0.80 |
| Flavonoid biosynthesis | ko00941 | 50 out of 3463 1.44383482529599% | 121 out of 10163 1.19059332874151% | 0.0568 | 1.21 | 17 out of 165 10.3030303030303% | 18 out of 264 6.81818181818182% | 0.0019 | 1.51 |
| Phenylalanine metabolism | ko00360 | 33 out of 3463 0.952930984695351% | 77 out of 10163 0.757650300108236% | 0.0670 | 1.26 | 6 out of 165 3.63636363636364% | 7 out of 264 2.65151515151515% | 0.1901 | 1.37 |
| Glutathione metabolism | ko00480 | 61 out of 3463 1.7614784868611% | 153 out of 10163 1.50546098592935% | 0.0764 | 1.17 | 1 out of 165 0.606060606060606% | 1 out of 264 0.378787878787879% | 0.6250 | 1.60 |
| alpha-Linolenic acid metabolism | ko00592 | 37 out of 3463 1.06843777071903% | 90 out of 10163 0.885565285840795% | 0.0975 | 1.21 | 3 out of 165 1.81818181818182% | 6 out of 264 2.27272727272727% | 0.8562 | 0.80 |
| Sulfur metabolism | ko00920 | 18 out of 3463 0.519780537106555% | 40 out of 10163 0.393584571484798% | 0.0995 | 1.32 | 5 out of 165 3.03030303030303% | 5 out of 264 1.89393939393939% | 0.0932 | 1.60 |
| Nicotinate and nicotinamide metabolism | ko00760 | 18 out of 3463 0.519780537106555% | 41 out of 10163 0.403424185771918% | 0.1229 | 1.29 | 3 out of 165 1.81818181818182% | 4 out of 264 1.51515151515152% | 0.5184 | 1.20 |
| Isoquinoline alkaloid biosynthesis | ko00950 | 21 out of 3463 0.606410626624314% | 49 out of 10163 0.482141100068877% | 0.1260 | 1.26 | 3 out of 165 1.81818181818182% | 4 out of 264 1.51515151515152% | 0.5184 | 1.20 |
| Terpenoid backbone biosynthesis | ko00900 | 34 out of 3463 0.981807681201271% | 84 out of 10163 0.826527600118075% | 0.1303 | 1.19 | 1 out of 165 0.606060606060606% | 2 out of 264 0.757575757575758% | 0.8603 | 0.80 |
| Fatty acid elongation | ko00062 | 19 out of 3463 0.548657233612475% | 44 out of 10163 0.432943028633278% | 0.1324 | 1.27 | 1 out of 165 0.606060606060606% | 1 out of 264 0.378787878787879% | 0.6250 | 1.60 |
| Cutin, suberine and wax biosynthesis | ko00073 | 25 out of 3463 0.721917412647993% | 60 out of 10163 0.590376857227197% | 0.1345 | 1.22 | 3 out of 165 1.81818181818182% | 3 out of 264 1.13636363636364% | 0.2425 | 1.60 |
| Plant hormone signal transduction | ko04075 | 216 out of 3463 6.23736644527866% | 597 out of 10163 5.87424972941061% | 0.1414 | 1.06 | 2 out of 165 1.21212121212121% | 2 out of 264 0.757575757575758% | 0.3897 | 1.60 |
| Porphyrin and chlorophyll metabolism | ko00860 | 26 out of 3463 0.750794109153913% | 63 out of 10163 0.619895700088557% | 0.1415 | 1.21 | 4 out of 165 2.42424242424242% | 5 out of 264 1.89393939393939% | 0.3797 | 1.28 |
| Carotenoid biosynthesis | ko00906 | 23 out of 3463 0.664164019636154% | 55 out of 10163 0.541178785791597% | 0.1421 | 1.23 | 1 out of 165 0.606060606060606% | 2 out of 264 0.757575757575758% | 0.8603 | 0.80 |
| Taurine and hypotaurine metabolism | ko00430 | 4 out of 3463 0.115506786023679% | 7 out of 10163 0.0688773000098396% | 0.1848 | 1.68 | 3 out of 165 1.81818181818182% | 4 out of 264 1.51515151515152% | 0.5184 | 1.20 |
| Glycolysis / Gluconeogenesis | ko00010 | 83 out of 3463 2.39676580999134% | 224 out of 10163 2.20407360031487% | 0.1890 | 1.09 | 3 out of 165 1.81818181818182% | 3 out of 264 1.13636363636364% | 0.2425 | 1.60 |
| Limonene and pinene degradation | ko00903 | 6 out of 3463 0.173260179035518% | 12 out of 10163 0.118075371445439% | 0.1928 | 1.47 | 1 out of 165 0.606060606060606% | 2 out of 264 0.757575757575758% | 0.8603 | 0.80 |
| Ether lipid metabolism | ko00565 | 23 out of 3463 0.664164019636154% | 57 out of 10163 0.560858014365837% | 0.1932 | 1.18 | 1 out of 165 0.606060606060606% | 1 out of 264 0.378787878787879% | 0.6250 | 1.60 |
| Monoterpenoid biosynthesis | ko00902 | 17 out of 3463 0.490903840600635% | 41 out of 10163 0.403424185771918% | 0.2004 | 1.22 | 1 out of 165 0.606060606060606% | 4 out of 264 1.51515151515152% | 0.9810 | 0.40 |
| Sphingolipid metabolism | ko00600 | 32 out of 3463 0.924054288189431% | 82 out of 10163 0.806848371543835% | 0.2015 | 1.15 | 1 out of 165 0.606060606060606% | 1 out of 264 0.378787878787879% | 0.6250 | 1.60 |
| Linoleic acid metabolism | ko00591 | 9 out of 3463 0.259890268553278% | 20 out of 10163 0.196792285742399% | 0.2106 | 1.32 | 3 out of 165 1.81818181818182% | 4 out of 264 1.51515151515152% | 0.5184 | 1.20 |
| Ascorbate and aldarate metabolism | ko00053 | 48 out of 3463 1.38608143228415% | 127 out of 10163 1.24963101446423% | 0.2120 | 1.11 | 2 out of 165 1.21212121212121% | 3 out of 264 1.13636363636364% | 0.6843 | 1.07 |
| Thiamine metabolism | ko00730 | 16 out of 3463 0.462027144094716% | 39 out of 10163 0.383744957197678% | 0.2249 | 1.20 | 1 out of 165 0.606060606060606% | 1 out of 264 0.378787878787879% | 0.6250 | 1.60 |
| Sulfur relay system | ko04122 | 5 out of 3463 0.144383482529599% | 10 out of 10163 0.0983961428711995% | 0.2285 | 1.47 | 1 out of 165 0.606060606060606% | 1 out of 264 0.378787878787879% | 0.6250 | 1.60 |
| Valine, leucine and isoleucine degradation | ko00280 | 38 out of 3463 1.09731446722495% | 100 out of 10163 0.983961428711994% | 0.2323 | 1.12 | 2 out of 165 1.21212121212121% | 5 out of 264 1.89393939393939% | 0.9327 | 0.64 |
| Glyoxylate and dicarboxylate metabolism | ko00630 | 54 out of 3463 1.55934161131966% | 145 out of 10163 1.42674407163239% | 0.2338 | 1.09 | 7 out of 165 4.24242424242424% | 8 out of 264 3.03030303030303% | 0.1312 | 1.40 |
| Stilbenoid, diarylheptanoid and gingerol biosynthesis | ko00945 | 23 out of 3463 0.664164019636154% | 59 out of 10163 0.580537242940077% | 0.2521 | 1.14 | 1 out of 165 0.606060606060606% | 1 out of 264 0.378787878787879% | 0.6250 | 1.60 |
| Fatty acid degradation | ko00071 | 37 out of 3463 1.06843777071903% | 99 out of 10163 0.974121814424875% | 0.2755 | 1.10 | 2 out of 165 1.21212121212121% | 2 out of 264 0.757575757575758% | 0.3897 | 1.60 |
| Betalain biosynthesis | ko00965 | 10 out of 3463 0.288766965059197% | 24 out of 10163 0.236150742890879% | 0.2795 | 1.22 | 1 out of 165 0.606060606060606% | 2 out of 264 0.757575757575758% | 0.8603 | 0.80 |
| Fructose and mannose metabolism | ko00051 | 38 out of 3463 1.09731446722495% | 102 out of 10163 1.00364065728623% | 0.2799 | 1.09 | 4 out of 165 2.42424242424242% | 7 out of 264 2.65151515151515% | 0.7596 | 0.91 |
| Lysine degradation | ko00310 | 25 out of 3463 0.721917412647993% | 66 out of 10163 0.649414542949916% | 0.2969 | 1.11 | 4 out of 165 2.42424242424242% | 6 out of 264 2.27272727272727% | 0.5965 | 1.07 |
| Pyruvate metabolism | ko00620 | 51 out of 3463 1.47271152180191% | 140 out of 10163 1.37754600019679% | 0.3055 | 1.07 | 2 out of 165 1.21212121212121% | 2 out of 264 0.757575757575758% | 0.3897 | 1.60 |
| Pyrimidine metabolism | ko00240 | 39 out of 3463 1.12619116373087% | 108 out of 10163 1.06267834300895% | 0.3609 | 1.06 | 6 out of 165 3.63636363636364% | 8 out of 264 3.03030303030303% | 0.3670 | 1.20 |
| Alanine, aspartate and glutamate metabolism | ko00250 | 25 out of 3463 0.721917412647993% | 68 out of 10163 0.669093771524156% | 0.3621 | 1.08 | 5 out of 165 3.03030303030303% | 6 out of 264 2.27272727272727% | 0.2713 | 1.33 |
| Biotin metabolism | ko00780 | 9 out of 3463 0.259890268553278% | 23 out of 10163 0.226311128603759% | 0.3773 | 1.15 | 2 out of 165 1.21212121212121% | 2 out of 264 0.757575757575758% | 0.3897 | 1.60 |
| Monobactam biosynthesis | ko00261 | 5 out of 3463 0.144383482529599% | 12 out of 10163 0.118075371445439% | 0.3898 | 1.22 | 5 out of 165 3.03030303030303% | 6 out of 264 2.27272727272727% | 0.2713 | 1.33 |
| Pantothenate and CoA biosynthesis | ko00770 | 16 out of 3463 0.462027144094716% | 44 out of 10163 0.432943028633278% | 0.4294 | 1.07 | 1 out of 165 0.606060606060606% | 2 out of 264 0.757575757575758% | 0.8603 | 0.80 |
| Phosphonate and phosphinate metabolism | ko00440 | 3 out of 3463 0.0866300895177592% | 7 out of 10163 0.0688773000098396% | 0.4464 | 1.26 | 1 out of 165 0.606060606060606% | 2 out of 264 0.757575757575758% | 0.8603 | 0.80 |
| Amino sugar and nucleotide sugar metabolism | ko00520 | 88 out of 3463 2.54114929252094% | 257 out of 10163 2.52878087178983% | 0.5011 | 1.00 | 3 out of 165 1.81818181818182% | 6 out of 264 2.27272727272727% | 0.8562 | 0.80 |
| ABC transporters | ko02010 | 64 out of 3463 1.84810857637886% | 187 out of 10163 1.84000787169143% | 0.5104 | 1.00 | 27 out of 165 16.3636363636364% | 39 out of 264 14.7727272727273% | 0.2248 | 1.11 |
| Flavone and flavonol biosynthesis | ko00944 | 11 out of 3463 0.317643661565117% | 32 out of 10163 0.314867657187838% | 0.5520 | 1.01 | 9 out of 165 5.45454545454545% | 12 out of 264 4.54545454545455% | 0.2770 | 1.20 |
| Indole alkaloid biosynthesis | ko00901 | 2 out of 3463 0.0577533930118394% | 5 out of 10163 0.0491980714355997% | 0.5537 | 1.17 | 1 out of 165 0.606060606060606% | 1 out of 264 0.378787878787879% | 0.6250 | 1.60 |
| Fatty acid biosynthesis | ko00061 | 25 out of 3463 0.721917412647993% | 74 out of 10163 0.728131457246876% | 0.5648 | 0.99 | 3 out of 165 1.81818181818182% | 4 out of 264 1.51515151515152% | 0.5184 | 1.20 |
| Caffeine metabolism | ko00232 | 1 out of 3463 0.0288766965059197% | 2 out of 10163 0.0196792285742399% | 0.5654 | 1.47 | 1 out of 165 0.606060606060606% | 1 out of 264 0.378787878787879% | 0.6250 | 1.60 |
| Arginine biosynthesis | ko00220 | 11 out of 3463 0.317643661565117% | 33 out of 10163 0.324707271474958% | 0.6006 | 0.98 | 5 out of 165 3.03030303030303% | 6 out of 264 2.27272727272727% | 0.2713 | 1.33 |
| Purine metabolism | ko00230 | 55 out of 3463 1.58821830782558% | 165 out of 10163 1.62353635737479% | 0.6092 | 0.98 | 11 out of 165 6.66666666666667% | 12 out of 264 4.54545454545455% | 0.0265 | 1.47 |
| Vitamin B6 metabolism | ko00750 | 7 out of 3463 0.202136875541438% | 21 out of 10163 0.206631900029519% | 0.6095 | 0.98 | 2 out of 165 1.21212121212121% | 3 out of 264 1.13636363636364% | 0.6843 | 1.07 |
| Lysine biosynthesis | ko00300 | 5 out of 3463 0.144383482529599% | 15 out of 10163 0.147594214306799% | 0.6196 | 0.98 | 2 out of 165 1.21212121212121% | 3 out of 264 1.13636363636364% | 0.6843 | 1.07 |
| Cyanoamino acid metabolism | ko00460 | 27 out of 3463 0.779670805659832% | 82 out of 10163 0.806848371543835% | 0.6277 | 0.97 | 5 out of 165 3.03030303030303% | 7 out of 264 2.65151515151515% | 0.4743 | 1.14 |
| Butanoate metabolism | ko00650 | 13 out of 3463 0.375397054576956% | 40 out of 10163 0.393584571484798% | 0.6412 | 0.95 | 3 out of 165 1.81818181818182% | 3 out of 264 1.13636363636364% | 0.2425 | 1.60 |
| Nitrogen metabolism | ko00910 | 13 out of 3463 0.375397054576956% | 42 out of 10163 0.413263800059038% | 0.7186 | 0.91 | 2 out of 165 1.21212121212121% | 2 out of 264 0.757575757575758% | 0.3897 | 1.60 |
| 2-Oxocarboxylic acid metabolism | ko01210 | 23 out of 3463 0.664164019636154% | 73 out of 10163 0.718291842959756% | 0.7189 | 0.92 | 8 out of 165 4.84848484848485% | 11 out of 264 4.16666666666667% | 0.3546 | 1.16 |
| Citrate cycle (TCA cycle) | ko00020 | 27 out of 3463 0.779670805659832% | 86 out of 10163 0.846206828692315% | 0.7367 | 0.92 | 2 out of 165 1.21212121212121% | 2 out of 264 0.757575757575758% | 0.3897 | 1.60 |
| Fatty acid metabolism | ko01212 | 40 out of 3463 1.15506786023679% | 126 out of 10163 1.23979140017711% | 0.7401 | 0.93 | 1 out of 165 0.606060606060606% | 2 out of 264 0.757575757575758% | 0.8603 | 0.80 |
| Histidine metabolism | ko00340 | 12 out of 3463 0.346520358071037% | 40 out of 10163 0.393584571484798% | 0.7587 | 0.88 | 1 out of 165 0.606060606060606% | 4 out of 264 1.51515151515152% | 0.9810 | 0.40 |
| Galactose metabolism | ko00052 | 48 out of 3463 1.38608143228415% | 152 out of 10163 1.49562137164223% | 0.7692 | 0.93 | 8 out of 165 4.84848484848485% | 14 out of 264 5.3030303030303% | 0.7636 | 0.91 |
| Selenocompound metabolism | ko00450 | 6 out of 3463 0.173260179035518% | 23 out of 10163 0.226311128603759% | 0.8488 | 0.77 | 1 out of 165 0.606060606060606% | 1 out of 264 0.378787878787879% | 0.6250 | 1.60 |
| Biosynthesis of unsaturated fatty acids | ko01040 | 11 out of 3463 0.317643661565117% | 44 out of 10163 0.432943028633278% | 0.9271 | 0.73 | 3 out of 165 1.81818181818182% | 3 out of 264 1.13636363636364% | 0.2425 | 1.60 |
| C5-Branched dibasic acid metabolism | ko00660 | 2 out of 3463 0.0577533930118394% | 11 out of 10163 0.108235757158319% | 0.9318 | 0.53 | 3 out of 165 1.81818181818182% | 3 out of 264 1.13636363636364% | 0.2425 | 1.60 |
| Propanoate metabolism | ko00640 | 19 out of 3463 0.548657233612475% | 72 out of 10163 0.708452228672636% | 0.9365 | 0.77 | 2 out of 165 1.21212121212121% | 2 out of 264 0.757575757575758% | 0.3897 | 1.60 |
| Valine, leucine and isoleucine biosynthesis | ko00290 | 5 out of 3463 0.144383482529599% | 25 out of 10163 0.245990357177999% | 0.9608 | 0.59 | 3 out of 165 1.81818181818182% | 5 out of 264 1.89393939393939% | 0.7264 | 0.96 |
| Glucosinolate biosynthesis | ko00966 | 1 out of 3463 0.0288766965059197% | 11 out of 10163 0.108235757158319% | 0.9898 | 0.27 | 4 out of 165 2.42424242424242% | 6 out of 264 2.27272727272727% | 0.5965 | 1.07 |
| Oxidative phosphorylation | ko00190 | 80 out of 3463 2.31013572047358% | 303 out of 10163 2.98140312899734% | 0.9985 | 0.77 | 1 out of 165 0.606060606060606% | 1 out of 264 0.378787878787879% | 0.6250 | 1.60 |
| Aminoacyl-tRNA biosynthesis | ko00970 | 36 out of 3463 1.03956107421311% | 155 out of 10163 1.52514021450359% | 0.9988 | 0.68 | 12 out of 165 7.27272727272727% | 16 out of 264 6.06060606060606% | 0.2148 | 1.20 |
| Isoflavonoid biosynthesis | ko00943 | 4 out of 3463 0.115506786023679% | 33 out of 10163 0.324707271474958% | 0.9990 | 0.36 | 8 out of 165 4.84848484848485% | 9 out of 264 3.40909090909091% | 0.0893 | 1.42 |
| **Note:**  Kegg_pathway: Designations of pathways (or subcategories) in the KEGG database. ko_id: Pathway IDs in the KEGG database. Gene_Cluter_frequency: The proportion of target functional genes in the differential gene set under the specific pathway. Gene_Genome_frequency: The proportion of target functional genes in the genomic background. Gene_P-value: The corrected p-value of DEGs obtained through Fisher's exact test. Gene_rich_factor: The ratio of pathway-annotated genes among DEGs to that among all genes. Metabolite_Cluter_frequency: The proportion of target functional metabolites in the differential metabolite set under the specific pathway. Metabolite_Genome_frequency: The proportion of target functional metabolites in the metabolic background. Metabolite_P-value: The corrected p-value of DEMs obtained through Fisher's exact test. Metabolite_rich_factor: The ratio of pathway-annotated metabolites among DEMs to that among all metabolites. | | | | | | | | | |

**Table S2 Comparative KEGG enrichment analysis for DEGs/DAMs correlation analysis in HQI/HQO group**

| **Kegg_pathway** | **ko_id** | **Gene_Cluter_frequency** | **Gene_Genome_frequency** | **Gene_P-value** | **Gene_rich_factor** | **Metabolite_Cluter_frequency** | **Metabolite_Genome_frequency** | **Metabolite_P-value** | **Metabolite_rich_factor** |
| --- | --- | --- | --- | --- | --- | --- | --- | --- | --- |
| Glycerophospholipid metabolism | ko00564 | 76 out of 3229 2.35366986683184% | 156 out of 9746 1.60065667966345% | 0.0000 | 1.47 | 3 out of 145 2.06896551724138% | 5 out of 264 1.89393939393939% | 0.5924 | 1.09 |
| Phenylpropanoid biosynthesis | ko00940 | 114 out of 3229 3.53050480024775% | 262 out of 9746 2.68828237225528% | 0.0002 | 1.31 | 8 out of 145 5.51724137931035% | 10 out of 264 3.78787878787879% | 0.0944 | 1.46 |
| Plant hormone signal transduction | ko04075 | 224 out of 3229 6.93713223908331% | 561 out of 9746 5.75620767494357% | 0.0003 | 1.21 | 1 out of 145 0.689655172413793% | 2 out of 264 0.757575757575758% | 0.7978 | 0.91 |
| Glycerolipid metabolism | ko00561 | 63 out of 3229 1.95106844224218% | 133 out of 9746 1.36466242561051% | 0.0004 | 1.43 | 1 out of 145 0.689655172413793% | 1 out of 264 0.378787878787879% | 0.5492 | 1.82 |
| Porphyrin and chlorophyll metabolism | ko00860 | 29 out of 3229 0.898110870238464% | 52 out of 9746 0.533552226554484% | 0.0006 | 1.68 | 4 out of 145 2.75862068965517% | 5 out of 264 1.89393939393939% | 0.2528 | 1.46 |
| Ubiquinone and other terpenoid-quinone biosynthesis | ko00130 | 36 out of 3229 1.11489625270982% | 71 out of 9746 0.728504001641699% | 0.0016 | 1.53 | 3 out of 145 2.06896551724138% | 4 out of 264 1.51515151515152% | 0.3886 | 1.37 |
| Phosphonate and phosphinate metabolism | ko00440 | 8 out of 3229 0.247754722824404% | 10 out of 9746 0.102606197414324% | 0.0032 | 2.41 | 1 out of 145 0.689655172413793% | 2 out of 264 0.757575757575758% | 0.7978 | 0.91 |
| Anthocyanin biosynthesis | ko00942 | 12 out of 3229 0.371632084236606% | 18 out of 9746 0.184691155345783% | 0.0037 | 2.01 | 1 out of 145 0.689655172413793% | 2 out of 264 0.757575757575758% | 0.7978 | 0.91 |
| Inositol phosphate metabolism | ko00562 | 56 out of 3229 1.73428305977083% | 130 out of 9746 1.33388056638621% | 0.0109 | 1.30 | 1 out of 145 0.689655172413793% | 4 out of 264 1.51515151515152% | 0.9599 | 0.46 |
| Pentose and glucuronate interconversions | ko00040 | 89 out of 3229 2.75627129142149% | 220 out of 9746 2.25733634311512% | 0.0127 | 1.22 | 1 out of 145 0.689655172413793% | 5 out of 264 1.89393939393939% | 0.9822 | 0.36 |
| Glycine, serine and threonine metabolism | ko00260 | 45 out of 3229 1.39362031588727% | 104 out of 9746 1.06710445310897% | 0.0192 | 1.31 | 5 out of 145 3.44827586206897% | 7 out of 264 2.65151515151515% | 0.3123 | 1.30 |
| Isoquinoline alkaloid biosynthesis | ko00950 | 22 out of 3229 0.681325487767111% | 45 out of 9746 0.461727888364457% | 0.0202 | 1.48 | 2 out of 145 1.37931034482759% | 4 out of 264 1.51515151515152% | 0.7592 | 0.91 |
| Flavonoid biosynthesis | ko00941 | 48 out of 3229 1.48652833694642% | 113 out of 9746 1.15945003078186% | 0.0230 | 1.28 | 14 out of 145 9.6551724137931% | 18 out of 264 6.81818181818182% | 0.0354 | 1.42 |
| Steroid biosynthesis | ko00100 | 20 out of 3229 0.61938680706101% | 41 out of 9746 0.420685409398728% | 0.0269 | 1.47 | 1 out of 145 0.689655172413793% | 1 out of 264 0.378787878787879% | 0.5492 | 1.82 |
| Cutin, suberine and wax biosynthesis | ko00073 | 29 out of 3229 0.898110870238464% | 65 out of 9746 0.666940283193105% | 0.0349 | 1.35 | 3 out of 145 2.06896551724138% | 3 out of 264 1.13636363636364% | 0.1641 | 1.82 |
| Nicotinate and nicotinamide metabolism | ko00760 | 16 out of 3229 0.495509445648808% | 32 out of 9746 0.328339831725836% | 0.0354 | 1.51 | 2 out of 145 1.37931034482759% | 4 out of 264 1.51515151515152% | 0.7592 | 0.91 |
| Folate biosynthesis | ko00790 | 19 out of 3229 0.588417466707959% | 40 out of 9746 0.410424789657295% | 0.0412 | 1.43 | 1 out of 145 0.689655172413793% | 2 out of 264 0.757575757575758% | 0.7978 | 0.91 |
| Phenylalanine, tyrosine and tryptophan biosynthesis | ko00400 | 33 out of 3229 1.02198823165067% | 77 out of 9746 0.790067720090293% | 0.0467 | 1.29 | 3 out of 145 2.06896551724138% | 4 out of 264 1.51515151515152% | 0.3886 | 1.37 |
| Tropane, piperidine and pyridine alkaloid biosynthesis | ko00960 | 18 out of 3229 0.557448126354909% | 39 out of 9746 0.400164169915863% | 0.0618 | 1.39 | 4 out of 145 2.75862068965517% | 6 out of 264 2.27272727272727% | 0.4390 | 1.21 |
| alpha-Linolenic acid metabolism | ko00592 | 37 out of 3229 1.14586559306287% | 90 out of 9746 0.923455776728914% | 0.0681 | 1.24 | 3 out of 145 2.06896551724138% | 6 out of 264 2.27272727272727% | 0.7459 | 0.91 |
| Purine metabolism | ko00230 | 58 out of 3229 1.79622174047693% | 150 out of 9746 1.53909296121486% | 0.0874 | 1.17 | 10 out of 145 6.89655172413793% | 12 out of 264 4.54545454545455% | 0.0383 | 1.52 |
| Lysine biosynthesis | ko00300 | 7 out of 3229 0.216785382471353% | 13 out of 9746 0.133388056638621% | 0.1005 | 1.63 | 3 out of 145 2.06896551724138% | 3 out of 264 1.13636363636364% | 0.1641 | 1.82 |
| Starch and sucrose metabolism | ko00500 | 121 out of 3229 3.74729018271911% | 333 out of 9746 3.41678637389698% | 0.1146 | 1.10 | 4 out of 145 2.75862068965517% | 7 out of 264 2.65151515151515% | 0.6079 | 1.04 |
| Biotin metabolism | ko00780 | 10 out of 3229 0.309693403530505% | 21 out of 9746 0.21547301457008% | 0.1204 | 1.44 | 2 out of 145 1.37931034482759% | 2 out of 264 0.757575757575758% | 0.3007 | 1.82 |
| Linoleic acid metabolism | ko00591 | 11 out of 3229 0.340662743883555% | 24 out of 9746 0.246254873794377% | 0.1350 | 1.38 | 3 out of 145 2.06896551724138% | 4 out of 264 1.51515151515152% | 0.3886 | 1.37 |
| Terpenoid backbone biosynthesis | ko00900 | 33 out of 3229 1.02198823165067% | 84 out of 9746 0.86189205828032% | 0.1389 | 1.19 | 1 out of 145 0.689655172413793% | 2 out of 264 0.757575757575758% | 0.7978 | 0.91 |
| Carbon fixation in photosynthetic organisms | ko00710 | 44 out of 3229 1.36265097553422% | 115 out of 9746 1.17997127026472% | 0.1413 | 1.15 | 4 out of 145 2.75862068965517% | 4 out of 264 1.51515151515152% | 0.0893 | 1.82 |
| Sulfur relay system | ko04122 | 7 out of 3229 0.216785382471353% | 14 out of 9746 0.143648676380053% | 0.1454 | 1.51 | 1 out of 145 0.689655172413793% | 1 out of 264 0.378787878787879% | 0.5492 | 1.82 |
| Alanine, aspartate and glutamate metabolism | ko00250 | 22 out of 3229 0.681325487767111% | 54 out of 9746 0.554073466037349% | 0.1479 | 1.23 | 5 out of 145 3.44827586206897% | 6 out of 264 2.27272727272727% | 0.1597 | 1.52 |
| Cyanoamino acid metabolism | ko00460 | 31 out of 3229 0.960049550944565% | 79 out of 9746 0.810588959573158% | 0.1497 | 1.18 | 6 out of 145 4.13793103448276% | 7 out of 264 2.65151515151515% | 0.0986 | 1.56 |
| Pyrimidine metabolism | ko00240 | 37 out of 3229 1.14586559306287% | 97 out of 9746 0.995280114918941% | 0.1718 | 1.15 | 6 out of 145 4.13793103448276% | 8 out of 264 3.03030303030303% | 0.2150 | 1.37 |
| Flavone and flavonol biosynthesis | ko00944 | 12 out of 3229 0.371632084236606% | 28 out of 9746 0.287297352760107% | 0.1845 | 1.29 | 10 out of 145 6.89655172413793% | 12 out of 264 4.54545454545455% | 0.0383 | 1.52 |
| Thiamine metabolism | ko00730 | 16 out of 3229 0.495509445648808% | 39 out of 9746 0.400164169915863% | 0.1885 | 1.24 | 1 out of 145 0.689655172413793% | 1 out of 264 0.378787878787879% | 0.5492 | 1.82 |
| Taurine and hypotaurine metabolism | ko00430 | 3 out of 3229 0.0929080210591514% | 5 out of 9746 0.0513030987071619% | 0.2068 | 1.81 | 3 out of 145 2.06896551724138% | 4 out of 264 1.51515151515152% | 0.3886 | 1.37 |
| Tyrosine metabolism | ko00350 | 34 out of 3229 1.05295757200372% | 91 out of 9746 0.933716396470347% | 0.2252 | 1.13 | 2 out of 145 1.37931034482759% | 6 out of 264 2.27272727272727% | 0.9325 | 0.61 |
| Fatty acid biosynthesis | ko00061 | 28 out of 3229 0.867141529885413% | 74 out of 9746 0.759285860865996% | 0.2280 | 1.14 | 4 out of 145 2.75862068965517% | 4 out of 264 1.51515151515152% | 0.0893 | 1.82 |
| Phenylalanine metabolism | ko00360 | 28 out of 3229 0.867141529885413% | 74 out of 9746 0.759285860865996% | 0.2280 | 1.14 | 5 out of 145 3.44827586206897% | 7 out of 264 2.65151515151515% | 0.3123 | 1.30 |
| Sulfur metabolism | ko00920 | 17 out of 3229 0.526478786001858% | 43 out of 9746 0.441206648881592% | 0.2297 | 1.19 | 3 out of 145 2.06896551724138% | 5 out of 264 1.89393939393939% | 0.5924 | 1.09 |
| Fructose and mannose metabolism | ko00051 | 36 out of 3229 1.11489625270982% | 97 out of 9746 0.995280114918941% | 0.2314 | 1.12 | 2 out of 145 1.37931034482759% | 7 out of 264 2.65151515151515% | 0.9658 | 0.52 |
| Biosynthesis of amino acids | ko01230 | 122 out of 3229 3.77825952307216% | 348 out of 9746 3.57069567001847% | 0.2351 | 1.06 | 18 out of 145 12.4137931034483% | 21 out of 264 7.95454545454545% | 0.0022 | 1.56 |
| 2-Oxocarboxylic acid metabolism | ko01210 | 25 out of 3229 0.774233508826262% | 66 out of 9746 0.677200902934537% | 0.2425 | 1.14 | 9 out of 145 6.20689655172414% | 11 out of 264 4.16666666666667% | 0.0606 | 1.49 |
| Glutathione metabolism | ko00480 | 45 out of 3229 1.39362031588727% | 125 out of 9746 1.28257746767905% | 0.2753 | 1.09 | 1 out of 145 0.689655172413793% | 1 out of 264 0.378787878787879% | 0.5492 | 1.82 |
| Fatty acid elongation | ko00062 | 14 out of 3229 0.433570764942707% | 36 out of 9746 0.369382310691566% | 0.2841 | 1.17 | 1 out of 145 0.689655172413793% | 1 out of 264 0.378787878787879% | 0.5492 | 1.82 |
| Cysteine and methionine metabolism | ko00270 | 50 out of 3229 1.54846701765252% | 140 out of 9746 1.43648676380053% | 0.2843 | 1.08 | 6 out of 145 4.13793103448276% | 8 out of 264 3.03030303030303% | 0.2150 | 1.37 |
| Indole alkaloid biosynthesis | ko00901 | 3 out of 3229 0.0929080210591514% | 6 out of 9746 0.0615637184485943% | 0.3156 | 1.51 | 1 out of 145 0.689655172413793% | 1 out of 264 0.378787878787879% | 0.5492 | 1.82 |
| beta-Alanine metabolism | ko00410 | 24 out of 3229 0.743264168473212% | 66 out of 9746 0.677200902934537% | 0.3299 | 1.10 | 3 out of 145 2.06896551724138% | 4 out of 264 1.51515151515152% | 0.3886 | 1.37 |
| Carbon metabolism | ko01200 | 139 out of 3229 4.30473830907402% | 407 out of 9746 4.17607223476298% | 0.3454 | 1.03 | 5 out of 145 3.44827586206897% | 9 out of 264 3.40909090909091% | 0.6214 | 1.01 |
| Vitamin B6 metabolism | ko00750 | 7 out of 3229 0.216785382471353% | 18 out of 9746 0.184691155345783% | 0.3843 | 1.17 | 2 out of 145 1.37931034482759% | 3 out of 264 1.13636363636364% | 0.5739 | 1.21 |
| Arginine biosynthesis | ko00220 | 13 out of 3229 0.402601424589656% | 37 out of 9746 0.379642930432998% | 0.4586 | 1.06 | 5 out of 145 3.44827586206897% | 6 out of 264 2.27272727272727% | 0.1597 | 1.52 |
| Lysine degradation | ko00310 | 25 out of 3229 0.774233508826262% | 73 out of 9746 0.749025241124564% | 0.4633 | 1.03 | 3 out of 145 2.06896551724138% | 6 out of 264 2.27272727272727% | 0.7459 | 0.91 |
| Tryptophan metabolism | ko00380 | 31 out of 3229 0.960049550944565% | 92 out of 9746 0.943977016211779% | 0.4934 | 1.02 | 1 out of 145 0.689655172413793% | 2 out of 264 0.757575757575758% | 0.7978 | 0.91 |
| Monobactam biosynthesis | ko00261 | 6 out of 3229 0.185816042118303% | 17 out of 9746 0.174430535604351% | 0.5153 | 1.07 | 5 out of 145 3.44827586206897% | 6 out of 264 2.27272727272727% | 0.1597 | 1.52 |
| Arginine and proline metabolism | ko00330 | 27 out of 3229 0.836172189532363% | 81 out of 9746 0.831110199056023% | 0.5265 | 1.01 | 4 out of 145 2.75862068965517% | 4 out of 264 1.51515151515152% | 0.0893 | 1.82 |
| Glucosinolate biosynthesis | ko00966 | 3 out of 3229 0.0929080210591514% | 8 out of 9746 0.0820849579314591% | 0.5269 | 1.13 | 6 out of 145 4.13793103448276% | 6 out of 264 2.27272727272727% | 0.0262 | 1.82 |
| Galactose metabolism | ko00052 | 52 out of 3229 1.61040569835863% | 157 out of 9746 1.61091729940488% | 0.5315 | 1.00 | 5 out of 145 3.44827586206897% | 14 out of 264 5.3030303030303% | 0.9610 | 0.65 |
| Fatty acid degradation | ko00071 | 31 out of 3229 0.960049550944565% | 95 out of 9746 0.974758875436076% | 0.5799 | 0.98 | 2 out of 145 1.37931034482759% | 2 out of 264 0.757575757575758% | 0.3007 | 1.82 |
| Isoflavonoid biosynthesis | ko00943 | 12 out of 3229 0.371632084236606% | 37 out of 9746 0.379642930432998% | 0.5973 | 0.98 | 6 out of 145 4.13793103448276% | 9 out of 264 3.40909090909091% | 0.3569 | 1.21 |
| Fatty acid metabolism | ko01212 | 34 out of 3229 1.05295757200372% | 107 out of 9746 1.09788631233326% | 0.6528 | 0.96 | 2 out of 145 1.37931034482759% | 2 out of 264 0.757575757575758% | 0.3007 | 1.82 |
| C5-Branched dibasic acid metabolism | ko00660 | 3 out of 3229 0.0929080210591514% | 10 out of 9746 0.102606197414324% | 0.6962 | 0.91 | 2 out of 145 1.37931034482759% | 3 out of 264 1.13636363636364% | 0.5739 | 1.21 |
| Valine, leucine and isoleucine degradation | ko00280 | 31 out of 3229 0.960049550944565% | 101 out of 9746 1.03632259388467% | 0.7331 | 0.93 | 3 out of 145 2.06896551724138% | 5 out of 264 1.89393939393939% | 0.5924 | 1.09 |
| Nitrogen metabolism | ko00910 | 13 out of 3229 0.402601424589656% | 45 out of 9746 0.461727888364457% | 0.7754 | 0.87 | 2 out of 145 1.37931034482759% | 2 out of 264 0.757575757575758% | 0.3007 | 1.82 |
| Betalain biosynthesis | ko00965 | 4 out of 3229 0.123877361412202% | 15 out of 9746 0.153909296121486% | 0.7862 | 0.80 | 2 out of 145 1.37931034482759% | 2 out of 264 0.757575757575758% | 0.3007 | 1.82 |
| Amino sugar and nucleotide sugar metabolism | ko00520 | 81 out of 3229 2.50851656859709% | 261 out of 9746 2.67802175251385% | 0.7863 | 0.94 | 2 out of 145 1.37931034482759% | 6 out of 264 2.27272727272727% | 0.9325 | 0.61 |
| Valine, leucine and isoleucine biosynthesis | ko00290 | 6 out of 3229 0.185816042118303% | 23 out of 9746 0.235994254052945% | 0.8257 | 0.79 | 4 out of 145 2.75862068965517% | 5 out of 264 1.89393939393939% | 0.2528 | 1.46 |
| Selenocompound metabolism | ko00450 | 6 out of 3229 0.185816042118303% | 23 out of 9746 0.235994254052945% | 0.8257 | 0.79 | 1 out of 145 0.689655172413793% | 1 out of 264 0.378787878787879% | 0.5492 | 1.82 |
| Glycolysis / Gluconeogenesis | ko00010 | 75 out of 3229 2.32270052647879% | 247 out of 9746 2.5343730761338% | 0.8424 | 0.92 | 3 out of 145 2.06896551724138% | 3 out of 264 1.13636363636364% | 0.1641 | 1.82 |
| Butanoate metabolism | ko00650 | 11 out of 3229 0.340662743883555% | 41 out of 9746 0.420685409398728% | 0.8478 | 0.81 | 1 out of 145 0.689655172413793% | 3 out of 264 1.13636363636364% | 0.9097 | 0.61 |
| Pantothenate and CoA biosynthesis | ko00770 | 14 out of 3229 0.433570764942707% | 52 out of 9746 0.533552226554484% | 0.8656 | 0.81 | 2 out of 145 1.37931034482759% | 2 out of 264 0.757575757575758% | 0.3007 | 1.82 |
| Biosynthesis of unsaturated fatty acids | ko01040 | 9 out of 3229 0.278724063177454% | 35 out of 9746 0.359121690950133% | 0.8687 | 0.78 | 3 out of 145 2.06896551724138% | 3 out of 264 1.13636363636364% | 0.1641 | 1.82 |
| Diterpenoid biosynthesis | ko00904 | 10 out of 3229 0.309693403530505% | 41 out of 9746 0.420685409398728% | 0.9159 | 0.74 | 1 out of 145 0.689655172413793% | 3 out of 264 1.13636363636364% | 0.9097 | 0.61 |
| Aminoacyl-tRNA biosynthesis | ko00970 | 34 out of 3229 1.05295757200372% | 123 out of 9746 1.26205622819618% | 0.9207 | 0.83 | 15 out of 145 10.3448275862069% | 16 out of 264 6.06060606060606% | 0.0007 | 1.71 |
| Glyoxylate and dicarboxylate metabolism | ko00630 | 38 out of 3229 1.17683493341592% | 137 out of 9746 1.40570490457624% | 0.9271 | 0.84 | 5 out of 145 3.44827586206897% | 8 out of 264 3.03030303030303% | 0.4745 | 1.14 |
| Citrate cycle (TCA cycle) | ko00020 | 21 out of 3229 0.65035614741406% | 81 out of 9746 0.831110199056023% | 0.9360 | 0.78 | 1 out of 145 0.689655172413793% | 2 out of 264 0.757575757575758% | 0.7978 | 0.91 |
| Ascorbate and aldarate metabolism | ko00053 | 32 out of 3229 0.991018891297615% | 121 out of 9746 1.24153498871332% | 0.9547 | 0.80 | 1 out of 145 0.689655172413793% | 3 out of 264 1.13636363636364% | 0.9097 | 0.61 |
| Pyruvate metabolism | ko00620 | 41 out of 3229 1.26974295447507% | 152 out of 9746 1.55961420069772% | 0.9585 | 0.81 | 1 out of 145 0.689655172413793% | 2 out of 264 0.757575757575758% | 0.7978 | 0.91 |
| Zeatin biosynthesis | ko00908 | 18 out of 3229 0.557448126354909% | 79 out of 9746 0.810588959573158% | 0.9838 | 0.69 | 1 out of 145 0.689655172413793% | 2 out of 264 0.757575757575758% | 0.7978 | 0.91 |
| Histidine metabolism | ko00340 | 9 out of 3229 0.278724063177454% | 50 out of 9746 0.513030987071619% | 0.9946 | 0.54 | 3 out of 145 2.06896551724138% | 4 out of 264 1.51515151515152% | 0.3886 | 1.37 |
| ABC transporters | ko02010 | 46 out of 3229 1.42458965624032% | 200 out of 9746 2.05212394828648% | 0.9994 | 0.69 | 26 out of 145 17.9310344827586% | 39 out of 264 14.7727272727273% | 0.0766 | 1.21 |
| Note:  Kegg_pathway: Designations of pathways (or subcategories) in the KEGG database. ko_id: Pathway IDs in the KEGG database. Gene_Cluter_frequency: The proportion of target functional genes in the differential gene set under the specific pathway. Gene_Genome_frequency: The proportion of target functional genes in the genomic background. Gene_P-value: The corrected p-value of DEGs obtained through Fisher's exact test. Gene_rich_factor: The ratio of pathway-annotated genes among DEGs to that among all genes. Metabolite_Cluter_frequency: The proportion of target functional metabolites in the differential metabolite set under the specific pathway. Metabolite_Genome_frequency: The proportion of target functional metabolites in the metabolic background. Metabolite_P-value: The corrected p-value of DAMs obtained through Fisher's exact test. Metabolite_rich_factor: The ratio of pathway-annotated metabolites among DAMs to that among all metabolites. | | | | | | | | | |

**Table S3 Statistical characteristics of DEGs/DAMs within enriched KEGG pathways of different groups**

| **LFI/LFO group** | | | | | | | | | | | | | |
| --- | --- | --- | --- | --- | --- | --- | --- | --- | --- | --- | --- | --- | --- |
| **ID-Gene** | **LFI01** | **LFI02** | **LFI03** | **LFO01** | **LFO02** | **LFO03** | **ID-Meta** | **LFI01** | **LFI02** | **LFI03** | **LFO01** | **LFO02** | **LFO03** |
| **Flavonoid biosynthesis (ko00941)** | | | | | | | | | | | | | |
| **Genes** | | | | | | | **Metabolites** | | | | | | |
| TRINITY_DN717_c1_g1 | 27.05 | 29.28 | 36.71 | 77.95 | 55.84 | 77.67 | Taxifolin | 39742946.69 | 44270816.76 | 45125342.91 | 6847326.709 | 6296053.437 | 8207357.823 |
| TRINITY_DN4827_c2_g1 | 21.12 | 22.02 | 21.94 | 846.14 | 884.33 | 754.61 | (+)-Gallocatechin | 994894 | 986698 | 929896.4961 | 10.00190286 | 10.00190286 | 10.00190286 |
| TRINITY_DN6380_c0_g1 | 26.71 | 25.67 | 25.66 | 4.29 | 5.57 | 4.49 | (-)-Epigallocatechin | 871959.0519 | 735525.3939 | 937399.8072 | 10.00190286 | 10.00190286 | 10.00190286 |
| TRINITY_DN294_c1_g1 | 5.32 | 6.68 | 7.52 | 28.09 | 28.65 | 24.66 | Naringenin Chalcone | 5319045.833 | 4929109.283 | 4534084.651 | 2434843.46 | 2555108.029 | 2546634.219 |
| TRINITY_DN2979_c0_g1 | 13.95 | 12.98 | 13.57 | 0 | 0 | 0 | Chrysin | 9042.093551 | 7822.508786 | 8828.695693 | 22928.29801 | 26205.60424 | 31225.13355 |
| TRINITY_DN2533_c1_g3 | 68.82 | 65.27 | 65.04 | 0.53 | 0.51 | 0.46 | Phloretin | 14716455.46 | 15321511.45 | 15146918.33 | 293750.5948 | 401199.3695 | 387807.6308 |
| TRINITY_DN2269_c2_g1 | 0.63 | 1.55 | 1.25 | 6.35 | 5.07 | 3.8 | Phlorizin | 9295138.056 | 10140109.51 | 9478532.19 | 505996.9568 | 569874.9857 | 561043.8839 |
| TRINITY_DN447_c0_g1 | 8.6 | 9.05 | 9.3 | 16.95 | 17.06 | 15.57 | Dihydromyricetin | 6676407.478 | 6324010.476 | 5682204.728 | 11102.37146 | 8655.450516 | 7551.473295 |
| TRINITY_DN6019_c0_g2 | 4.38 | 5.53 | 3.41 | 0.26 | 0.59 | 0.12 | Pinocembrin | 49350.80535 | 42050 | 40946.57276 | 174431.7808 | 219446.8748 | 242702.4257 |
| TRINITY_DN4149_c0_g1 | 9 | 8.35 | 9.52 | 2.44 | 1.95 | 1.83 | P-Coumaroyl Quinic Acid | 267425139.3 | 256084522.1 | 258625558.2 | 107461412.6 | 109533966.4 | 111938821.6 |
| TRINITY_DN3240_c0_g2 | 48.26 | 50.24 | 52.34 | 1.06 | 1.57 | 0.97 | Kaempferol | 1998156.42 | 1803933.414 | 2077306.839 | 947430.8676 | 1294838.715 | 1235579.254 |
| TRINITY_DN13667_c0_g1 | 0.47 | 0.17 | 0.47 | 4.07 | 4.35 | 3.4 | Luteolin | 4624872.723 | 5485243.401 | 5970540.318 | 1781848.075 | 2284845.754 | 2202946.805 |
| TRINITY_DN13053_c0_g2 | 111.52 | 111.04 | 104.92 | 7.32 | 5.04 | 6.55 | (+)-Taxifolin | 49906174.51 | 51945566.52 | 46771642.89 | 8010241.003 | 9084673.413 | 9412389.979 |
| TRINITY_DN54451_c0_g1 | 117.36 | 119.37 | 113.42 | 0.21 | 0.18 | 0.3 | Apigenin | 2341021.854 | 2656356.251 | 3062345.246 | 1274474.527 | 1434289.438 | 1458124.168 |
| TRINITY_DN7389_c0_g1 | 25.33 | 26.94 | 28.71 | 1420.75 | 1403.84 | 1291.21 | Myricetin | 29674325.1 | 26572490.82 | 27994887.12 | 52061.76271 | 46411.18322 | 46161.95568 |
| TRINITY_DN6292_c5_g1 | 3.38 | 2.79 | 2.45 | 9.14 | 9.49 | 10.61 | Tricetin | 4589329.014 | 4660291.479 | 5249036.861 | 10.00190286 | 10.00190286 | 10.00190286 |
| TRINITY_DN3454_c1_g1 | 1.61 | 1.34 | 1.39 | 0.03 | 0.08 | 0 | Dihydrokaempferol | 37342213.08 | 41325354.58 | 44211896.63 | 8503543.816 | 8712182.696 | 8075430.831 |
| TRINITY_DN1429_c2_g1 | 52.88 | 53.3 | 49.54 | 373.38 | 361.29 | 320.04 |  |  |  |  |  |  |  |
| TRINITY_DN2821_c0_g1 | 8.9 | 9.57 | 7.94 | 0 | 0.14 | 0.11 |  |  |  |  |  |  |  |
| TRINITY_DN1238_c1_g1 | 3.51 | 2.44 | 2.23 | 8.83 | 9.96 | 5.38 |  |  |  |  |  |  |  |
| TRINITY_DN3710_c0_g1 | 67.37 | 72.45 | 68.34 | 145.78 | 149.26 | 130.79 |  |  |  |  |  |  |  |
| TRINITY_DN5157_c0_g1 | 15.63 | 14.06 | 14.66 | 3.57 | 2.42 | 2.12 |  |  |  |  |  |  |  |
| TRINITY_DN9141_c0_g4 | 7.03 | 7.57 | 8.03 | 61.93 | 62.35 | 58.67 |  |  |  |  |  |  |  |
| TRINITY_DN4871_c0_g2 | 0.43 | 0.26 | 0.46 | 1.49 | 2.29 | 1.41 |  |  |  |  |  |  |  |
| TRINITY_DN670_c0_g1 | 5.07 | 5.62 | 5.19 | 11.82 | 7.88 | 11.01 |  |  |  |  |  |  |  |
| TRINITY_DN26153_c0_g1 | 1.99 | 1.57 | 1.55 | 0.2 | 0.75 | 0.06 |  |  |  |  |  |  |  |
| TRINITY_DN1429_c0_g1 | 20.96 | 23.88 | 23.72 | 128.14 | 111.54 | 110.27 |  |  |  |  |  |  |  |
| TRINITY_DN2887_c4_g1 | 1.49 | 1.83 | 1.33 | 4.29 | 4.6 | 4.54 |  |  |  |  |  |  |  |
| TRINITY_DN3834_c0_g2 | 3.64 | 3.53 | 3.12 | 0.25 | 0.92 | 0.1 |  |  |  |  |  |  |  |
| TRINITY_DN751_c1_g2 | 30.08 | 29.85 | 30.68 | 0.67 | 0.62 | 0.04 |  |  |  |  |  |  |  |
| TRINITY_DN4578_c0_g1 | 16.14 | 17.23 | 16.34 | 31.67 | 32.78 | 29.4 |  |  |  |  |  |  |  |
| TRINITY_DN111_c0_g3 | 12.1 | 12.62 | 13.58 | 3.24 | 3.55 | 3.2 |  |  |  |  |  |  |  |
| TRINITY_DN1192_c0_g1 | 8.85 | 12.79 | 9.49 | 0.36 | 0.23 | 0.31 |  |  |  |  |  |  |  |
| TRINITY_DN11108_c0_g1 | 26.37 | 27.8 | 27.23 | 219.15 | 220.7 | 191.98 |  |  |  |  |  |  |  |
| TRINITY_DN3179_c0_g2 | 9.6 | 8.43 | 10.95 | 3.55 | 1.83 | 4.17 |  |  |  |  |  |  |  |
| TRINITY_DN208_c1_g2 | 32.43 | 32.89 | 32.24 | 2.6 | 2.32 | 2.35 |  |  |  |  |  |  |  |
| TRINITY_DN19920_c1_g1 | 359.48 | 371.37 | 362.46 | 3.3 | 2.83 | 2.08 |  |  |  |  |  |  |  |
| TRINITY_DN24276_c0_g1 | 85.37 | 83.15 | 82.66 | 29.21 | 32.48 | 28.13 |  |  |  |  |  |  |  |
| TRINITY_DN10806_c0_g3 | 5.75 | 5.42 | 4.78 | 2.01 | 2.91 | 1.44 |  |  |  |  |  |  |  |
| TRINITY_DN4871_c0_g3 | 63.93 | 62.77 | 63.98 | 525.68 | 507.36 | 468.77 |  |  |  |  |  |  |  |
| TRINITY_DN5948_c0_g1 | 4.36 | 4.04 | 2.94 | 14.87 | 14.59 | 12.16 |  |  |  |  |  |  |  |
| TRINITY_DN3240_c0_g1 | 6.03 | 5.2 | 4.42 | 336.11 | 338.58 | 292.81 |  |  |  |  |  |  |  |
| TRINITY_DN2533_c1_g4 | 9.4 | 9.33 | 8.31 | 24.81 | 23.12 | 21.96 |  |  |  |  |  |  |  |
| TRINITY_DN4240_c0_g1 | 1529.38 | 1514.01 | 1539.71 | 57.16 | 53.99 | 53.6 |  |  |  |  |  |  |  |
| TRINITY_DN4635_c0_g1 | 6.55 | 6.68 | 6.12 | 1.49 | 3.04 | 1.71 |  |  |  |  |  |  |  |
| TRINITY_DN648_c0_g1 | 36.16 | 33.66 | 35.68 | 14.74 | 13.59 | 12.83 |  |  |  |  |  |  |  |
| TRINITY_DN24276_c0_g2 | 13.23 | 15.03 | 9.78 | 5.24 | 2.02 | 3.53 |  |  |  |  |  |  |  |
| TRINITY_DN17224_c1_g1 | 9.18 | 8.42 | 10.28 | 0.26 | 0.81 | 0.39 |  |  |  |  |  |  |  |
| TRINITY_DN277_c0_g1 | 51.95 | 56.08 | 54.55 | 3816.24 | 3601.21 | 3270.55 |  |  |  |  |  |  |  |
| TRINITY_DN5199_c0_g1 | 108.27 | 104.7 | 104.31 | 12.74 | 12.23 | 14.92 |  |  |  |  |  |  |  |
| **Biosynthesis of amino acids (ko01230)** | | | | | | | | | | | | | |
| **Genes** | | | | | | | **Metabolites** | | | | | | |
| TRINITY_DN6094_c0_g2 | 240.55 | 253.9 | 253.92 | 1807.99 | 1749.68 | 1651.57 | L-Glutamine | 11680441.31 | 11317303.2 | 11747915.13 | 8505814.528 | 8023701.513 | 7450598.618 |
| TRINITY_DN34949_c0_g1 | 1.79 | 2.78 | 2.3 | 0.67 | 0.07 | 0.79 | L-Homoserine | 1902787.489 | 1694921.465 | 1872179.17 | 3161073.96 | 2862602.175 | 2931724.281 |
| TRINITY_DN9382_c0_g2 | 11.34 | 10.54 | 10.8 | 23.13 | 24.27 | 19.43 | L-Tyrosine | 4324006.032 | 4826614.316 | 5133722.451 | 1726808.003 | 1699870.9 | 1652112.486 |
| TRINITY_DN24479_c0_g1 | 2.74 | 2.84 | 2.5 | 0 | 0 | 0 | L-Valine | 9719811.901 | 9611411.058 | 9646195.23 | 6716318.141 | 6531363.095 | 6378092.297 |
| TRINITY_DN135_c5_g1 | 8.96 | 8.46 | 7.46 | 25.79 | 25.04 | 23.09 | L-Serine | 1654326.186 | 1506170.737 | 1482295.541 | 2812208.811 | 2657323.226 | 2703792.312 |
| TRINITY_DN6152_c0_g1 | 69.84 | 65.36 | 67.05 | 204.6 | 195.19 | 184.22 | L-Lysine | 20743446.49 | 22241406.11 | 21385437.53 | 14653581.93 | 13953047.14 | 12603661.09 |
| TRINITY_DN32687_c0_g1 | 0.49 | 2.4 | 2.87 | 10.92 | 11.78 | 11.58 | L-Proline | 127072456.7 | 140270210.6 | 135944277.1 | 11738451.86 | 11099983.41 | 10595346.28 |
| TRINITY_DN1739_c0_g2 | 10.9 | 12.92 | 13.2 | 23.29 | 20.57 | 17.51 | L-Threonine | 13522912.24 | 15579512.52 | 13910743.14 | 23149475.34 | 23941689.56 | 22467425.97 |
| TRINITY_DN2077_c0_g1 | 13.68 | 13.67 | 12.91 | 24.13 | 22.63 | 19.26 | L-Arginine | 71460129.66 | 78838035.58 | 82213417.76 | 8342639.814 | 6342085.205 | 5169170.022 |
| TRINITY_DN1435_c0_g3 | 14.93 | 14.86 | 14.17 | 1.68 | 7.83 | 2.56 | L-Citrulline | 2835078.539 | 2721143.915 | 3171799.27 | 1491562.691 | 1176153.674 | 896357.4662 |
| TRINITY_DN1524_c0_g1 | 172.84 | 165.43 | 166.25 | 0.08 | 0.12 | 0.33 | L-Alanine | 304275.8163 | 331727.9633 | 270310.1267 | 385545.8415 | 426981.2958 | 417619.4246 |
| TRINITY_DN3812_c0_g1 | 52.89 | 48.33 | 49.24 | 294.34 | 275.41 | 261.22 | L-Asparagine | 3096130.431 | 3183144.978 | 3165125.468 | 583326.7419 | 535342.8801 | 476762.2368 |
| TRINITY_DN1237_c1_g1 | 21.05 | 22.8 | 21.45 | 103.2 | 102.96 | 90.06 | L-Phenylalanine | 194332242.3 | 196792274.2 | 213878931 | 379723411.8 | 363023217.6 | 361591053.3 |
| TRINITY_DN771_c0_g2 | 32.38 | 30.69 | 33.5 | 106.02 | 98.26 | 91.05 | N-Acetyl-L-Glutamic Acid | 58027.85673 | 50416.34663 | 50793.03224 | 102950.6668 | 97625.07792 | 100420.2571 |
| TRINITY_DN6687_c0_g1 | 4.53 | 4.4 | 4 | 1.55 | 1.46 | 1.64 | Shikimic Acid | 27117153.06 | 26599081.35 | 27643834.59 | 4864605.561 | 4703724.356 | 4775823.23 |
| TRINITY_DN90_c1_g2 | 21.14 | 20.29 | 20.45 | 493.12 | 472.49 | 424.14 | L-Glutamic Acid | 15267530.52 | 14466275.86 | 15055842.27 | 7068507.123 | 6988421.439 | 7123276.088 |
| TRINITY_DN3120_c0_g1 | 1.52 | 2.68 | 1.26 | 1.99 | 3.18 | 2.73 | 2-Dehydro-3-deoxy-D-arabino-heptonate 7-phosphate | 2665.486843 | 2890.568304 | 2882.533258 | 913.9662213 | 1064.613293 | 1022.645934 |
| TRINITY_DN2725_c0_g1 | 4.23 | 4.72 | 3.49 | 41.37 | 25.83 | 29 |  |  |  |  |  |  |  |
| TRINITY_DN37501_c0_g1 | 1.26 | 2.16 | 2.07 | 0.33 | 0.92 | 0.31 |  |  |  |  |  |  |  |
| TRINITY_DN3939_c1_g2 | 7.69 | 7.9 | 8.11 | 2.9 | 2.54 | 2.52 |  |  |  |  |  |  |  |
| TRINITY_DN8039_c0_g1 | 22.1 | 25.65 | 22.21 | 47.62 | 42.24 | 40.08 |  |  |  |  |  |  |  |
| TRINITY_DN1335_c0_g1 | 105.14 | 104.62 | 109.38 | 33.45 | 27.53 | 31.61 |  |  |  |  |  |  |  |
| TRINITY_DN9007_c0_g1 | 1.22 | 1.1 | 1.25 | 24.32 | 22.59 | 22.56 |  |  |  |  |  |  |  |
| TRINITY_DN1115_c1_g1 | 4.41 | 7.57 | 3.91 | 22.82 | 18.38 | 20.17 |  |  |  |  |  |  |  |
| TRINITY_DN432_c0_g1 | 2.45 | 2.52 | 2.45 | 5.71 | 5.09 | 4.4 |  |  |  |  |  |  |  |
| TRINITY_DN8800_c0_g1 | 41.7 | 39.01 | 37.72 | 0.3 | 0.61 | 0.64 |  |  |  |  |  |  |  |
| TRINITY_DN4044_c0_g1 | 27.59 | 24.91 | 24.97 | 4.5 | 5.8 | 4.39 |  |  |  |  |  |  |  |
| TRINITY_DN62_c1_g2 | 4.83 | 5.49 | 4.83 | 9.37 | 7.58 | 8.12 |  |  |  |  |  |  |  |
| TRINITY_DN1785_c3_g1 | 33.35 | 37.52 | 38.7 | 8.51 | 8.94 | 10.37 |  |  |  |  |  |  |  |
| TRINITY_DN3939_c1_g1 | 6.41 | 4.94 | 7.03 | 2.86 | 2.43 | 0.87 |  |  |  |  |  |  |  |
| TRINITY_DN164_c1_g4 | 3.94 | 3.54 | 4.61 | 0 | 0 | 0 |  |  |  |  |  |  |  |
| TRINITY_DN4793_c1_g1 | 254.39 | 250.64 | 245.06 | 81.74 | 78.21 | 74.25 |  |  |  |  |  |  |  |
| TRINITY_DN13464_c0_g2 | 23.57 | 22.36 | 23.32 | 51.66 | 44.26 | 37.28 |  |  |  |  |  |  |  |
| TRINITY_DN620_c0_g3 | 10.48 | 10.06 | 6.95 | 0.49 | 0.26 | 0.23 |  |  |  |  |  |  |  |
| TRINITY_DN43347_c0_g1 | 19.9 | 17.54 | 17.14 | 75.24 | 72.8 | 67.42 |  |  |  |  |  |  |  |
| TRINITY_DN25060_c0_g2 | 14.2 | 13.62 | 13.68 | 2.41 | 2.49 | 1.6 |  |  |  |  |  |  |  |
| TRINITY_DN6717_c0_g1 | 9.32 | 9.87 | 9.81 | 0.08 | 0.05 | 0.08 |  |  |  |  |  |  |  |
| TRINITY_DN1583_c0_g1 | 75.04 | 73.56 | 68.64 | 3.34 | 8.25 | 8.01 |  |  |  |  |  |  |  |
| TRINITY_DN5293_c0_g1 | 31.88 | 30.11 | 28.92 | 0.15 | 0.19 | 0.4 |  |  |  |  |  |  |  |
| TRINITY_DN8752_c0_g1 | 17.04 | 16.89 | 15.5 | 59.81 | 52.13 | 60.9 |  |  |  |  |  |  |  |
| TRINITY_DN8719_c1_g1 | 84.17 | 84.2 | 82.39 | 171.46 | 170.62 | 157.67 |  |  |  |  |  |  |  |
| TRINITY_DN19220_c0_g2 | 12.15 | 12.92 | 13.77 | 2.42 | 2.67 | 1.73 |  |  |  |  |  |  |  |
| TRINITY_DN19453_c0_g1 | 19.91 | 21.37 | 24.35 | 0 | 0 | 0 |  |  |  |  |  |  |  |
| TRINITY_DN7771_c0_g1 | 1.83 | 1.46 | 1.57 | 4.32 | 2.78 | 2.35 |  |  |  |  |  |  |  |
| TRINITY_DN6064_c0_g1 | 44.59 | 43.82 | 44.78 | 78.87 | 79.99 | 67.94 |  |  |  |  |  |  |  |
| TRINITY_DN3130_c1_g2 | 1.58 | 1.88 | 1.52 | 0.63 | 0.16 | 0.11 |  |  |  |  |  |  |  |
| TRINITY_DN14190_c0_g3 | 93.35 | 88.29 | 83.54 | 4.68 | 5.84 | 6.48 |  |  |  |  |  |  |  |
| TRINITY_DN4394_c0_g1 | 3.15 | 2.59 | 3.1 | 7.59 | 4.58 | 5.19 |  |  |  |  |  |  |  |
| TRINITY_DN1134_c0_g2 | 1.37 | 1.27 | 0.98 | 0.45 | 0.05 | 0.21 |  |  |  |  |  |  |  |
| TRINITY_DN1435_c0_g4 | 12.68 | 12.79 | 12.48 | 31.62 | 28.31 | 24.27 |  |  |  |  |  |  |  |
| TRINITY_DN2000_c0_g1 | 6.09 | 4.77 | 4.29 | 1.41 | 1.44 | 1.15 |  |  |  |  |  |  |  |
| TRINITY_DN4507_c0_g1 | 10.91 | 7.53 | 9.12 | 23.68 | 20.71 | 17.77 |  |  |  |  |  |  |  |
| TRINITY_DN1263_c2_g1 | 1280.17 | 1185.15 | 1214.14 | 56.27 | 50.77 | 49.22 |  |  |  |  |  |  |  |
| TRINITY_DN11011_c0_g1 | 2.14 | 1.85 | 1.19 | 5.62 | 6.43 | 5.97 |  |  |  |  |  |  |  |
| TRINITY_DN5865_c1_g2 | 57.76 | 61.98 | 57.1 | 0.76 | 0.41 | 0.16 |  |  |  |  |  |  |  |
| TRINITY_DN30247_c0_g3 | 107.68 | 99.22 | 102.61 | 259.56 | 257.28 | 241.12 |  |  |  |  |  |  |  |
| TRINITY_DN995_c1_g2 | 0.36 | 1.15 | 0.51 | 1.45 | 1.59 | 1.83 |  |  |  |  |  |  |  |
| TRINITY_DN73_c0_g2 | 0.98 | 1.53 | 1.24 | 1.93 | 2.68 | 2.41 |  |  |  |  |  |  |  |
| TRINITY_DN1056_c0_g1 | 4.85 | 4.12 | 3.73 | 0.8 | 1.57 | 2.15 |  |  |  |  |  |  |  |
| TRINITY_DN11285_c0_g1 | 2.81 | 2.4 | 1.81 | 5.58 | 7.88 | 5.78 |  |  |  |  |  |  |  |
| TRINITY_DN12722_c0_g1 | 7.7 | 7.65 | 7.99 | 0.08 | 0.1 | 0.14 |  |  |  |  |  |  |  |
| TRINITY_DN620_c0_g4 | 4.88 | 4.11 | 5.19 | 19.12 | 18.58 | 16.64 |  |  |  |  |  |  |  |
| TRINITY_DN6590_c0_g1 | 11.92 | 10.83 | 10.12 | 28.83 | 24.62 | 24.3 |  |  |  |  |  |  |  |
| TRINITY_DN1853_c0_g1 | 65.45 | 63.15 | 63.57 | 189.84 | 179.13 | 162.2 |  |  |  |  |  |  |  |
| TRINITY_DN6933_c0_g1 | 9.82 | 13.46 | 8.17 | 39.41 | 38.37 | 30.74 |  |  |  |  |  |  |  |
| TRINITY_DN1583_c1_g1 | 6.42 | 6.84 | 6.41 | 20.25 | 20.01 | 15.59 |  |  |  |  |  |  |  |
| TRINITY_DN7295_c0_g1 | 0.57 | 0.46 | 0.08 | 1.33 | 2.72 | 3.13 |  |  |  |  |  |  |  |
| TRINITY_DN16406_c0_g1 | 13.12 | 13.32 | 13.42 | 1.2 | 1.17 | 1.5 |  |  |  |  |  |  |  |
| TRINITY_DN1938_c0_g1 | 18.85 | 16.9 | 21.37 | 35.49 | 38.41 | 31 |  |  |  |  |  |  |  |
| TRINITY_DN8111_c0_g1 | 12.73 | 14.97 | 12.84 | 4.15 | 4.75 | 4.41 |  |  |  |  |  |  |  |
| TRINITY_DN3303_c0_g1 | 1.27 | 1.94 | 1.27 | 13.15 | 11.92 | 12.2 |  |  |  |  |  |  |  |
| TRINITY_DN1050_c1_g1 | 134.49 | 112 | 112.49 | 537.06 | 569.31 | 489.63 |  |  |  |  |  |  |  |
| TRINITY_DN1513_c0_g1 | 10.87 | 9.15 | 8.99 | 3.73 | 3.98 | 3.11 |  |  |  |  |  |  |  |
| TRINITY_DN1483_c0_g2 | 138.61 | 137.97 | 137.92 | 240.6 | 224.23 | 218.2 |  |  |  |  |  |  |  |
| TRINITY_DN339_c0_g1 | 54.13 | 54.71 | 55.03 | 160.28 | 151.73 | 141.5 |  |  |  |  |  |  |  |
| TRINITY_DN982_c0_g1 | 5.6 | 4.8 | 4.92 | 1.99 | 1.23 | 1.51 |  |  |  |  |  |  |  |
| TRINITY_DN20811_c0_g1 | 19.47 | 18.59 | 18.95 | 48.65 | 54.8 | 44.98 |  |  |  |  |  |  |  |
| TRINITY_DN3099_c0_g2 | 111.74 | 106.51 | 107.25 | 252.17 | 236.63 | 218.23 |  |  |  |  |  |  |  |
| TRINITY_DN7382_c0_g1 | 4.17 | 4.31 | 5.1 | 11.54 | 10.23 | 7.12 |  |  |  |  |  |  |  |
| TRINITY_DN5127_c0_g1 | 3.47 | 4.38 | 3.3 | 0.24 | 1.12 | 0.26 |  |  |  |  |  |  |  |
| TRINITY_DN10502_c0_g1 | 1.07 | 0.94 | 0.73 | 0.05 | 0.03 | 0.06 |  |  |  |  |  |  |  |
| TRINITY_DN11139_c0_g1 | 6.17 | 3.45 | 4.77 | 0.27 | 0.16 | 0.86 |  |  |  |  |  |  |  |
| TRINITY_DN2939_c0_g1 | 17.26 | 16.19 | 13.58 | 1.98 | 1.67 | 2.27 |  |  |  |  |  |  |  |
| TRINITY_DN4144_c0_g1 | 9.95 | 10.21 | 12.78 | 21.65 | 23.61 | 21.43 |  |  |  |  |  |  |  |
| TRINITY_DN1653_c1_g1 | 8.74 | 10.07 | 9.68 | 16.96 | 18.31 | 14.81 |  |  |  |  |  |  |  |
| TRINITY_DN6717_c0_g2 | 43.72 | 41.07 | 41.23 | 13.73 | 8.65 | 8.55 |  |  |  |  |  |  |  |
| TRINITY_DN683_c0_g2 | 5.34 | 4.7 | 5.64 | 11.14 | 9.99 | 9.07 |  |  |  |  |  |  |  |
| TRINITY_DN17260_c0_g4 | 19.1 | 20.41 | 19.05 | 122.22 | 106.61 | 102.76 |  |  |  |  |  |  |  |
| TRINITY_DN607_c0_g1 | 34.16 | 35.57 | 34.61 | 102.01 | 94.75 | 88.69 |  |  |  |  |  |  |  |
| TRINITY_DN5575_c0_g1 | 57.8 | 55.29 | 54.43 | 7.31 | 7.13 | 7.66 |  |  |  |  |  |  |  |
| TRINITY_DN6754_c0_g4 | 12.01 | 11.44 | 11.62 | 20.11 | 20.05 | 19.58 |  |  |  |  |  |  |  |
| TRINITY_DN3563_c0_g1 | 307.28 | 282.68 | 281.73 | 92.27 | 89.36 | 83.48 |  |  |  |  |  |  |  |
| TRINITY_DN425_c0_g1 | 53.9 | 52.91 | 54.64 | 840.5 | 787.37 | 715.83 |  |  |  |  |  |  |  |
| TRINITY_DN5623_c0_g1 | 7.32 | 7.92 | 5.92 | 20.65 | 20.74 | 18.85 |  |  |  |  |  |  |  |
| TRINITY_DN10900_c0_g1 | 15.1 | 20.1 | 16.92 | 35.01 | 34.39 | 30.56 |  |  |  |  |  |  |  |
| TRINITY_DN1797_c5_g1 | 18.55 | 18.31 | 20.95 | 77.11 | 85.89 | 75.02 |  |  |  |  |  |  |  |
| TRINITY_DN2835_c0_g2 | 2.81 | 3.19 | 3 | 0.21 | 0.16 | 0 |  |  |  |  |  |  |  |
| TRINITY_DN1551_c1_g2 | 28.08 | 24.03 | 31.48 | 19.25 | 10.9 | 9.3 |  |  |  |  |  |  |  |
| TRINITY_DN3153_c0_g2 | 47.79 | 47.71 | 49.49 | 104.92 | 106.11 | 96.92 |  |  |  |  |  |  |  |
| TRINITY_DN54272_c0_g1 | 2.51 | 1.57 | 2 | 0.33 | 0.37 | 0.16 |  |  |  |  |  |  |  |
| TRINITY_DN127_c1_g3 | 17.13 | 15.85 | 16.58 | 37.8 | 32.76 | 28.8 |  |  |  |  |  |  |  |
| TRINITY_DN6869_c0_g1 | 104.43 | 104.84 | 108.71 | 17.66 | 16.43 | 17.46 |  |  |  |  |  |  |  |
| TRINITY_DN1484_c1_g1 | 45.01 | 39.81 | 41.04 | 78.26 | 71.67 | 74.52 |  |  |  |  |  |  |  |
| TRINITY_DN3464_c0_g1 | 1.67 | 2.47 | 2.34 | 4.71 | 3.8 | 4.71 |  |  |  |  |  |  |  |
| TRINITY_DN4429_c0_g2 | 11.39 | 10.98 | 11.32 | 84.34 | 83.09 | 73.4 |  |  |  |  |  |  |  |
| TRINITY_DN7988_c0_g1 | 5.69 | 6.75 | 5.2 | 22.8 | 19.23 | 19.35 |  |  |  |  |  |  |  |
| TRINITY_DN7521_c0_g1 | 7.78 | 8.84 | 8.5 | 24.79 | 20.85 | 19.82 |  |  |  |  |  |  |  |
| TRINITY_DN7740_c0_g1 | 7.33 | 8.05 | 7.86 | 1.12 | 1.16 | 1.9 |  |  |  |  |  |  |  |
| TRINITY_DN1583_c0_g2 | 103.88 | 103.01 | 99.54 | 24.36 | 24.74 | 24.13 |  |  |  |  |  |  |  |
| TRINITY_DN45204_c0_g1 | 32.4 | 30.04 | 17.76 | 7.12 | 9.53 | 4.57 |  |  |  |  |  |  |  |
| TRINITY_DN724_c0_g1 | 54.82 | 50.6 | 51.3 | 13.44 | 15.14 | 12.01 |  |  |  |  |  |  |  |
| TRINITY_DN20336_c1_g1 | 8.19 | 8.29 | 8.38 | 2.49 | 1.54 | 0.95 |  |  |  |  |  |  |  |
| TRINITY_DN42900_c0_g2 | 22.63 | 28.08 | 25.13 | 5.19 | 4.28 | 7.4 |  |  |  |  |  |  |  |
| TRINITY_DN4538_c0_g1 | 441.24 | 452.88 | 450.4 | 146.26 | 134.2 | 122.78 |  |  |  |  |  |  |  |
| TRINITY_DN2664_c1_g1 | 9.38 | 8.33 | 8.06 | 3.13 | 3.81 | 1.53 |  |  |  |  |  |  |  |
| TRINITY_DN10658_c0_g1 | 2.56 | 3.33 | 2.43 | 0.22 | 0.54 | 1.37 |  |  |  |  |  |  |  |
| TRINITY_DN567_c0_g1 | 12.49 | 10.83 | 12.12 | 43.94 | 43.76 | 38.63 |  |  |  |  |  |  |  |
| TRINITY_DN9156_c0_g1 | 22.44 | 21.31 | 21.2 | 42.45 | 39.59 | 36.62 |  |  |  |  |  |  |  |
| TRINITY_DN6379_c0_g1 | 45.86 | 48.78 | 45.67 | 190.34 | 187.92 | 164.98 |  |  |  |  |  |  |  |
| TRINITY_DN4814_c0_g3 | 0.92 | 0.86 | 0.9 | 0.13 | 0.06 | 0.35 |  |  |  |  |  |  |  |
| TRINITY_DN3234_c0_g1 | 45.66 | 45.36 | 46.09 | 127.69 | 125.07 | 116.74 |  |  |  |  |  |  |  |
| TRINITY_DN35429_c0_g2 | 46.44 | 45.43 | 44.74 | 245.88 | 223.34 | 204.27 |  |  |  |  |  |  |  |
| TRINITY_DN3918_c1_g1 | 1.1 | 1.26 | 1.73 | 2.01 | 2.95 | 2.81 |  |  |  |  |  |  |  |
| TRINITY_DN41482_c0_g2 | 3.02 | 1.14 | 2.45 | 0.52 | 0.14 | 1.63 |  |  |  |  |  |  |  |
| TRINITY_DN1739_c0_g3 | 68.9 | 70.25 | 74.13 | 283.5 | 280.4 | 250.56 |  |  |  |  |  |  |  |
| TRINITY_DN3785_c0_g2 | 8.62 | 8.24 | 7.74 | 60.06 | 56.97 | 50.58 |  |  |  |  |  |  |  |
| TRINITY_DN7019_c0_g1 | 9.54 | 9.1 | 6.57 | 0 | 0.62 | 0.08 |  |  |  |  |  |  |  |
| TRINITY_DN466_c0_g1 | 75.04 | 73.02 | 69.38 | 114.88 | 114.09 | 93.48 |  |  |  |  |  |  |  |
| TRINITY_DN34105_c0_g1 | 2.52 | 3.42 | 3.6 | 2.96 | 0.9 | 0.37 |  |  |  |  |  |  |  |
| TRINITY_DN3990_c0_g1 | 4.37 | 3.65 | 3.8 | 0.12 | 0.17 | 0.04 |  |  |  |  |  |  |  |
| TRINITY_DN33556_c0_g1 | 5.72 | 6.11 | 6.19 | 1.93 | 1.83 | 1.76 |  |  |  |  |  |  |  |
| TRINITY_DN448_c0_g3 | 9.04 | 8.27 | 8.19 | 0.31 | 1.63 | 0.78 |  |  |  |  |  |  |  |
| **Sulfur metabolism (ko00920)** | | | | | | | | | | | | | |
| **Genes** | | | | | | | **Metabolites** | | | | | | |
| TRINITY_DN1797_c5_g1 | 18.55 | 18.31 | 20.95 | 77.11 | 85.89 | 75.02 | Sulfate | 10795479.21 | 12746641.38 | 14484581.39 | 4514957.628 | 4119906.737 | 6282653.882 |
| TRINITY_DN5127_c0_g1 | 3.47 | 4.38 | 3.3 | 0.24 | 1.12 | 0.26 | Taurine | 37228.93216 | 40581.20539 | 41262.35931 | 153350.7118 | 175048.7594 | 148147.8078 |
| TRINITY_DN3099_c0_g2 | 111.74 | 106.51 | 107.25 | 252.17 | 236.63 | 218.23 | L-Serine | 1654326.186 | 1506170.737 | 1482295.541 | 2812208.811 | 2657323.226 | 2703792.312 |
| TRINITY_DN8111_c0_g1 | 12.73 | 14.97 | 12.84 | 4.15 | 4.75 | 4.41 | Succinic Acid | 5715097.091 | 5813509.878 | 6068665.156 | 8391498.124 | 8735064.16 | 8545233.673 |
| TRINITY_DN2310_c0_g3 | 2.25 | 1.89 | 2.28 | 0.03 | 0 | 0 | L-Homoserine | 1902787.489 | 1694921.465 | 1872179.17 | 3161073.96 | 2862602.175 | 2931724.281 |
| TRINITY_DN6933_c0_g1 | 9.82 | 13.46 | 8.17 | 39.41 | 38.37 | 30.74 |  |  |  |  |  |  |  |
| TRINITY_DN4369_c0_g1 | 11.09 | 11.92 | 11.63 | 128.82 | 127.49 | 107.22 |  |  |  |  |  |  |  |
| TRINITY_DN15123_c0_g1 | 15.95 | 16.08 | 15.89 | 1.13 | 1.54 | 1.02 |  |  |  |  |  |  |  |
| TRINITY_DN53_c3_g1 | 156.96 | 141.59 | 147.93 | 6.28 | 10.01 | 6.8 |  |  |  |  |  |  |  |
| TRINITY_DN1772_c0_g1 | 2.38 | 2.88 | 2.12 | 12.65 | 12.61 | 11.88 |  |  |  |  |  |  |  |
| TRINITY_DN7227_c0_g1 | 21.98 | 23.02 | 22.16 | 34.49 | 35.18 | 30.72 |  |  |  |  |  |  |  |
| TRINITY_DN3470_c0_g1 | 93.48 | 94.11 | 87.97 | 2.15 | 4.12 | 2.56 |  |  |  |  |  |  |  |
| TRINITY_DN26168_c0_g2 | 30.61 | 35.12 | 34.27 | 7.14 | 10.12 | 8.27 |  |  |  |  |  |  |  |
| TRINITY_DN1263_c2_g1 | 1280.17 | 1185.15 | 1214.14 | 56.27 | 50.77 | 49.22 |  |  |  |  |  |  |  |
| TRINITY_DN8752_c0_g1 | 17.04 | 16.89 | 15.5 | 59.81 | 52.13 | 60.9 |  |  |  |  |  |  |  |
| TRINITY_DN7771_c0_g1 | 1.83 | 1.46 | 1.57 | 4.32 | 2.78 | 2.35 |  |  |  |  |  |  |  |
| TRINITY_DN19453_c0_g1 | 19.91 | 21.37 | 24.35 | 0 | 0 | 0 |  |  |  |  |  |  |  |
| TRINITY_DN926_c1_g1 | 22.18 | 23.12 | 20.65 | 224.37 | 295.28 | 232.94 |  |  |  |  |  |  |  |
|  |  |  |  |  |  |  |  |  |  |  |  |  |  |
| **HQI/HQO group** |  |  |  |  |  |  |  |  |  |  |  |  |  |
| **ID-Gene** | **HQI01** | **HQI02** | **HQI03** | **HQO01** | **HQO02** | **HQO03** | **ID-Meta** | **HQI01** | **HQI02** | **HQI03** | **HQO01** | **HQO02** | **HQO03** |
| **Flavonoid biosynthesis (ko00941)** | | | | | | | | | | | | | |
| **Genes** | | | | | | | **Metabolites** | | | | | | |
| TRINITY_DN8213_c1_g2 | 4.29 | 5.43 | 3.34 | 182.64 | 188.22 | 172.01 | Taxifolin | 1257359.434 | 1356939.673 | 1351384.148 | 10.00190286 | 10.00190286 | 10.00190286 |
| TRINITY_DN37328_c0_g1 | 83.8 | 93.19 | 89.49 | 5.12 | 5.42 | 4.09 | Kaempferol | 2028583.336 | 2244179.48 | 2357482.414 | 770619.1331 | 727581.5076 | 774004.9617 |
| TRINITY_DN8789_c0_g1 | 12.6 | 13.49 | 12.86 | 521.77 | 509.42 | 545.58 | Dihydrokaempferol | 29031461.75 | 33737748.83 | 34511793.13 | 4963470.048 | 4800563.936 | 5074278.548 |
| TRINITY_DN4267_c0_g1 | 7.32 | 8.01 | 6.87 | 57.09 | 59.35 | 54.24 | Dihydromyricetin | 1551894.935 | 1607315.008 | 1510078.495 | 18732.22512 | 20323.16157 | 31833.81516 |
| TRINITY_DN9_c2_g1 | 25.27 | 28.55 | 29.75 | 165.93 | 175.4 | 161.18 | Naringenin Chalcone | 3950301.484 | 4092691.436 | 3956424.893 | 1969612.76 | 1611534.213 | 1992205.082 |
| TRINITY_DN721_c0_g1 | 12.64 | 12.84 | 12.42 | 5455.89 | 5129.94 | 5086.66 | Luteolin | 3023395.453 | 3986480.149 | 3615289.838 | 1763959.786 | 2025763.481 | 2158368.824 |
| TRINITY_DN1724_c0_g1 | 1914.81 | 2002.74 | 2049.06 | 132.19 | 129.58 | 124.97 | Myricetin | 17867163.89 | 23439420.62 | 23720942.18 | 115847.1804 | 93849.70447 | 96856.34268 |
| TRINITY_DN6410_c0_g1 | 7.02 | 7.49 | 7.62 | 772.98 | 738.19 | 772.24 | Tricetin | 1727104.7 | 2201213.326 | 2230451.141 | 10.00190286 | 10.00190286 | 10.00190286 |
| TRINITY_DN18925_c1_g1 | 1.9 | 1.24 | 2.16 | 0.16 | 0.32 | 0.59 | Phloretin | 5534929.609 | 6451277.286 | 6110385.886 | 587426.9751 | 598925.9394 | 628245.0376 |
| TRINITY_DN8789_c0_g2 | 0.08 | 0 | 0.08 | 1.9 | 1.13 | 1.92 | (+)-Gallocatechin | 280024.5614 | 263730.3349 | 230520.6091 | 10.00190286 | 10.00190286 | 10.00190286 |
| TRINITY_DN2275_c0_g2 | 30.44 | 25.61 | 34.66 | 303.81 | 285.74 | 303.93 | Apigenin | 540474.7751 | 588192.2335 | 579170.4348 | 273099.1108 | 160466.0224 | 125807.9615 |
| TRINITY_DN1028_c0_g1 | 14.73 | 17.8 | 17.07 | 3.21 | 1.25 | 7.68 | Pelargonidin | 931459.1264 | 702655.6146 | 806207.315 | 517327.9189 | 505991.4152 | 453510.7209 |
| TRINITY_DN702_c0_g1 | 4.65 | 5.14 | 4.73 | 0.14 | 0.22 | 0.17 | Phlorizin | 3018665.574 | 3618462.165 | 3042051.85 | 130512.5598 | 112001.3712 | 169549.6097 |
| TRINITY_DN11391_c0_g1 | 1.75 | 2.11 | 2.18 | 187.78 | 162.73 | 174.67 | (+)-Taxifolin | 1481174.345 | 1545406.093 | 1481386.92 | 10.00190286 | 10.00190286 | 10.00190286 |
| TRINITY_DN23579_c0_g1 | 5.69 | 5.94 | 4.96 | 0.38 | 1.42 | 0.29 |  |  |  |  |  |  |  |
| TRINITY_DN12660_c0_g1 | 0.58 | 0.9 | 1.16 | 4.72 | 2.95 | 5 |  |  |  |  |  |  |  |
| TRINITY_DN6367_c0_g2 | 20.93 | 21.84 | 22.38 | 109.51 | 104.47 | 99.94 |  |  |  |  |  |  |  |
| TRINITY_DN48713_c0_g1 | 0.26 | 0.22 | 0.26 | 11.96 | 10.97 | 12.14 |  |  |  |  |  |  |  |
| TRINITY_DN756_c0_g1 | 1.37 | 1.49 | 2.21 | 0.97 | 0.99 | 0.6 |  |  |  |  |  |  |  |
| TRINITY_DN5866_c0_g1 | 1.33 | 1 | 1.32 | 28.42 | 28.53 | 24.72 |  |  |  |  |  |  |  |
| TRINITY_DN7608_c0_g1 | 50.49 | 56.79 | 62.51 | 5.13 | 6.73 | 3.56 |  |  |  |  |  |  |  |
| TRINITY_DN36773_c0_g1 | 0.95 | 3.7 | 2.7 | 0.34 | 1.2 | 1.98 |  |  |  |  |  |  |  |
| TRINITY_DN11393_c0_g1 | 24.72 | 21.85 | 25.01 | 7.11 | 8.98 | 8.81 |  |  |  |  |  |  |  |
| TRINITY_DN9802_c0_g2 | 6.61 | 11.11 | 14.52 | 0.58 | 1.21 | 0.51 |  |  |  |  |  |  |  |
| TRINITY_DN4447_c1_g2 | 4.03 | 4.47 | 3.38 | 19.69 | 16.79 | 18.12 |  |  |  |  |  |  |  |
| TRINITY_DN73_c0_g2 | 2.28 | 2.29 | 2.66 | 93.7 | 80.16 | 83.71 |  |  |  |  |  |  |  |
| TRINITY_DN3048_c1_g1 | 9.24 | 10.58 | 9.72 | 1 | 0.97 | 1.03 |  |  |  |  |  |  |  |
| TRINITY_DN2275_c0_g1 | 7.18 | 8.27 | 6.66 | 2.61 | 3.29 | 4.01 |  |  |  |  |  |  |  |
| TRINITY_DN536_c0_g1 | 14.63 | 17.02 | 17.21 | 375.42 | 375.44 | 351.79 |  |  |  |  |  |  |  |
| TRINITY_DN38245_c0_g1 | 6.74 | 8.6 | 6.49 | 1029.9 | 969.59 | 1002.18 |  |  |  |  |  |  |  |
| TRINITY_DN9130_c1_g1 | 2.81 | 2.86 | 2.35 | 15.95 | 16.72 | 18.34 |  |  |  |  |  |  |  |
| TRINITY_DN1496_c0_g1 | 3.02 | 3.34 | 4.12 | 16.09 | 17.68 | 15.23 |  |  |  |  |  |  |  |
| TRINITY_DN479_c0_g1 | 5.68 | 7.65 | 4.63 | 41.84 | 39.66 | 41.48 |  |  |  |  |  |  |  |
| TRINITY_DN2165_c0_g2 | 5.62 | 6.1 | 6.84 | 42.59 | 41.47 | 39.32 |  |  |  |  |  |  |  |
| TRINITY_DN2165_c0_g3 | 40.97 | 43.05 | 43.08 | 14 | 15.43 | 13.93 |  |  |  |  |  |  |  |
| TRINITY_DN5586_c1_g1 | 138.67 | 148.46 | 144.72 | 974.82 | 922.13 | 932.66 |  |  |  |  |  |  |  |
| TRINITY_DN8313_c0_g1 | 3.59 | 3.46 | 3.33 | 27.26 | 26.75 | 27.53 |  |  |  |  |  |  |  |
| TRINITY_DN20773_c0_g2 | 52.23 | 56.76 | 64.61 | 18.8 | 16.1 | 18.97 |  |  |  |  |  |  |  |
| TRINITY_DN4805_c0_g1 | 8.29 | 10.36 | 7.5 | 5.5 | 4.01 | 5.92 |  |  |  |  |  |  |  |
| TRINITY_DN9466_c3_g1 | 1.07 | 1.59 | 1.5 | 0.19 | 0.25 | 0 |  |  |  |  |  |  |  |
| TRINITY_DN1327_c0_g1 | 2.75 | 3.21 | 2.37 | 29.34 | 37.58 | 28.2 |  |  |  |  |  |  |  |
| TRINITY_DN73_c0_g1 | 10.53 | 13.49 | 10.78 | 0.06 | 0.15 | 0.06 |  |  |  |  |  |  |  |
| TRINITY_DN2424_c1_g1 | 0.9 | 1.73 | 1.42 | 0 | 0 | 0 |  |  |  |  |  |  |  |
| TRINITY_DN11722_c0_g1 | 24.41 | 26.84 | 37.6 | 11.5 | 12.33 | 10.1 |  |  |  |  |  |  |  |
| TRINITY_DN9306_c0_g1 | 20.58 | 19.72 | 21.4 | 90.17 | 83.92 | 89.6 |  |  |  |  |  |  |  |
| TRINITY_DN12060_c0_g3 | 12.61 | 13.15 | 12.61 | 11.16 | 9.83 | 8.87 |  |  |  |  |  |  |  |
| **Purine metabolism (ko00230)** | | | | | | | | | | | | | |
| **Genes** | | | | | | | **Metabolites** | | | | | | |
| TRINITY_DN2949_c0_g1 | 7.78 | 8.76 | 6.59 | 2.28 | 1.93 | 1.08 | Oxalic Acid | 246122.5681 | 226634.576 | 278177.4921 | 75625.7805 | 91614.44148 | 75804 |
| TRINITY_DN1994_c0_g1 | 17.64 | 19.86 | 17.87 | 12.17 | 14.32 | 12.8 | Guanosine | 2369952.88 | 2459920.895 | 2373839.221 | 926885.5372 | 780037.5235 | 917759.4268 |
| TRINITY_DN5645_c0_g1 | 5.05 | 4.52 | 5.29 | 29.42 | 34.13 | 27.55 | Sulfate | 14827937.26 | 16956793.3 | 16806710.96 | 2186411.888 | 2187851.253 | 2297305.096 |
| TRINITY_DN5640_c0_g1 | 1.78 | 2.28 | 1.97 | 6.38 | 7.07 | 6.11 | L-Glutamine | 24695885.14 | 25621655.03 | 24625591.75 | 4197288.911 | 3920147.104 | 3644458.786 |
| TRINITY_DN11731_c0_g1 | 28.26 | 27.7 | 29.98 | 9.07 | 11.09 | 9.87 | 5-Hydroxyisourate | 374.7693557 | 513.1119769 | 760.3976651 | 10.00190286 | 10.00190286 | 10.00190286 |
| TRINITY_DN2085_c1_g2 | 10.54 | 8.38 | 10.06 | 36.58 | 41.88 | 40.83 | Inosine | 79172.71185 | 86241.70661 | 77085.83277 | 170857.2788 | 180341.8716 | 210585.5536 |
| TRINITY_DN772_c1_g2 | 1.83 | 1.76 | 2.04 | 47.88 | 47.69 | 47.79 | Adenosine | 59500434.76 | 59226547.72 | 60280925.45 | 33997726.11 | 33083398.89 | 32318916.66 |
| TRINITY_DN437_c0_g2 | 9.21 | 9.56 | 10.43 | 6.07 | 7.11 | 6.57 |  |  |  |  |  |  |  |
| TRINITY_DN8624_c0_g1 | 12.37 | 14.21 | 13.38 | 9.89 | 7.79 | 11.22 |  |  |  |  |  |  |  |
| TRINITY_DN8358_c0_g1 | 0.55 | 0.33 | 0.51 | 17.49 | 16.81 | 12.42 |  |  |  |  |  |  |  |
| TRINITY_DN10206_c0_g1 | 44.51 | 44.23 | 48.98 | 7.84 | 10.93 | 6.53 |  |  |  |  |  |  |  |
| TRINITY_DN871_c0_g1 | 39.99 | 44.81 | 45.13 | 13.39 | 12.56 | 11.3 |  |  |  |  |  |  |  |
| TRINITY_DN20677_c0_g3 | 6.66 | 6.57 | 6.71 | 44.02 | 42.42 | 44.96 |  |  |  |  |  |  |  |
| TRINITY_DN15198_c0_g1 | 2.84 | 2.9 | 3.12 | 12.52 | 12.4 | 12.3 |  |  |  |  |  |  |  |
| TRINITY_DN1929_c0_g1 | 7.68 | 9.82 | 8.24 | 32.62 | 31.85 | 32.87 |  |  |  |  |  |  |  |
| TRINITY_DN470_c0_g2 | 41.49 | 38.72 | 41.78 | 15.26 | 16.03 | 18.25 |  |  |  |  |  |  |  |
| TRINITY_DN576_c1_g2 | 4.96 | 4.83 | 4.91 | 1.24 | 1.71 | 1.86 |  |  |  |  |  |  |  |
| TRINITY_DN6215_c0_g2 | 11.21 | 10.89 | 10.33 | 8.55 | 6.95 | 7.46 |  |  |  |  |  |  |  |
| TRINITY_DN12779_c0_g1 | 25.46 | 21.84 | 22.91 | 14.48 | 13.21 | 12.09 |  |  |  |  |  |  |  |
| TRINITY_DN3012_c1_g1 | 73.99 | 78.3 | 74.8 | 9.94 | 10.86 | 10.29 |  |  |  |  |  |  |  |
| TRINITY_DN43542_c0_g1 | 0.51 | 0.13 | 0.03 | 1.79 | 2.19 | 2.32 |  |  |  |  |  |  |  |
| TRINITY_DN11981_c0_g1 | 18.64 | 19.19 | 18.16 | 0.33 | 2.34 | 1.27 |  |  |  |  |  |  |  |
| TRINITY_DN5042_c0_g1 | 4.62 | 3.84 | 4.06 | 24.84 | 32.14 | 28.67 |  |  |  |  |  |  |  |
| TRINITY_DN4664_c0_g1 | 2.12 | 1.08 | 1.18 | 36.02 | 32.95 | 34.02 |  |  |  |  |  |  |  |
| TRINITY_DN7074_c0_g1 | 7.56 | 8.63 | 7.85 | 45.53 | 48.45 | 46.53 |  |  |  |  |  |  |  |
| TRINITY_DN5589_c0_g1 | 4.71 | 5.15 | 5.08 | 4.28 | 3.32 | 4.2 |  |  |  |  |  |  |  |
| TRINITY_DN181_c0_g1 | 5.56 | 4.81 | 4.57 | 99.66 | 112.39 | 86.41 |  |  |  |  |  |  |  |
| TRINITY_DN1299_c0_g1 | 52.28 | 61.65 | 56.7 | 14.49 | 16.31 | 12.09 |  |  |  |  |  |  |  |
| TRINITY_DN8755_c0_g1 | 50.17 | 51.87 | 54.87 | 27.32 | 25.8 | 28.49 |  |  |  |  |  |  |  |
| TRINITY_DN17264_c0_g1 | 9.42 | 10.23 | 9.23 | 3.32 | 4.88 | 3.85 |  |  |  |  |  |  |  |
| TRINITY_DN7626_c0_g1 | 1.77 | 1.79 | 2.27 | 13.1 | 12.5 | 13.47 |  |  |  |  |  |  |  |
| TRINITY_DN6107_c0_g1 | 0.41 | 0.49 | 2.16 | 5.44 | 6.13 | 5.6 |  |  |  |  |  |  |  |
| TRINITY_DN1202_c0_g1 | 9.24 | 11.66 | 12.62 | 55.34 | 56.74 | 47.69 |  |  |  |  |  |  |  |
| TRINITY_DN5563_c0_g2 | 1.25 | 2.46 | 1.75 | 8.06 | 8.8 | 7.71 |  |  |  |  |  |  |  |
| TRINITY_DN5913_c0_g1 | 0.64 | 0.61 | 0.73 | 3.1 | 3.3 | 3.26 |  |  |  |  |  |  |  |
| TRINITY_DN3578_c0_g1 | 1.64 | 1.7 | 1.77 | 1.61 | 1.52 | 1.32 |  |  |  |  |  |  |  |
| TRINITY_DN14976_c0_g1 | 32.26 | 36.58 | 38.65 | 4.69 | 5.53 | 5.56 |  |  |  |  |  |  |  |
| TRINITY_DN1641_c0_g1 | 6.91 | 7.77 | 10.24 | 45.69 | 51.11 | 50.42 |  |  |  |  |  |  |  |
| TRINITY_DN7367_c1_g1 | 4.88 | 5.62 | 6.99 | 4.06 | 4.23 | 5.16 |  |  |  |  |  |  |  |
| TRINITY_DN1543_c1_g1 | 12.42 | 13.85 | 12.15 | 61.06 | 56.13 | 51.12 |  |  |  |  |  |  |  |
| TRINITY_DN4708_c0_g1 | 23.78 | 28.34 | 26.41 | 116.22 | 111.87 | 119.75 |  |  |  |  |  |  |  |
| TRINITY_DN2669_c1_g1 | 41.72 | 48.92 | 42.05 | 33.51 | 38.2 | 37.97 |  |  |  |  |  |  |  |
| TRINITY_DN43542_c0_g2 | 2.36 | 2.54 | 1.81 | 9.92 | 7.99 | 10.23 |  |  |  |  |  |  |  |
| TRINITY_DN2453_c0_g1 | 11.52 | 19.79 | 16.77 | 7.04 | 4.59 | 5.76 |  |  |  |  |  |  |  |
| TRINITY_DN8554_c0_g1 | 3.18 | 2.08 | 2.41 | 0.5 | 0.08 | 0.97 |  |  |  |  |  |  |  |
| TRINITY_DN5155_c0_g1 | 1.22 | 1.38 | 1.52 | 0.52 | 0.56 | 0.49 |  |  |  |  |  |  |  |
| TRINITY_DN190_c0_g1 | 48.12 | 54.64 | 52.21 | 218.03 | 226.36 | 232.06 |  |  |  |  |  |  |  |
| **Phenylpropanoid biosynthesis (ko00940)** | | | | | | | | | | | | | |
| **Genes** | | | | | | | **Metabolites** | | | | | | |
| TRINITY_DN12429_c0_g1 | 2939.82 | 3099.96 | 3067.73 | 151.3 | 155.55 | 145.55 | Ferulic Acid | 9344288.683 | 10182382.43 | 10499521.04 | 2405865.738 | 2159336.079 | 2151855.001 |
| TRINITY_DN4703_c0_g1 | 7.71 | 9.92 | 9.08 | 238.3 | 241.68 | 233.76 | Coniferaldehyde | 8108694.525 | 6988721.296 | 7578619.168 | 2009092.068 | 2033539.568 | 1903846.818 |
| TRINITY_DN28868_c0_g1 | 12.06 | 13.28 | 10.91 | 8.04 | 7.67 | 5.59 | P-Coumaryl Alcohol | 10.00190286 | 10.00190286 | 10.00190286 | 31267.00948 | 28755.60295 | 19171.90942 |
| TRINITY_DN18055_c1_g1 | 5.87 | 4.23 | 5.78 | 1.4 | 1.55 | 1.49 | Eugenol | 221923.5641 | 218826.8273 | 216516.1202 | 248674.8211 | 239909.0363 | 253147.8407 |
| TRINITY_DN3886_c1_g1 | 2.11 | 4.03 | 2 | 190.16 | 201.18 | 203.02 | L-Phenylalanine | 27773272.9 | 27507221.99 | 27882291.3 | 52956442.52 | 51192655.74 | 49854807.61 |
| TRINITY_DN17373_c0_g1 | 239.05 | 264.84 | 270.21 | 201.15 | 176.55 | 209.04 | L-Tyrosine | 1065119.507 | 1142581.713 | 1060558.464 | 456083.8822 | 462964.7977 | 484870.2472 |
| TRINITY_DN9306_c0_g1 | 20.58 | 19.72 | 21.4 | 90.17 | 83.92 | 89.6 | Isochavicol | 1550.259608 | 1745.00963 | 1739.046024 | 1980.044474 | 2201.253322 | 2174.445871 |
| TRINITY_DN20611_c0_g2 | 11.54 | 12.76 | 13.61 | 79.78 | 81.39 | 80.1 | Caffeic Acid | 4815565.91 | 4925400.093 | 5076591.025 | 1460111.517 | 1602651.253 | 1445259.725 |
| TRINITY_DN23855_c0_g2 | 3.93 | 5.11 | 4.55 | 18.36 | 24.62 | 20.23 |  |  |  |  |  |  |  |
| TRINITY_DN4519_c0_g1 | 0.08 | 0 | 0 | 3.32 | 3.07 | 1.18 |  |  |  |  |  |  |  |
| TRINITY_DN7042_c0_g1 | 29.09 | 21.46 | 21.39 | 4220.53 | 4114.51 | 4160.37 |  |  |  |  |  |  |  |
| TRINITY_DN6781_c0_g1 | 2.23 | 2.33 | 2.32 | 9.9 | 10.01 | 7.76 |  |  |  |  |  |  |  |
| TRINITY_DN32563_c0_g3 | 0.96 | 0.71 | 0.64 | 28.61 | 30.73 | 33.02 |  |  |  |  |  |  |  |
| TRINITY_DN2606_c0_g1 | 1.91 | 1.73 | 2.1 | 21.27 | 24.78 | 18.5 |  |  |  |  |  |  |  |
| TRINITY_DN8644_c0_g1 | 3.92 | 2.52 | 2.75 | 0.13 | 0.06 | 0.11 |  |  |  |  |  |  |  |
| TRINITY_DN15104_c0_g3 | 6.83 | 7.48 | 8.33 | 4.98 | 4.41 | 4.26 |  |  |  |  |  |  |  |
| TRINITY_DN29594_c0_g3 | 7.5 | 6.34 | 7.45 | 0.18 | 0.35 | 0.43 |  |  |  |  |  |  |  |
| TRINITY_DN6185_c2_g1 | 1.49 | 1.22 | 1.48 | 15.75 | 15.77 | 13.98 |  |  |  |  |  |  |  |
| TRINITY_DN10581_c0_g4 | 5.13 | 5.45 | 5.08 | 29.21 | 32.97 | 29.29 |  |  |  |  |  |  |  |
| TRINITY_DN3597_c1_g1 | 203.83 | 169.26 | 169.32 | 103.05 | 88.98 | 100.63 |  |  |  |  |  |  |  |
| TRINITY_DN4805_c0_g1 | 8.29 | 10.36 | 7.5 | 5.5 | 4.01 | 5.92 |  |  |  |  |  |  |  |
| TRINITY_DN21588_c0_g2 | 8.27 | 8.57 | 9 | 3.2 | 3.81 | 4.12 |  |  |  |  |  |  |  |
| TRINITY_DN11404_c0_g1 | 115.05 | 78.37 | 75.68 | 16.85 | 26.27 | 18.8 |  |  |  |  |  |  |  |
| TRINITY_DN9332_c0_g1 | 1.98 | 2.91 | 3.14 | 306.98 | 321.4 | 325.24 |  |  |  |  |  |  |  |
| TRINITY_DN5200_c0_g1 | 1064.98 | 1212.77 | 1223.41 | 922.37 | 928.86 | 911.99 |  |  |  |  |  |  |  |
| TRINITY_DN24941_c0_g2 | 1.2 | 0.89 | 1.95 | 6.93 | 7.65 | 5.59 |  |  |  |  |  |  |  |
| TRINITY_DN2085_c3_g2 | 28.77 | 27.85 | 44.28 | 5.11 | 3.64 | 4.57 |  |  |  |  |  |  |  |
| TRINITY_DN22183_c0_g1 | 5.9 | 6.2 | 0.87 | 218.36 | 183.66 | 156.38 |  |  |  |  |  |  |  |
| TRINITY_DN8313_c0_g1 | 3.59 | 3.46 | 3.33 | 27.26 | 26.75 | 27.53 |  |  |  |  |  |  |  |
| TRINITY_DN2853_c0_g1 | 1.26 | 1.16 | 2.11 | 10.04 | 9.96 | 10.1 |  |  |  |  |  |  |  |
| TRINITY_DN2739_c2_g1 | 0.86 | 1.1 | 1.29 | 16.19 | 17.25 | 15.49 |  |  |  |  |  |  |  |
| TRINITY_DN22_c0_g3 | 4.09 | 3.05 | 2.89 | 73.28 | 69.89 | 81.34 |  |  |  |  |  |  |  |
| TRINITY_DN4079_c0_g1 | 215.29 | 226.86 | 237.66 | 7058.66 | 6638.38 | 6295.28 |  |  |  |  |  |  |  |
| TRINITY_DN22183_c0_g2 | 0 | 0 | 0 | 4.05 | 3.62 | 4.19 |  |  |  |  |  |  |  |
| TRINITY_DN47149_c0_g1 | 0.18 | 0.28 | 0.3 | 3.97 | 5.16 | 3.46 |  |  |  |  |  |  |  |
| TRINITY_DN1515_c0_g2 | 1.95 | 1.91 | 3.92 | 0.42 | 0.46 | 0.68 |  |  |  |  |  |  |  |
| TRINITY_DN1923_c0_g2 | 0.15 | 0 | 0.16 | 13.19 | 16.95 | 13.03 |  |  |  |  |  |  |  |
| TRINITY_DN4061_c0_g1 | 1.2 | 1.83 | 1 | 24.3 | 23 | 24.81 |  |  |  |  |  |  |  |
| TRINITY_DN2377_c4_g2 | 1.63 | 2.13 | 2.77 | 9.32 | 9.93 | 10.41 |  |  |  |  |  |  |  |
| TRINITY_DN479_c0_g1 | 5.68 | 7.65 | 4.63 | 41.84 | 39.66 | 41.48 |  |  |  |  |  |  |  |
| TRINITY_DN5169_c0_g1 | 1.56 | 2.92 | 2.39 | 16.37 | 13.16 | 10.36 |  |  |  |  |  |  |  |
| TRINITY_DN20068_c0_g3 | 9.8 | 7.52 | 8.19 | 0.16 | 0.16 | 0.59 |  |  |  |  |  |  |  |
| TRINITY_DN20068_c0_g4 | 2.02 | 1.61 | 3.15 | 0.06 | 0.12 | 0 |  |  |  |  |  |  |  |
| TRINITY_DN44436_c0_g1 | 0.15 | 0.99 | 0.05 | 6.95 | 6 | 7.64 |  |  |  |  |  |  |  |
| TRINITY_DN12306_c0_g1 | 12.13 | 15.84 | 14.1 | 668.99 | 671.31 | 638.8 |  |  |  |  |  |  |  |
| TRINITY_DN2945_c0_g1 | 3.95 | 2.48 | 4.37 | 0.98 | 0.49 | 0.05 |  |  |  |  |  |  |  |
| TRINITY_DN8852_c0_g1 | 8.11 | 7.35 | 8.5 | 1048.12 | 1055.95 | 1006.85 |  |  |  |  |  |  |  |
| TRINITY_DN368_c2_g1 | 11.72 | 10.98 | 13.27 | 1.25 | 0.71 | 0.47 |  |  |  |  |  |  |  |
| TRINITY_DN5200_c0_g2 | 96.96 | 107.08 | 105.48 | 4.53 | 5.96 | 3.16 |  |  |  |  |  |  |  |
| TRINITY_DN2275_c0_g1 | 7.18 | 8.27 | 6.66 | 2.61 | 3.29 | 4.01 |  |  |  |  |  |  |  |
| TRINITY_DN7387_c0_g1 | 3.8 | 3.45 | 3.68 | 72.43 | 59.78 | 61.61 |  |  |  |  |  |  |  |
| TRINITY_DN7476_c0_g1 | 9.44 | 10.4 | 10.92 | 0.29 | 0.79 | 1.25 |  |  |  |  |  |  |  |
| TRINITY_DN505_c0_g2 | 6.92 | 7.75 | 6.78 | 30.83 | 33.08 | 26.84 |  |  |  |  |  |  |  |
| TRINITY_DN215_c3_g2 | 0.74 | 0.56 | 1.8 | 20.27 | 20.54 | 23.68 |  |  |  |  |  |  |  |
| TRINITY_DN536_c0_g1 | 14.63 | 17.02 | 17.21 | 375.42 | 375.44 | 351.79 |  |  |  |  |  |  |  |
| TRINITY_DN1717_c0_g3 | 24.74 | 24.95 | 22.12 | 289.69 | 278.75 | 297.47 |  |  |  |  |  |  |  |
| TRINITY_DN8399_c0_g1 | 31.45 | 35.96 | 35.07 | 213.16 | 200.87 | 226.15 |  |  |  |  |  |  |  |
| TRINITY_DN2738_c0_g3 | 0.22 | 0 | 0.07 | 11.36 | 14.24 | 11.43 |  |  |  |  |  |  |  |
| TRINITY_DN417_c0_g2 | 11.19 | 12.51 | 10.95 | 96.68 | 98.47 | 90.18 |  |  |  |  |  |  |  |
| TRINITY_DN8100_c0_g1 | 2.01 | 3.33 | 3.07 | 11.42 | 13.48 | 14.19 |  |  |  |  |  |  |  |
| TRINITY_DN8153_c1_g1 | 7.6 | 9.21 | 8.31 | 0.86 | 0.89 | 0.73 |  |  |  |  |  |  |  |
| TRINITY_DN7083_c0_g1 | 1.89 | 1.6 | 1.62 | 0.81 | 1.41 | 1.24 |  |  |  |  |  |  |  |
| TRINITY_DN10515_c0_g2 | 11.36 | 12.36 | 12.16 | 86.2 | 83.61 | 77.91 |  |  |  |  |  |  |  |
| TRINITY_DN22183_c0_g3 | 2.42 | 0 | 1.63 | 94.43 | 126.82 | 81.81 |  |  |  |  |  |  |  |
| TRINITY_DN32563_c0_g2 | 3.41 | 2.96 | 2.95 | 1.47 | 1.34 | 1.98 |  |  |  |  |  |  |  |
| TRINITY_DN25062_c0_g1 | 2.02 | 3.23 | 1.59 | 0.12 | 0.36 | 0 |  |  |  |  |  |  |  |
| TRINITY_DN40451_c0_g1 | 70.27 | 75.7 | 74.18 | 4.01 | 4.53 | 4.27 |  |  |  |  |  |  |  |
| TRINITY_DN21_c0_g2 | 2.82 | 2.33 | 2.07 | 13.58 | 12.53 | 16.55 |  |  |  |  |  |  |  |
| TRINITY_DN10332_c0_g1 | 9.96 | 10.28 | 10.47 | 0.06 | 0 | 0 |  |  |  |  |  |  |  |
| TRINITY_DN5809_c0_g1 | 2.98 | 10.69 | 0 | 1545.53 | 1568.85 | 1521.36 |  |  |  |  |  |  |  |
| TRINITY_DN4578_c0_g1 | 14.91 | 16.35 | 16.14 | 1886.9 | 1951.62 | 1831.19 |  |  |  |  |  |  |  |
| TRINITY_DN1923_c0_g1 | 13.42 | 17.68 | 17.36 | 13.27 | 9.19 | 9.31 |  |  |  |  |  |  |  |
| TRINITY_DN19181_c0_g1 | 4.16 | 15.16 | 6.12 | 653.18 | 662.31 | 449.69 |  |  |  |  |  |  |  |
| TRINITY_DN3417_c0_g3 | 1.25 | 1.28 | 0.96 | 145.74 | 134.89 | 135.28 |  |  |  |  |  |  |  |
| TRINITY_DN6087_c0_g1 | 7.22 | 5.87 | 5.34 | 83.55 | 92.14 | 102.31 |  |  |  |  |  |  |  |
| TRINITY_DN32533_c1_g1 | 48.72 | 51.88 | 47.85 | 2.42 | 2.99 | 3.42 |  |  |  |  |  |  |  |
| TRINITY_DN4224_c0_g1 | 10.58 | 6.69 | 6.84 | 45.28 | 39.11 | 39.74 |  |  |  |  |  |  |  |
| TRINITY_DN3886_c1_g2 | 4.05 | 3.93 | 3.19 | 323.02 | 342.93 | 349.04 |  |  |  |  |  |  |  |
| TRINITY_DN11393_c0_g1 | 24.72 | 21.85 | 25.01 | 7.11 | 8.98 | 8.81 |  |  |  |  |  |  |  |
| TRINITY_DN1339_c3_g1 | 1.23 | 5.99 | 1.17 | 17.28 | 17.93 | 17.02 |  |  |  |  |  |  |  |
| TRINITY_DN16471_c0_g1 | 2.49 | 2.09 | 1.27 | 0.04 | 0.08 | 0.16 |  |  |  |  |  |  |  |
| TRINITY_DN32563_c0_g1 | 3.62 | 3.27 | 4.05 | 245.2 | 230.23 | 243.82 |  |  |  |  |  |  |  |
| TRINITY_DN6367_c0_g2 | 20.93 | 21.84 | 22.38 | 109.51 | 104.47 | 99.94 |  |  |  |  |  |  |  |
| TRINITY_DN1982_c0_g1 | 0.76 | 1.66 | 0.56 | 6.61 | 4.62 | 7.69 |  |  |  |  |  |  |  |
| TRINITY_DN10539_c0_g1 | 28.63 | 28.54 | 24.9 | 150.45 | 147.85 | 142.31 |  |  |  |  |  |  |  |
| TRINITY_DN10803_c0_g1 | 1.17 | 0.92 | 0.8 | 13.7 | 12.06 | 12.65 |  |  |  |  |  |  |  |
| TRINITY_DN23579_c0_g1 | 5.69 | 5.94 | 4.96 | 0.38 | 1.42 | 0.29 |  |  |  |  |  |  |  |
| TRINITY_DN1028_c0_g1 | 14.73 | 17.8 | 17.07 | 3.21 | 1.25 | 7.68 |  |  |  |  |  |  |  |
| TRINITY_DN8873_c0_g1 | 13.29 | 14.68 | 14.09 | 267.52 | 267.09 | 254.32 |  |  |  |  |  |  |  |
| TRINITY_DN8789_c0_g2 | 0.08 | 0 | 0.08 | 1.9 | 1.13 | 1.92 |  |  |  |  |  |  |  |
| TRINITY_DN18925_c1_g1 | 1.9 | 1.24 | 2.16 | 0.16 | 0.32 | 0.59 |  |  |  |  |  |  |  |
| TRINITY_DN5858_c0_g1 | 5.71 | 6.67 | 7.33 | 0.64 | 0.3 | 0.05 |  |  |  |  |  |  |  |
| TRINITY_DN9305_c1_g1 | 0.6 | 0.23 | 0.8 | 11.43 | 11.24 | 12.12 |  |  |  |  |  |  |  |
| TRINITY_DN17578_c0_g1 | 38.28 | 48.82 | 44.48 | 2.18 | 2.18 | 2.07 |  |  |  |  |  |  |  |
| TRINITY_DN2275_c0_g2 | 30.44 | 25.61 | 34.66 | 303.81 | 285.74 | 303.93 |  |  |  |  |  |  |  |
| TRINITY_DN7160_c0_g1 | 0.6 | 0.26 | 0.31 | 9.2 | 9.99 | 7.58 |  |  |  |  |  |  |  |
| TRINITY_DN3145_c1_g1 | 4.04 | 3.78 | 2.49 | 0.15 | 0.15 | 0.16 |  |  |  |  |  |  |  |
| TRINITY_DN3417_c0_g1 | 53.57 | 54.6 | 60.12 | 12826.87 | 11929.5 | 11234.72 |  |  |  |  |  |  |  |
| TRINITY_DN28_c1_g1 | 3.45 | 3.46 | 3.29 | 20.98 | 23.73 | 20.78 |  |  |  |  |  |  |  |
| TRINITY_DN31354_c0_g1 | 1288.81 | 1378.73 | 1317.26 | 94.68 | 85.69 | 77.7 |  |  |  |  |  |  |  |
| TRINITY_DN24312_c1_g1 | 10.13 | 11.19 | 12.59 | 6.41 | 6.31 | 10.23 |  |  |  |  |  |  |  |
| TRINITY_DN12255_c0_g1 | 0.13 | 0.06 | 0.4 | 4.95 | 5.71 | 6.29 |  |  |  |  |  |  |  |
| TRINITY_DN9312_c0_g2 | 3.22 | 2.31 | 2.83 | 90.41 | 93.78 | 85.79 |  |  |  |  |  |  |  |
| TRINITY_DN8789_c0_g1 | 12.6 | 13.49 | 12.86 | 521.77 | 509.42 | 545.58 |  |  |  |  |  |  |  |
| TRINITY_DN8213_c1_g2 | 4.29 | 5.43 | 3.34 | 182.64 | 188.22 | 172.01 |  |  |  |  |  |  |  |
| TRINITY_DN6348_c0_g1 | 5.32 | 5.09 | 6.32 | 99.31 | 110.03 | 101.17 |  |  |  |  |  |  |  |
| TRINITY_DN9_c2_g1 | 25.27 | 28.55 | 29.75 | 165.93 | 175.4 | 161.18 |  |  |  |  |  |  |  |
| TRINITY_DN3204_c0_g2 | 2.13 | 1.99 | 2.7 | 22.02 | 23.38 | 22.06 |  |  |  |  |  |  |  |
| **HQO/LFO group** |  |  |  |  |  |  |  |  |  |  |  |  |  |
| **ID-Gene** | **HQO01** | **HQO02** | **HQO03** | **LFO01** | **LFO02** | **LFO03** | **ID-Meta** | **HQO01** | **HQO02** | **HQO03** | **LFO01** | **LFO02** | **LFO03** |
| **Flavonoid biosynthesis (ko00941)** | | | | | | | | | | | | | |
| **Genes** | | | | | | | **Metabolites** | | | | | | |
| TRINITY_DN632_c0_g1 | 2.42 | 3.74 | 3.07 | 0.66 | 0.61 | 0.45 | Sn-Glycero-3-Phosphocholine | 1550155.62 | 1494730.723 | 1970884.58 | 997435.2639 | 1078410.225 | 1127720.113 |
| TRINITY_DN8455_c0_g1 | 12.75 | 13.01 | 13.05 | 3.51 | 3.88 | 2.97 | Citicoline | 23532.82081 | 28843 | 39080.03494 | 138079.2743 | 91833.88455 | 85834.01926 |
| TRINITY_DN6676_c0_g1 | 89.92 | 96.38 | 126.37 | 174.47 | 143.58 | 127.31 | Sn-Glycerol 3-Phosphate | 786262.706 | 846763.4776 | 878741.3318 | 1844049.857 | 1800326.363 | 1791987.971 |
| TRINITY_DN1095_c0_g4 | 128.49 | 132.95 | 126.48 | 25.72 | 29.59 | 25.76 | L-Serine | 840574.3447 | 842141.9891 | 732526.5147 | 2812208.811 | 2657323.226 | 2703792.312 |
| TRINITY_DN30526_c0_g1 | 13.37 | 12.36 | 8.55 | 0.6 | 0.34 | 0.09 | Phosphorylcholine | 243388.8453 | 300842.6932 | 278677.4421 | 744944.8295 | 842137.3404 | 752599.6929 |
| TRINITY_DN11968_c1_g1 | 212.45 | 195.73 | 212.77 | 27.83 | 27.04 | 24.96 |  |  |  |  |  |  |  |
| TRINITY_DN1372_c0_g3 | 7.07 | 6.29 | 8.66 | 12.02 | 10.13 | 10.63 |  |  |  |  |  |  |  |
| TRINITY_DN14336_c0_g2 | 0.93 | 1.18 | 0.67 | 0.1 | 0.05 | 0.09 |  |  |  |  |  |  |  |
| TRINITY_DN3634_c0_g1 | 1.29 | 1.19 | 1 | 0.4 | 0.11 | 0.25 |  |  |  |  |  |  |  |
| TRINITY_DN7279_c0_g1 | 3.18 | 3.73 | 4.15 | 0.58 | 0.78 | 0.31 |  |  |  |  |  |  |  |
| TRINITY_DN17259_c0_g1 | 1.67 | 1.1 | 1.61 | 0.1 | 0.19 | 0.02 |  |  |  |  |  |  |  |
| TRINITY_DN9438_c0_g1 | 111.22 | 121.36 | 96.63 | 25.36 | 23.83 | 18.16 |  |  |  |  |  |  |  |
| TRINITY_DN8231_c0_g1 | 0.98 | 0.84 | 1.17 | 5.22 | 5.23 | 5.37 |  |  |  |  |  |  |  |
| TRINITY_DN103_c0_g1 | 78.72 | 75.61 | 73.24 | 206.26 | 183.86 | 180.65 |  |  |  |  |  |  |  |
| TRINITY_DN18512_c3_g1 | 6.93 | 6.61 | 8.45 | 0 | 0 | 0 |  |  |  |  |  |  |  |
| TRINITY_DN3207_c0_g1 | 14.79 | 14.95 | 11.21 | 0.91 | 0.95 | 0.63 |  |  |  |  |  |  |  |
| TRINITY_DN9499_c0_g1 | 2.52 | 2.69 | 3.11 | 0.22 | 0.49 | 0.26 |  |  |  |  |  |  |  |
| TRINITY_DN1000_c0_g1 | 4.19 | 4.19 | 3.34 | 0.82 | 0.42 | 0.38 |  |  |  |  |  |  |  |
| TRINITY_DN47617_c0_g1 | 42.72 | 40.2 | 37.62 | 10.52 | 8.45 | 8.24 |  |  |  |  |  |  |  |
| TRINITY_DN4914_c0_g2 | 7.48 | 8.82 | 6.45 | 1.05 | 1.15 | 1.93 |  |  |  |  |  |  |  |
| TRINITY_DN5300_c0_g1 | 5.91 | 6.51 | 6.07 | 10.46 | 9.24 | 10.07 |  |  |  |  |  |  |  |
| TRINITY_DN9053_c0_g1 | 22.74 | 19.27 | 21.56 | 3.01 | 2.31 | 2.28 |  |  |  |  |  |  |  |
| TRINITY_DN9074_c0_g1 | 13.93 | 15.11 | 14.08 | 3.8 | 3.26 | 3.74 |  |  |  |  |  |  |  |
| TRINITY_DN34563_c0_g2 | 7.56 | 6.51 | 7.51 | 0.64 | 0.8 | 0.91 |  |  |  |  |  |  |  |
| TRINITY_DN6179_c0_g1 | 1.13 | 1.72 | 1.63 | 2.78 | 2.87 | 1.82 |  |  |  |  |  |  |  |
| TRINITY_DN38079_c0_g1 | 0.8 | 1.02 | 0.38 | 1.43 | 2.58 | 2.16 |  |  |  |  |  |  |  |
| TRINITY_DN2760_c1_g1 | 6.01 | 5.33 | 5.54 | 2.22 | 2.46 | 1.39 |  |  |  |  |  |  |  |
| TRINITY_DN28081_c0_g1 | 2.94 | 1.48 | 1.79 | 0.06 | 0.13 | 0.11 |  |  |  |  |  |  |  |
| TRINITY_DN6461_c0_g1 | 1.35 | 1.44 | 1.23 | 0 | 0.07 | 0.15 |  |  |  |  |  |  |  |
| TRINITY_DN5134_c0_g1 | 240.71 | 238.33 | 251 | 58.68 | 56.94 | 47.78 |  |  |  |  |  |  |  |
| TRINITY_DN14900_c0_g1 | 26.54 | 24.96 | 25.78 | 8.66 | 13.49 | 6.49 |  |  |  |  |  |  |  |

**Table S4 The KEGG enrichment analysis of DAMs within EATO/HQO and EATO/LFO group**

| **ID** | **Description** | **MetaboliteRatio** | **BgRatio** | **enrich_factor** | **pvalue** | **metaboliteID** | **Count** | **regulated** |
| --- | --- | --- | --- | --- | --- | --- | --- | --- |
| **EATO/HQO** | | | | | | | | |
| ko00460 | Cyanoamino acid metabolism | 5.83% | 3.17% | 1.84 | 0.012817085 | NEG_q267;NEG_q277;NEG_q280;POS_q215;POS_q229;POS_q237;POS_q243 | 7 | down |
| ko00970 | Aminoacyl-tRNA biosynthesis | 10.83% | 7.24% | 1.5 | 0.020830998 | NEG_q267;NEG_q277;NEG_q279;NEG_q280;POS_q215;POS_q221;POS_q222;POS_q229;POS_q230;POS_q232;POS_q237;POS_q239;POS_q243 | 13 | up&down |
| ko00966 | Glucosinolate biosynthesis | 5% | 2.71% | 1.84 | 0.024172572 | NEG_q280;NEG_t83;POS_q229;POS_q230;POS_q237;POS_q243 | 6 | up&down |
| ko00290 | Valine, leucine and isoleucine biosynthesis | 4.17% | 2.26% | 1.84 | 0.045402396 | NEG_q129;NEG_q279;POS_q229;POS_q230;POS_q243 | 5 | down |
| ko00920 | Sulfur metabolism | 4.17% | 2.26% | 1.84 | 0.045402396 | NEG_q277;NEG_q405;NEG_q407;NEG_q411;POS_q223 | 5 | down |
| ko00630 | Glyoxylate and dicarboxylate metabolism | 5.83% | 3.62% | 1.61 | 0.055161378 | NEG_q216;NEG_q277;NEG_q278;NEG_q286;NEG_q341;NEG_q405;POS_q221 | 7 | up&down |
| ko00470 | D-Amino acid metabolism | 10% | 7.24% | 1.38 | 0.069337922 | NEG_q267;NEG_q277;NEG_q279;POS_q123;POS_q141;POS_q142;POS_q144;POS_q221;POS_q222;POS_q232;POS_q237;POS_q239 | 12 | up&down |
| ko00950 | Isoquinoline alkaloid biosynthesis | 3.33% | 1.81% | 1.84 | 0.084933792 | NEG_q280;NEG_t312;NEG_t55;POS_q357 | 4 | down |
| ko00940 | Phenylpropanoid biosynthesis | 6.67% | 4.52% | 1.47 | 0.086937147 | NEG_q106;NEG_q133;NEG_q201;NEG_q280;NEG_q348;NEG_q349;NEG_t31;POS_q237 | 8 | up&down |
| ko00260 | Glycine, serine and threonine metabolism | 5% | 3.17% | 1.58 | 0.092305495 | NEG_q263;NEG_q267;NEG_q277;NEG_q279;POS_q144;POS_q223 | 6 | down |
| ko00261 | Monobactam biosynthesis | 4.17% | 2.71% | 1.53 | 0.151551515 | NEG_q267;NEG_q277;NEG_q279;NEG_q280;NEG_q407 | 5 | down |
| ko00073 | Cutin, suberine and wax biosynthesis | 2.5% | 1.36% | 1.84 | 0.158252706 | NEG_q224;NEG_q337;NEG_q345 | 3 | up&down |
| ko00300 | Lysine biosynthesis | 2.5% | 1.36% | 1.84 | 0.158252706 | NEG_q267;POS_q223;POS_q232 | 3 | down |
| ko00941 | Flavonoid biosynthesis | 10% | 8.14% | 1.23 | 0.19786944 | NEG_q158;NEG_q160;NEG_q207;NEG_q256;NEG_q313;NEG_q321;NEG_q348;NEG_q353;NEG_q354;NEG_q424;NEG_q93;POS_q148 | 12 | up&down |
| ko00270 | Cysteine and methionine metabolism | 5% | 3.62% | 1.38 | 0.203737846 | NEG_q267;NEG_q277;NEG_q407;POS_q223;POS_q4;POS_q56 | 6 | up&down |
| ko00040 | Pentose and glucuronate interconversions | 3.33% | 2.26% | 1.47 | 0.243059377 | NEG_q140;NEG_q264;NEG_q382;NEG_t348 | 4 | down |
| ko00280 | Valine, leucine and isoleucine degradation | 3.33% | 2.26% | 1.47 | 0.243059377 | NEG_q302;POS_q229;POS_q230;POS_q243 | 4 | down |
| ko00564 | Glycerophospholipid metabolism | 3.33% | 2.26% | 1.47 | 0.243059377 | NEG_q277;NEG_q400;POS_q298;POS_q335 | 4 | up&down |
| ko00944 | Flavone and flavonol biosynthesis | 6.67% | 5.43% | 1.23 | 0.281546241 | NEG_q248;NEG_q253;NEG_q256;NEG_q259;NEG_q313;NEG_q81;NEG_q93;POS_q83 | 8 | up&down |
| ko00020 | Citrate cycle (TCA cycle) | 1.67% | 0.9% | 1.84 | 0.293706294 | NEG_q286;NEG_q405 | 2 | up&down |
| ko00071 | Fatty acid degradation | 1.67% | 0.9% | 1.84 | 0.293706294 | NEG_q215;NEG_q345 | 2 | up&down |
| ko00620 | Pyruvate metabolism | 1.67% | 0.9% | 1.84 | 0.293706294 | NEG_q286;NEG_q405 | 2 | up&down |
| ko00640 | Propanoate metabolism | 1.67% | 0.9% | 1.84 | 0.293706294 | NEG_q302;NEG_q405 | 2 | down |
| ko00770 | Pantothenate and CoA biosynthesis | 1.67% | 0.9% | 1.84 | 0.293706294 | NEG_q267;POS_q243 | 2 | down |
| ko00780 | Biotin metabolism | 1.67% | 0.9% | 1.84 | 0.293706294 | NEG_q361;POS_q232 | 2 | down |
| ko00360 | Phenylalanine metabolism | 4.17% | 3.17% | 1.32 | 0.299666566 | NEG_q280;NEG_q405;POS_q141;POS_q237;POS_q29 | 5 | down |
| ko00500 | Starch and sucrose metabolism | 4.17% | 3.17% | 1.32 | 0.299666566 | NEG_q149;NEG_q238;NEG_q243;NEG_q406;NEG_t1 | 5 | down |
| ko00130 | Ubiquinone and other terpenoid-quinone biosynthesis | 2.5% | 1.81% | 1.38 | 0.378209449 | NEG_q280;NEG_q58;NEG_t314 | 3 | up&down |
| ko00340 | Histidine metabolism | 2.5% | 1.81% | 1.38 | 0.378209449 | NEG_q267;POS_q222;POS_q81 | 3 | up&down |
| ko00400 | Phenylalanine, tyrosine and tryptophan biosynthesis | 2.5% | 1.81% | 1.38 | 0.378209449 | NEG_q280;NEG_t265;POS_q237 | 3 | down |
| ko00410 | beta-Alanine metabolism | 2.5% | 1.81% | 1.38 | 0.378209449 | NEG_q267;POS_q222;POS_q81 | 3 | up&down |
| ko00591 | Linoleic acid metabolism | 2.5% | 1.81% | 1.38 | 0.378209449 | NEG_t205;POS_q1;POS_q68 | 3 | up |
| ko00760 | Nicotinate and nicotinamide metabolism | 2.5% | 1.81% | 1.38 | 0.378209449 | NEG_q267;NEG_q325;NEG_q405 | 3 | down |
| ko00220 | Arginine biosynthesis | 3.33% | 2.71% | 1.23 | 0.4260751 | NEG_q267;NEG_q316;POS_q217;POS_q221 | 4 | down |
| ko00250 | Alanine, aspartate and glutamate metabolism | 3.33% | 2.71% | 1.23 | 0.4260751 | NEG_q267;NEG_q405;POS_q215;POS_q221 | 4 | down |
| ko00350 | Tyrosine metabolism | 3.33% | 2.71% | 1.23 | 0.4260751 | NEG_q280;NEG_q405;NEG_t312;POS_q357 | 4 | down |
| ko02010 | ABC transporters | 18.33% | 17.65% | 1.04 | 0.455753162 | NEG_q179;NEG_q237;NEG_q25;NEG_q267;NEG_q277;NEG_q279;NEG_q400;NEG_q406;NEG_q407;NEG_q411;NEG_q428;NEG_t1;POS_q22;POS_q221;POS_q222;POS_q229;POS_q230;POS_q232;POS_q237;POS_q239;POS_q243;POS_q71 | 22 | up&down |
| ko00240 | Pyrimidine metabolism | 4.17% | 3.62% | 1.15 | 0.459547765 | NEG_q302;NEG_q339;NEG_q428;NEG_q431;POS_q221 | 5 | up&down |
| ko00230 | Purine metabolism | 5.83% | 5.43% | 1.07 | 0.506885998 | NEG_q237;NEG_q25;NEG_q341;NEG_q407;POS_q22;POS_q221;POS_q71 | 7 | up&down |
| ko00030 | Pentose phosphate pathway | 0.83% | 0.45% | 1.84 | 0.542986425 | NEG_q147 | 1 | down |
| ko00062 | Fatty acid elongation | 0.83% | 0.45% | 1.84 | 0.542986425 | NEG_q345 | 1 | up |
| ko00100 | Steroid biosynthesis | 0.83% | 0.45% | 1.84 | 0.542986425 | POS_q119 | 1 | up |
| ko00190 | Oxidative phosphorylation | 0.83% | 0.45% | 1.84 | 0.542986425 | NEG_q405 | 1 | down |
| ko00561 | Glycerolipid metabolism | 0.83% | 0.45% | 1.84 | 0.542986425 | NEG_q400 | 1 | up |
| ko00565 | Ether lipid metabolism | 0.83% | 0.45% | 1.84 | 0.542986425 | POS_q335 | 1 | down |
| ko00600 | Sphingolipid metabolism | 0.83% | 0.45% | 1.84 | 0.542986425 | NEG_q277 | 1 | down |
| ko00730 | Thiamine metabolism | 0.83% | 0.45% | 1.84 | 0.542986425 | NEG_q280 | 1 | down |
| ko00901 | Indole alkaloid biosynthesis | 0.83% | 0.45% | 1.84 | 0.542986425 | NEG_t17 | 1 | down |
| ko00945 | Stilbenoid, diarylheptanoid and gingerol biosynthesis | 0.83% | 0.45% | 1.84 | 0.542986425 | NEG_q348 | 1 | up |
| ko00053 | Ascorbate and aldarate metabolism | 1.67% | 1.36% | 1.23 | 0.564613469 | NEG_q140;NEG_q312 | 2 | down |
| ko01040 | Biosynthesis of unsaturated fatty acids | 1.67% | 1.36% | 1.23 | 0.564613469 | NEG_q337;NEG_q345 | 2 | up |
| ko00996 | Biosynthesis of various alkaloids | 2.5% | 2.26% | 1.1 | 0.580934558 | NEG_q101;POS_q237;POS_q360 | 3 | up&down |
| ko00943 | Isoflavonoid biosynthesis | 4.17% | 4.07% | 1.02 | 0.606668493 | NEG_q208;NEG_q28;NEG_q365;NEG_q93;NEG_t36 | 5 | up&down |
| ko00999 | Biosynthesis of various plant secondary metabolites | 4.17% | 4.52% | 0.92 | 0.727834904 | NEG_q136;NEG_q204;NEG_q267;NEG_q280;POS_q237 | 5 | up&down |
| ko00310 | Lysine degradation | 2.5% | 2.71% | 0.92 | 0.735794016 | NEG_q215;NEG_q405;POS_q232 | 3 | down |
| ko00592 | alpha-Linolenic acid metabolism | 2.5% | 2.71% | 0.92 | 0.735794016 | NEG_q422;NEG_t191;NEG_t231 | 3 | up |
| ko00960 | Tropane, piperidine and pyridine alkaloid biosynthesis | 2.5% | 2.71% | 0.92 | 0.735794016 | POS_q229;POS_q232;POS_q237 | 3 | down |
| ko00061 | Fatty acid biosynthesis | 1.67% | 1.81% | 0.92 | 0.751017488 | NEG_q337;NEG_q345 | 2 | up |
| ko00330 | Arginine and proline metabolism | 1.67% | 1.81% | 0.92 | 0.751017488 | POS_q142;POS_q239 | 2 | down |
| ko00562 | Inositol phosphate metabolism | 1.67% | 1.81% | 0.92 | 0.751017488 | NEG_q149;NEG_q6 | 2 | up&down |
| ko00710 | Carbon fixation in photosynthetic organisms | 1.67% | 1.81% | 0.92 | 0.751017488 | NEG_q267;NEG_q286 | 2 | up&down |
| ko00254 | Aflatoxin biosynthesis | 0.83% | 0.9% | 0.92 | 0.792266557 | NEG_t12 | 1 | down |
| ko00311 | Penicillin and cephalosporin biosynthesis | 0.83% | 0.9% | 0.92 | 0.792266557 | POS_q243 | 1 | down |
| ko00380 | Tryptophan metabolism | 0.83% | 0.9% | 0.92 | 0.792266557 | POS_q206 | 1 | up |
| ko00440 | Phosphonate and phosphinate metabolism | 0.83% | 0.9% | 0.92 | 0.792266557 | NEG_q356 | 1 | down |
| ko00740 | Riboflavin metabolism | 0.83% | 0.9% | 0.92 | 0.792266557 | NEG_q382 | 1 | down |
| ko00790 | Folate biosynthesis | 0.83% | 0.9% | 0.92 | 0.792266557 | NEG_q58 | 1 | up |
| ko00900 | Terpenoid backbone biosynthesis | 0.83% | 0.9% | 0.92 | 0.792266557 | NEG_t266 | 1 | up |
| ko00906 | Carotenoid biosynthesis | 0.83% | 0.9% | 0.92 | 0.792266557 | NEG_q75 | 1 | up |
| ko00908 | Zeatin biosynthesis | 0.83% | 0.9% | 0.92 | 0.792266557 | POS_q56 | 1 | up |
| ko00910 | Nitrogen metabolism | 0.83% | 0.9% | 0.92 | 0.792266557 | POS_q221 | 1 | down |
| ko00965 | Betalain biosynthesis | 0.83% | 0.9% | 0.92 | 0.792266557 | NEG_q280 | 1 | down |
| ko04070 | Phosphatidylinositol signaling system | 0.83% | 0.9% | 0.92 | 0.792266557 | NEG_q6 | 1 | up |
| ko04075 | Plant hormone signal transduction | 0.83% | 0.9% | 0.92 | 0.792266557 | NEG_q75 | 1 | up |
| ko00524 | Neomycin, kanamycin and gentamicin biosynthesis | 1.67% | 2.26% | 0.74 | 0.864406108 | NEG_q149;NEG_t344 | 2 | down |
| ko00010 | Glycolysis / Gluconeogenesis | 0.83% | 1.36% | 0.61 | 0.906093101 | NEG_q94 | 1 | down |
| ko00332 | Carbapenem biosynthesis | 0.83% | 1.36% | 0.61 | 0.906093101 | POS_q239 | 1 | down |
| ko00650 | Butanoate metabolism | 0.83% | 1.36% | 0.61 | 0.906093101 | NEG_q405 | 1 | down |
| ko00660 | C5-Branched dibasic acid metabolism | 0.83% | 1.36% | 0.61 | 0.906093101 | NEG_q129 | 1 | down |
| ko00750 | Vitamin B6 metabolism | 0.83% | 1.36% | 0.61 | 0.906093101 | POS_q221 | 1 | down |
| ko00904 | Diterpenoid biosynthesis | 0.83% | 1.36% | 0.61 | 0.906093101 | NEG_q245 | 1 | up |
| ko00520 | Amino sugar and nucleotide sugar metabolism | 1.67% | 2.71% | 0.61 | 0.928712155 | NEG_q140;POS_q273 | 2 | down |
| ko00430 | Taurine and hypotaurine metabolism | 0.83% | 1.81% | 0.46 | 0.957784972 | NEG_q411 | 1 | down |
| ko00051 | Fructose and mannose metabolism | 1.67% | 3.17% | 0.53 | 0.963527149 | NEG_q179;POS_q95 | 2 | up&down |
| ko00860 | Porphyrin metabolism | 0.83% | 2.26% | 0.37 | 0.981129688 | NEG_q279 | 1 | down |
| ko00052 | Galactose metabolism | 3.33% | 6.33% | 0.53 | 0.989152542 | NEG_q179;NEG_q406;NEG_t45;POS_q249 | 4 | up&down |
| **EATO/LFO** | | | | | | | | |
| ko00941 | Flavonoid biosynthesis | 12.15% | 8.14% | 1.49 | 0.030366261 | NEG_q158;NEG_q160;NEG_q207;NEG_q313;NEG_q348;NEG_q353;NEG_q354;NEG_q362;NEG_q412;NEG_q413;NEG_q424;POS_q148;POS_q296 | 13 | up&down |
| ko00260 | Glycine, serine and threonine metabolism | 5.61% | 3.17% | 1.77 | 0.050008274 | NEG_q263;NEG_q277;NEG_q279;POS_q144;POS_q223;POS_q52 | 6 | up |
| ko00400 | Phenylalanine, tyrosine and tryptophan biosynthesis | 3.74% | 1.81% | 2.07 | 0.053357653 | NEG_q280;NEG_q397;NEG_t265;POS_q237 | 4 | up&down |
| ko00950 | Isoquinoline alkaloid biosynthesis | 3.74% | 1.81% | 2.07 | 0.053357653 | NEG_q280;NEG_t312;NEG_t55;POS_q357 | 4 | down |
| ko00470 | D-Amino acid metabolism | 10.28% | 7.24% | 1.42 | 0.075789633 | NEG_q270;NEG_q277;NEG_q279;POS_q123;POS_q141;POS_q142;POS_q144;POS_q221;POS_q232;POS_q237;POS_q239 | 11 | up&down |
| ko00970 | Aminoacyl-tRNA biosynthesis | 10.28% | 7.24% | 1.42 | 0.075789633 | NEG_q270;NEG_q277;NEG_q279;NEG_q280;POS_q215;POS_q221;POS_q229;POS_q230;POS_q232;POS_q237;POS_q239 | 11 | up&down |
| ko00350 | Tyrosine metabolism | 4.67% | 2.71% | 1.72 | 0.092160114 | NEG_q280;NEG_q405;NEG_t312;NEG_t44;POS_q357 | 5 | up&down |
| ko00966 | Glucosinolate biosynthesis | 4.67% | 2.71% | 1.72 | 0.092160114 | NEG_q280;NEG_t83;POS_q229;POS_q230;POS_q237 | 5 | up&down |
| ko00073 | Cutin, suberine and wax biosynthesis | 2.8% | 1.36% | 2.07 | 0.11184585 | NEG_q224;NEG_q337;NEG_q345 | 3 | up&down |
| ko00660 | C5-Branched dibasic acid metabolism | 2.8% | 1.36% | 2.07 | 0.11184585 | NEG_q129;NEG_q270;NEG_q420 | 3 | up&down |
| ko00240 | Pyrimidine metabolism | 5.61% | 3.62% | 1.55 | 0.120326998 | NEG_q287;NEG_q339;NEG_q428;NEG_q431;POS_q121;POS_q221 | 6 | up&down |
| ko00630 | Glyoxylate and dicarboxylate metabolism | 5.61% | 3.62% | 1.55 | 0.120326998 | NEG_q270;NEG_q277;NEG_q278;NEG_q341;NEG_q405;POS_q221 | 6 | up&down |
| ko00290 | Valine, leucine and isoleucine biosynthesis | 3.74% | 2.26% | 1.65 | 0.165482491 | NEG_q129;NEG_q279;POS_q229;POS_q230 | 4 | up&down |
| ko00564 | Glycerophospholipid metabolism | 3.74% | 2.26% | 1.65 | 0.165482491 | NEG_q277;NEG_q400;POS_q298;POS_q335 | 4 | up&down |
| ko00460 | Cyanoamino acid metabolism | 4.67% | 3.17% | 1.48 | 0.197539716 | NEG_q277;NEG_q280;POS_q215;POS_q229;POS_q237 | 5 | up&down |
| ko00500 | Starch and sucrose metabolism | 4.67% | 3.17% | 1.48 | 0.197539716 | NEG_q149;NEG_q238;NEG_q243;NEG_q406;NEG_t1 | 5 | down |
| ko00071 | Fatty acid degradation | 1.87% | 0.9% | 2.07 | 0.233278486 | NEG_q215;NEG_q345 | 2 | up&down |
| ko00780 | Biotin metabolism | 1.87% | 0.9% | 2.07 | 0.233278486 | NEG_q361;POS_q232 | 2 | down |
| ko00910 | Nitrogen metabolism | 1.87% | 0.9% | 2.07 | 0.233278486 | NEG_q270;POS_q221 | 2 | down |
| ko00061 | Fatty acid biosynthesis | 2.8% | 1.81% | 1.55 | 0.287310439 | NEG_q287;NEG_q337;NEG_q345 | 3 | up&down |
| ko00130 | Ubiquinone and other terpenoid-quinone biosynthesis | 2.8% | 1.81% | 1.55 | 0.287310439 | NEG_q280;NEG_q58;NEG_t314 | 3 | up&down |
| ko00330 | Arginine and proline metabolism | 2.8% | 1.81% | 1.55 | 0.287310439 | NEG_q270;POS_q142;POS_q239 | 3 | down |
| ko00562 | Inositol phosphate metabolism | 2.8% | 1.81% | 1.55 | 0.287310439 | NEG_q149;NEG_q6;NEG_t360 | 3 | up&down |
| ko00591 | Linoleic acid metabolism | 2.8% | 1.81% | 1.55 | 0.287310439 | NEG_t205;POS_q1;POS_q68 | 3 | up |
| ko00250 | Alanine, aspartate and glutamate metabolism | 3.74% | 2.71% | 1.38 | 0.312127244 | NEG_q270;NEG_q405;POS_q215;POS_q221 | 4 | up&down |
| ko00940 | Phenylpropanoid biosynthesis | 5.61% | 4.52% | 1.24 | 0.335084914 | NEG_q106;NEG_q133;NEG_q280;NEG_q348;NEG_t31;POS_q237 | 6 | up&down |
| ko00999 | Biosynthesis of various plant secondary metabolites | 5.61% | 4.52% | 1.24 | 0.335084914 | NEG_q136;NEG_q204;NEG_q250;NEG_q280;NEG_q397;POS_q237 | 6 | up&down |
| ko00360 | Phenylalanine metabolism | 3.74% | 3.17% | 1.18 | 0.464910614 | NEG_q280;NEG_q405;POS_q141;POS_q237 | 4 | up&down |
| ko00524 | Neomycin, kanamycin and gentamicin biosynthesis | 2.8% | 2.26% | 1.24 | 0.470052362 | NEG_q149;NEG_q270;NEG_t344 | 3 | down |
| ko00860 | Porphyrin metabolism | 2.8% | 2.26% | 1.24 | 0.470052362 | NEG_q270;NEG_q279;POS_q52 | 3 | up&down |
| ko00920 | Sulfur metabolism | 2.8% | 2.26% | 1.24 | 0.470052362 | NEG_q277;NEG_q405;POS_q223 | 3 | up |
| ko00996 | Biosynthesis of various alkaloids | 2.8% | 2.26% | 1.24 | 0.470052362 | NEG_q101;NEG_q397;POS_q237 | 3 | up&down |
| ko00300 | Lysine biosynthesis | 1.87% | 1.36% | 1.38 | 0.47614376 | POS_q223;POS_q232 | 2 | up&down |
| ko00332 | Carbapenem biosynthesis | 1.87% | 1.36% | 1.38 | 0.47614376 | NEG_q270;POS_q239 | 2 | down |
| ko00650 | Butanoate metabolism | 1.87% | 1.36% | 1.38 | 0.47614376 | NEG_q270;NEG_q405 | 2 | up&down |
| ko00750 | Vitamin B6 metabolism | 1.87% | 1.36% | 1.38 | 0.47614376 | NEG_q62;POS_q221 | 2 | up&down |
| ko01040 | Biosynthesis of unsaturated fatty acids | 1.87% | 1.36% | 1.38 | 0.47614376 | NEG_q337;NEG_q345 | 2 | up |
| ko00062 | Fatty acid elongation | 0.93% | 0.45% | 2.07 | 0.484162896 | NEG_q345 | 1 | up |
| ko00100 | Steroid biosynthesis | 0.93% | 0.45% | 2.07 | 0.484162896 | POS_q119 | 1 | up |
| ko00190 | Oxidative phosphorylation | 0.93% | 0.45% | 2.07 | 0.484162896 | NEG_q405 | 1 | up |
| ko00232 | Caffeine metabolism | 0.93% | 0.45% | 2.07 | 0.484162896 | NEG_q443 | 1 | up |
| ko00480 | Glutathione metabolism | 0.93% | 0.45% | 2.07 | 0.484162896 | NEG_q270 | 1 | down |
| ko00561 | Glycerolipid metabolism | 0.93% | 0.45% | 2.07 | 0.484162896 | NEG_q400 | 1 | up |
| ko00565 | Ether lipid metabolism | 0.93% | 0.45% | 2.07 | 0.484162896 | POS_q335 | 1 | down |
| ko00600 | Sphingolipid metabolism | 0.93% | 0.45% | 2.07 | 0.484162896 | NEG_q277 | 1 | up |
| ko00730 | Thiamine metabolism | 0.93% | 0.45% | 2.07 | 0.484162896 | NEG_q280 | 1 | down |
| ko00901 | Indole alkaloid biosynthesis | 0.93% | 0.45% | 2.07 | 0.484162896 | NEG_t17 | 1 | down |
| ko00945 | Stilbenoid, diarylheptanoid and gingerol biosynthesis | 0.93% | 0.45% | 2.07 | 0.484162896 | NEG_q348 | 1 | up |
| ko00270 | Cysteine and methionine metabolism | 3.74% | 3.62% | 1.03 | 0.60359365 | NEG_q277;POS_q223;POS_q4;POS_q56 | 4 | up |
| ko00220 | Arginine biosynthesis | 2.8% | 2.71% | 1.03 | 0.627977481 | NEG_q270;NEG_q316;POS_q221 | 3 | up&down |
| ko00261 | Monobactam biosynthesis | 2.8% | 2.71% | 1.03 | 0.627977481 | NEG_q277;NEG_q279;NEG_q280 | 3 | up&down |
| ko00310 | Lysine degradation | 2.8% | 2.71% | 1.03 | 0.627977481 | NEG_q215;NEG_q405;POS_q232 | 3 | up&down |
| ko00592 | alpha-Linolenic acid metabolism | 2.8% | 2.71% | 1.03 | 0.627977481 | NEG_q422;NEG_t191;NEG_t231 | 3 | up |
| ko00960 | Tropane, piperidine and pyridine alkaloid biosynthesis | 2.8% | 2.71% | 1.03 | 0.627977481 | POS_q229;POS_q232;POS_q237 | 3 | up&down |
| ko00760 | Nicotinate and nicotinamide metabolism | 1.87% | 1.81% | 1.03 | 0.66497708 | NEG_q325;NEG_q405 | 2 | up&down |
| ko00943 | Isoflavonoid biosynthesis | 3.74% | 4.07% | 0.92 | 0.718186206 | NEG_q208;NEG_q28;NEG_q365;NEG_t36 | 4 | up&down |
| ko00020 | Citrate cycle (TCA cycle) | 0.93% | 0.9% | 1.03 | 0.735047306 | NEG_q405 | 1 | up |
| ko00254 | Aflatoxin biosynthesis | 0.93% | 0.9% | 1.03 | 0.735047306 | NEG_t12 | 1 | down |
| ko00440 | Phosphonate and phosphinate metabolism | 0.93% | 0.9% | 1.03 | 0.735047306 | NEG_q356 | 1 | down |
| ko00620 | Pyruvate metabolism | 0.93% | 0.9% | 1.03 | 0.735047306 | NEG_q405 | 1 | up |
| ko00640 | Propanoate metabolism | 0.93% | 0.9% | 1.03 | 0.735047306 | NEG_q405 | 1 | up |
| ko00790 | Folate biosynthesis | 0.93% | 0.9% | 1.03 | 0.735047306 | NEG_q58 | 1 | up |
| ko00906 | Carotenoid biosynthesis | 0.93% | 0.9% | 1.03 | 0.735047306 | NEG_q75 | 1 | up |
| ko00908 | Zeatin biosynthesis | 0.93% | 0.9% | 1.03 | 0.735047306 | POS_q56 | 1 | up |
| ko00942 | Anthocyanin biosynthesis | 0.93% | 0.9% | 1.03 | 0.735047306 | POS_q296 | 1 | up |
| ko00965 | Betalain biosynthesis | 0.93% | 0.9% | 1.03 | 0.735047306 | NEG_q280 | 1 | down |
| ko04070 | Phosphatidylinositol signaling system | 0.93% | 0.9% | 1.03 | 0.735047306 | NEG_q6 | 1 | up |
| ko04075 | Plant hormone signal transduction | 0.93% | 0.9% | 1.03 | 0.735047306 | NEG_q75 | 1 | up |
| ko00230 | Purine metabolism | 4.67% | 5.43% | 0.86 | 0.780637211 | NEG_q237;NEG_q341;NEG_q443;POS_q221;POS_q71 | 5 | up&down |
| ko00280 | Valine, leucine and isoleucine degradation | 1.87% | 2.26% | 0.83 | 0.794926892 | POS_q229;POS_q230 | 2 | up |
| ko00010 | Glycolysis / Gluconeogenesis | 0.93% | 1.36% | 0.69 | 0.864499079 | NEG_q94 | 1 | down |
| ko00053 | Ascorbate and aldarate metabolism | 0.93% | 1.36% | 0.69 | 0.864499079 | NEG_q312 | 1 | up |
| ko00520 | Amino sugar and nucleotide sugar metabolism | 1.87% | 2.71% | 0.69 | 0.878401597 | NEG_t352;POS_q273 | 2 | up&down |
| ko02010 | ABC transporters | 14.95% | 17.65% | 0.85 | 0.884042478 | NEG_q237;NEG_q270;NEG_q277;NEG_q279;NEG_q400;NEG_q406;NEG_q428;NEG_t1;POS_q221;POS_q229;POS_q230;POS_q232;POS_q237;POS_q239;POS_q52;POS_q71 | 16 | up&down |
| ko00944 | Flavone and flavonol biosynthesis | 3.74% | 5.43% | 0.69 | 0.916459172 | NEG_q248;NEG_q313;NEG_q81;POS_q107 | 4 | up&down |
| ko00340 | Histidine metabolism | 0.93% | 1.81% | 0.52 | 0.931006412 | NEG_q270 | 1 | down |
| ko00410 | beta-Alanine metabolism | 0.93% | 1.81% | 0.52 | 0.931006412 | NEG_q287 | 1 | down |
| ko00430 | Taurine and hypotaurine metabolism | 0.93% | 1.81% | 0.52 | 0.931006412 | NEG_q270 | 1 | down |
| ko00710 | Carbon fixation in photosynthetic organisms | 0.93% | 1.81% | 0.52 | 0.931006412 | NEG_t47 | 1 | up |
| ko00040 | Pentose and glucuronate interconversions | 0.93% | 2.26% | 0.41 | 0.965026292 | NEG_t348 | 1 | up |
| ko00051 | Fructose and mannose metabolism | 0.93% | 3.17% | 0.3 | 0.991134572 | POS_q95 | 1 | up |
| ko00052 | Galactose metabolism | 2.8% | 6.33% | 0.44 | 0.992737996 | NEG_q406;NEG_t352;NEG_t45 | 3 | up&down |
